# The oligosaccharyltransferase TaOST1B promotes viral infection by enhancement of RNA silencing suppression in wheat

**DOI:** 10.1038/s41467-026-75397-8

**Published:** 2026-07-15

**Authors:** Jiaqian Liu, Xia Wang, Ying Liu, Jiaqian Yang, Chaonan Shi, Bin Yong, Yingjie Zhao, Yaoyao Jiang, Haichao Hu, Lixiao Feng, Jun Guo, Tianye Zhang, Kaili Zhong, Peng Liu, Keqi Tang, Feng Chen, Jianping Chen, Jian Yang

**Affiliations:** 1 State Key Laboratory for Quality and Safety of Agro-Products, Key Laboratory of Biotechnology in Plant Protection of MARA, Zhejiang Key Laboratory of Green Plant Protection, Institute of Plant Virology, Ningbo University, Ningbo, China; 2 Institute of Mass Spectrometry, Zhejiang Engineering Research Center of Advanced Mass Spectrometry and Clinical Application, School of Material Science and Chemical Engineering, Ningbo University, Ningbo, China; 3 National Key Laboratory of Wheat and Maize Crop Science/CIMMYT-China Wheat and Maize Joint Research Center/Agronomy College, Henan Agricultural University, Zhengzhou, China

**Keywords:** Virulence, Microbe, Plant cell biology

## Abstract

Viral suppressors of RNA silencing (VSRs) are crucial for viral infection. Here, we show that a wheat (*Triticum aestivum*) oligosaccharyltransferase (*TaOST1B*) is associated with resistance to Wheat yellow mosaic virus (WYMV). TaOST1B interacts with WYMV-encoded P1 and enhances the VSR function of P1 by N-glycosylating its asparagine residue at position 116. This increases intranuclear accumulation of P1 through interaction with the nuclear transport protein TaIMP-α2 and blocks the interaction between calmodulin (CaM3) and CaM-binding transcriptional activator (CaMTA3) to suppress RNA interference. Nevertheless, nonglycosylated P1 loses its VSR function and forms aggregates triggering endoplasmic reticulum (ER) stress and is subsequently degraded by the 26S proteasome. A natural variant of TaOST1B fails to bind P1 and regulate its N-glycosylation, which induces ER stress and proteasome-mediated degradation to attenuate WYMV infection. Our study identifies an oligosaccharyltransferases as being utilized by VSR to promote viral infection, offering insights into the arms race between plants and viruses.

## Abstract (web summary)

Viral suppressors of RNA silencing promote viral infection. Here, the authors show that *N*-glycosylation of the wheat yellow mosaic virus P1 by host TaOST1B enhances nuclear localization and virulence.

## Introduction

Plants typically mount a defense response against infection after sensing viral invasion. RNA interference (RNAi) is a fundamental and conserved antiviral immune response in plants. In general, RNA-induced silencing complexes could inhibit viral infection via transcriptional or posttranscriptional gene silencing^1^. However, viruses have evolved viral suppressors of RNA silencing (VSRs) to counter host antiviral defenses. For instance, P19 of tombusvirus^2^, helper component proteinase of Potyvirus, P25 of potato virus^3^, 2b of cucumovirus^4^. P0 of Polerovirus^5^, and V2 of tomato yellow leaf curl virus (TYLCV)^6,7^. Because RNA viruses are obligate intracellular infectious pathogens that lack enzymes capable of introducing posttranslational modifications (PTMs) in VSRs or other viral proteins, they usually utilize the host PTM machinery to promote their survival. Barley stripe mosaic virus (BSMV)-encoded γb protein can be palmitoylated by the palmitoyltransferases NbPAT15 and NbPAT21 to interact with NbREM1, enlarging the pore size of the cellular plasmodesmata by inhibiting callose deposition in the plasmodesmata, further promoting viral movement^8^. The S-adenosylmethionine decarboxylase SAMDC3 can interact with the BSMV-encoded γb protein to promote the ubiquitination and proteasomal degradation of γb^9^. Chinese wheat mosaic virus (CWMV)-encoded cysteine-rich protein can be phosphorylated by the host serine/threonine protein kinase SAPK7, which suppresses cell death and H_2_O_2_ production via the RNA-binding protein UBP1-associated protein 2 C to enhance virus infection^10^. Therefore, PTMs are essential for viral infection and plant immunity.

Glycosylation, an important and widespread PTM in eukaryotes, plays a crucial role in maintaining proper protein folding, recognition, development, signal transduction, the immune response, and metabolism^11–14^. N-glycosylation is the process by which the preformed oligosaccharide is transferred onto the asparagine residue of the conserved N-X-S/T motif (where X is any amino acid other than proline) in the nascent polypeptide via the oligosaccharyltransferase (OST) complex in the endoplasmic reticulum (ER) lumen^15–17^. Almost 70% of proteins with N-X-S/T sequences are glycoproteins^18^. In bacteria and some lower eukaryotes, this process is catalyzed by a single-subunit oligosaccharyltransferase^19^, while most eukaryote-encoded OST complexes are composed of multiple subunits^20,21^. The polypeptides linked to the core oligosaccharide begin to fold and are trimmed by mannosidase in the ER-associated quality control compartment system^22^. The correctly folded glycoproteins are subsequently transported to the Golgi apparatus and gradually processed into mature glycoproteins^23,24^. N-glycoproteins and enzymes involved in protein N-glycosylation have been widely identified and found to play vital roles in diverse aspects of development, physiology, and immunity. N-glycosylation was reported to be required to maintain the stability of the chloroplast-localized protein CAH1, which is closely related to photosynthesis in *Arabidopsis thaliana*^25^. Mutation of the oligosaccharyltransferase subunit STT3A affects flagellin peptide Flg22-induced PR1 accumulation and callose deposition, resulting in increased susceptibility to *Pseudomonas syringae* pv. *Tomato* DC3000 in *Arabidopsis*^26^. N-glycosylated PsXEG1 secreted by *Phytophthora sojae* cannot be degraded by the soybean (*Glycine max*) aspartic acid protease GmAP5, allowing it to achieve full virulence in soybean^27^. The asparagine-linked glycosylation 3 (ALG3)-mediated N-glycosylation of Slp1, an effector secreted by *Magnaporthe oryzae*, was essential for the pathogenicity of the rice blast fungus^28^. Blocking ALG3-mediated N-glycosylation caused intense ER stress and growth and virulence defects in the maize (*Zea mays*) anthracnose pathogen *Colletotrichum graminicola*^29^. The apoplastic effector FgLPMO9A from *Fusarium graminearum* suppresses ZmLecRK1-mediated plant immunity by attacking the extracellular glycosylation of this pattern recognition receptor and inducing its degradation through the autophagy pathway^30^. N-glycosylation of envelope proteins from human immunodeficiency virus, Japanese encephalitis virus, and West Nile virus influences membrane fusion, the release of viral particles, and infectivity^31–33^. However, the associations between N-glycosylation and plant viral proteins and the mechanism by which N-glycosylation regulates viral infection are still largely unknown.

Wheat yellow mosaic virus (WYMV) is a member of the genus *Bymovirus* in the family *Potyviridae* and is transmitted by *Polymyxa graminis*, causing severe disease that threatens wheat health and production^34,35^. WYMV was co-infected with CWMV from genus *Furovirus*, family *Virgavirdae*, in some winter wheat regions of China. WYMV has bipartite positive-sense single-stranded RNAs, and the P1 and P2 proteins encoded by RNA2 have been reported to act as VSRs^36,37^. Infected seedlings display yellow-stripe mosaicking on leaves, fewer tillers, and stunting during early spring^38–40^. It is extremely difficult to control this disease via chemical methods because of the thick wall and persistent vitality of the dormant spores of soil-borne *P. graminis*^41,42^. Breeding wheat antiviral varieties is considered a sustainable and effective way to prevent the occurrence of this disease^43^. To date, only a few WYMV resistance-related genes and quantitative trait loci (QTLs) on chromosomes (Chr.) 2 A, 2DL, 3BS, 4B, and 5AL have been identified. Most of these resistance traits are genetically controlled by dominant genes, including *YmNM*, *YmYF*, *Ym2* and *TaRDA21A*^44–49^; only *TaMTB*, located on Chr. 4B, is a susceptibility gene^50^. The disease resistance of crops mediated by resistance genes is strong but easily overcome by evolving viruses, while inactivation of susceptibility genes has the potential for broader recognition and extended durability. Therefore, it is imperative to identify more susceptibility genes for the molecular breeding of wheat virus resistance.

In this work, the oligosaccharyltransferase TaOST1B was identified via a genome-wide association study (GWAS) of 480 accessions and fine mapping using genetic population. Gene function studies showed that TaOST1B is a susceptibility gene that negatively regulates the resistance to WYMV in wheat. Further research revealed that ER-localized TaOST1B could interact with P1 to glycosylate the asparagine residue of P1 at position 116. The N-glycosylation of P1 affects its silencing suppressor activity by affecting its nuclear localization and protein stability. Moreover, we reveal that the protein encoded by natural allele variation of TaOST1B is incapable of interacting with P1 to regulate its N-glycosylation, triggering ER stress and 26S proteasome-mediated degradation. Our study provides a promising target for improving the viral resistance of wheat.

## Results

### Identification of WYMV resistance-related genes by GWAS and QTL mapping

To identify genes regulating resistance to WYMV infection in wheat, we investigated the WYMV infection type in 480 accessions, and GWAS was subsequently performed based on the genotype of the wheat Pan800K array we developed. GWAS results based on five environments revealed that 401 significant markers were mainly distributed on Chr. 2D, 2B, 5A and 7A (Supplementary Data 1). Wherein the resistance locus on Chr. 2D overlapped with the reported WYMV resistance QTL *Qupwym.hau*−2DL (contains dominant gene *TaRD21A*), which was mapped using the recombinant inbred line population of the PI610750 (susceptibility variety) and UC1110 (resistance variety) (UP-RIL)^49^. The second-most numerous significant markers were distributed on Chr. 2B in 480 accessions (this study) and UP-RIL^49^. The significant markers (2B:812514536) were stably detected in four environments, mean and BLUP, and was selected as the candidate WYMV resistance-related locus for further analysis (Supplementary Fig. 1a, Supplementary Data 1). We subsequently calculated the linkage disequilibrium decay distance of Chr. 2B and found that the decay distance was 3.8 Mb when the r^2^ cutoff value was set to 0.3 (Supplementary Fig. 1b). Thus, the candidate interval for this WYMV resistance-related locus was anchored at 808.7–812.8 Mb (about 4.1 Mb) (Supplementary Fig. 1c). Furthermore, the polymorphisms of all genes within this interval were incorporated as markers into the original genotyping array to further analysis, which identified six significant SNPs within the target region and formed a haplotype block spanning ~1.0 Mb between 2B:811541451 and 2B:812515105 on Chr. 2B (Supplementary Fig. 1d). Notably, similar results were obtained with QTL mapping using UP-RIL (Supplementary Fig. 1e). The results showed that two QTLs were identified, of which one located on Chr. 2D is known *Qupwym.hau*−2DL with a LOD score of 16.57 and a phenotypic variance explained (PVE) of 36.87%, and the other QTL located on Chr. 2B (covering the ~1.0 Mb mentioned region) had a LOD score of 6.92 and a PVE of 13.68%, named *Qupwym*−2BL (Supplementary Fig. 1e). Therefore, a WYMV resistance-related locus on Chr. 2B, *Qupwym*−2BL, was identified.

**Fig. 1:**
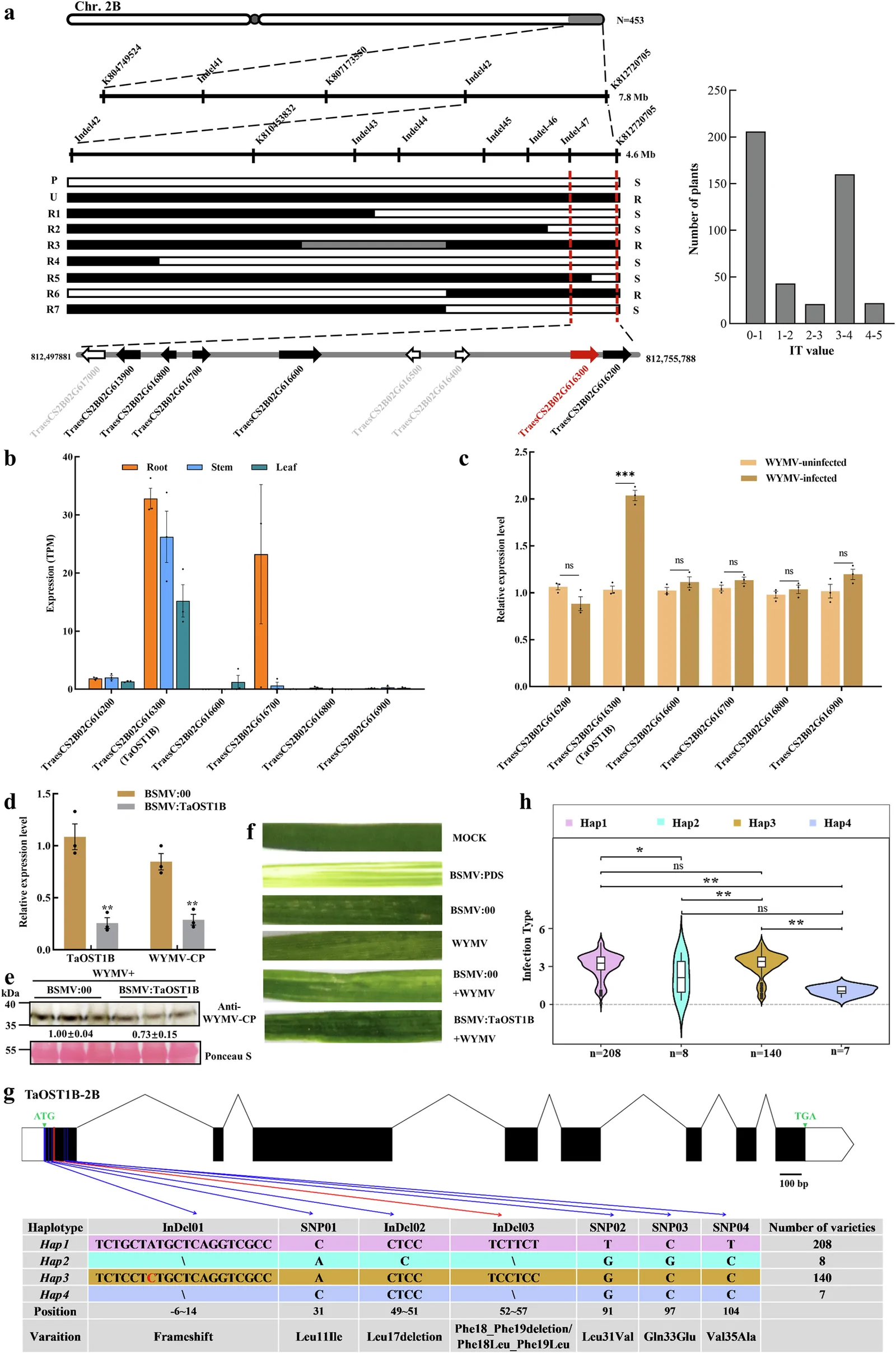
*TaOST1B* was identified as WYMV resistance-related gene on chromosome 2B. **a** Fine mapping of *Qupwym*−2BL using the genotypes and phenotypes of different recombinants from the UP-RHL population. The white and black boxes in the graphical genotype represent genomic segments from PI610750 and UC1110, respectively. The gray boxes represent heterozygous segments. The relevant markers are displayed at the top. The column chart on the right shows the distribution of IT (infection type) values for 453 UP-RHL individuals in WYMV-contained nursery. The white arrows at the bottom represent genes that coding sequences without missense or frameshift variation between PI610750 and UC1110, black represent genes with missense or frameshift variation, and red represent candidate gene that is significantly upregulated after WYMV infection. **b** The expression abundance of 6 genes with nonsynonymous or frameshift variation in the coding region in wheat tissues. The data from the Hexaploid Wheat Gene Expression Database (Chinese Spring Development) in WheatOmics 1.0. The value is the mean ± SD, *n* = 3 biologically independent samples. The graph was generated using GraphPad Prism 9.0. **c** Analysis of the expression levels for 6 genes before and after WYMV infection. *TaCDC* was used as the internal control gene; values are means ± SD (two-sided *t*-test, *n* = 3 biologically independent samples), ns *P* > 0.05, ***P < 0.001. **d** Detection of gene expression and WYMV RNA accumulation in wheat plants co-infected with WYMV and BSMV:TaOST1B or BSMV:00. BSMV:00 and WYMV co-infected plants were used as negative controls. *TaCDC* was used as the internal control gene. Values are means ± SD (two-sided *t*-test, *n* = 3 biologically independent samples). **P < 0.01. **e** Detection of WYMV-CP accumulation in WYMV-infected plants by western blot using a CP-specific antibody. Each treatment included three biological replicates. Signal density of protein bands was quantified using Image J software; the values are the means ± SD. Ponceau staining shows equal protein loadings in each lane. **f** Phenotypes in the fourth leaves of the plants inoculated with phosphate-buffered saline (Mock), BSMV:PDS, BSMV:00, WYMV, BSMV:00 + WYMV, BSMV:TaOST1B + WYMV, respectively. **g** Haplotype analysis of *TaOST1B-2B* in 363 wheat accessions and gene structure display. The black box represents the CDS region, and the blank space represents the UTR region. Different colors represent different haplotypes. **h** Comparison of WYMV infection type between wheat varieties carrying Hap1-Hap4 genotypes. The infection type is derived from the mean value of all replicates. n represents the number of wheat accessions with the corresponding haplotype. For all datasets, two-sided *t*-test was performed, *n* = 208 (Hap1), 8 (Hap2), 140 (Hap3), and 7 (Hap4), respectively. *P < 0.05, **P < 0.01, and ns *P* > 0.05. Boxplots indicate median (box center line), 25th, 75th percentiles (box), and 5th and 95th percentiles (whiskers). Source data are provided as a Source Data file.

To further target the candidate genes in this locus, we utilized a residual heterozygous line (RHL) population (*n* = 453) derived from the UP-RILs, in which individuals were heterozygous at the *Qupwym*−2BL locus yet homozygous for the UC1110 genotype at *Qupwym.hau*−2DL, for fine mapping. The results showed that the *Qupwym*−2BL was further narrowed to 222.8 kb between markers Indel47 and K812720705 (Fig. 1a). This interval encompasses nine genes, of which six genes exhibit nonsynonymous or frameshift mutations within coding regions between parental lines. Nevertheless, only one gene, encode the oligosaccharyltransferase (*TraesCS2B02G616300*, *TaOST1B*), was highly expressed in roots, stems, and leaves in the hexaploid wheat expression database and significantly upregulated post-viral infection in the susceptible line PI610750 (Fig. 1b, c). Moreover, the N-terminal region of TaOST1B has two fragment deletions and one amino acid substitution in the parental lines (Supplementary Fig. 1f).

Consequently, TaOST1B was chosen to knock down its expression levels via barley stripe mosaic virus-induced gene silencing (BSMV-VIGS) in wheat. Wheat seedlings with two leaves were inoculated with BSMV:TaOST1B. BSMV:TaPDS served as a reference control, and BSMV:00 served as a negative control. The qRT-PCR results revealed that the expression level of TaOST1B in the BSMV:TaOST1B-inoculated plants was approximately 75% lower than that in the BSMV:00-inoculated plants at 10 days post inoculation (Fig. 1d). We then inoculated the silenced plants with WYMV. At 14 days post inoculation (dpi), the qRT-PCR results revealed that the accumulation level of WYMV RNA in the BSMV:TaOST1B+WYMV-inoculated plants was significantly lower than that in the BSMV:00 + WYMV-inoculated plants (Fig. 1d). Western blot analysis revealed a similar trend (Fig. 1e). Moreover, milder mosaic symptom was observed on the leaves of BSMV:TaOST1B+WYMV-inoculated plants than BSMV:00 + WYMV-inoculated plants (Fig. 1f). This result showed that TaOST1B could be the candidate gene of *Qupwym*−2BL, which may negatively regulate resistance to WYMV in wheat.

To further evaluate whether natural variation of TaOST1B is associated with resistance to WYMV among wheat varieties, we investigated the TaOST1B sequence of 363 accessions and found that four haplotypes, named Hap1-Hap4, were identified for TaOST1B based on four nonsynonymous SNPs and three InDels (Supplementary Data 2). Compared to Hap1, Hap2 exhibits amino acid deletions at positions 1–6 and 17–19, along with substitutions at positions 11, 31, 33, and 35. Hap3 lacks residues 1–6 due to the mutation of the start codon ATG to CTG, with additional substitutions at positions 11, 18, 19, 31, and 35. Hap4 shows deletions at positions 1–6 and 18–19, and substitutions at positions 31 and 35 (Fig. 1g). The shared variation of Hap2/Hap4 have 6-bp (2-amino acids at positions 18 and 19) deletion compared with Hap1/Hap3, and Hap2 and Hap4 presented significantly milder WYMV susceptibility than Hap1 and Hap3 (Fig. 1g, h). Therefore, we hypothesize that the 2-amino acid deletion in TaOST1B constitutes a critical variation attenuating WYMV infection in wheat.

### *TaOST1B*^*Hap1*^ is a positive regulator of WYMV infection in wheat

**Fig. 2:**
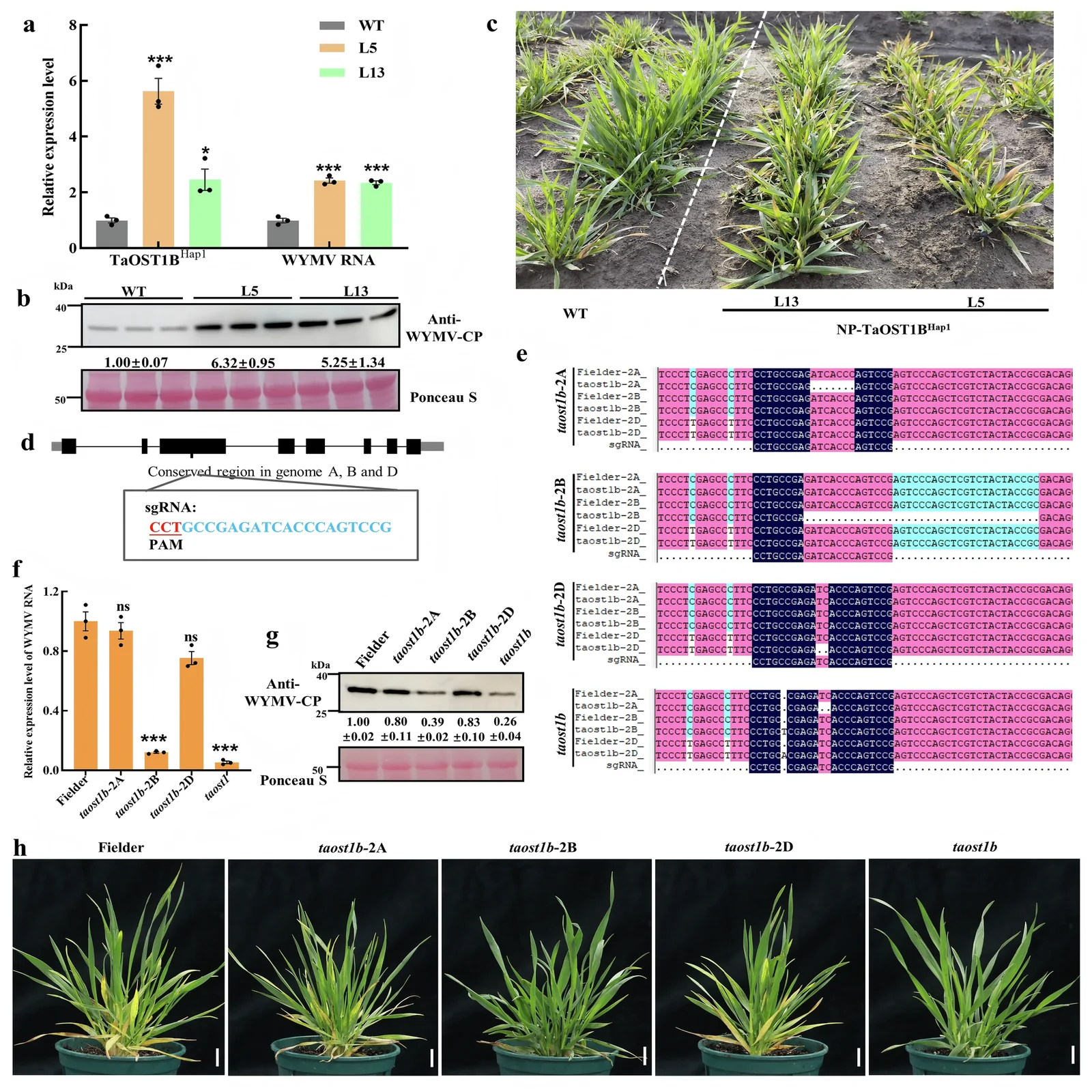
*TaOST1B* positively regulates WYMV infection in wheat. **a** The relative expression levels of *TaOST1B* (left) and accumulation of WYMV RNA (Right) in the WYMV-infected *TaOST1B* transgenic lines and wild-type (WT) plants were determined by qRT-PCR. *TaCDC* was used as the internal control gene. Values are means ± SD (two-sided *t*-test, *n* = 3 biologically independent samples), *P < 0.05, ***P < 0.001. **b** Detection of WYMV-CP accumulation in WT plants or two NP-TaOST1B^Hap1^ lines by western blot using a CP-specific antibody. Each group included three biological replicates. Signal density of protein bands was quantified using Image J software; the values are the means ± SD. Ponceau staining shows sample loadings. **c** Assessment of NP-TaOST1B^Hap1^ lines for disease resistance in the WYMV-contaminated nursery at Yangzhou, Jiangsu Province, in 2024. WT represents Fielder plants. **d** Schematic showing the genetic structure of *TaOST1B*. Gray boxes represent untranslated regions, black boxes represent coding regions, and lines represent introns. The sgRNA was designed to target all three homoeoalleles in wheat by CRISPR-Cas9. **e** Three homoeoalleles sequence of *TaOST1B* in Fielder and knockout mutant. The sequence of *TaOST1B* on chromosome 2A in *taost1b*−2A mutant contains a 7-bp deletion, *taost1b*−2B mutant contains a 37-bp deletion on chromosome 2B, *taost1b*−2D mutant contains a 2-bp deletion on chromosome 2D, respectively. The sequence of *TaOST1B* in *taost1b* mutant contains a 2-bp deletion on chromosome 2A and a 1-bp insertion on chromosome 2B/2D. **f**, **g** Detection of WYMV RNA and CP accumulation in the assayed wheat plants by qRT-PCR (**f**) and western blot assays (**g**), respectively. *TaCDC* was used as the internal control gene; values are means ± SD (two-sided *t*-test, *n* = 3 biologically independent samples), ***P < 0.001 and ns *P* > 0.05. Signal density of protein bands was quantified using Image J software; the values are the means ± SD. Ponceau staining shows sample loadings; three times the experiment was repeated with similar results. **h** Assessment of knockout mutant T2 lines for disease resistance in the WYMV-containted soil. Fielder plants as the control. Bars indicate 2 cm. Source data are provided as a Source Data file.

To further validate the regulatory function of *TaOST1B* in WYMV infection, we generated several transgenic wheat lines expressing native promoter-driven *TaOST1B*^*Hap1*^(NP-TaOST1B^Hap1^) or *TaOST1B*^*Hap4*^(NP-TaOST1B^Hap4^) in the cv. ‘Fielder’. The transgenic lines and wild-type plants were inoculated with WYMV. The expression level of *TaOST1B* via qRT-PCR revealed that line 5 (L5) and line 13 (L13) of NP-TaOST1B^Hap1^ and line 7 (L7) and line 10 (L10) of NP-TaOST1B^Hap4^ presented greater mRNA accumulation than did Fielder at 14 dpi (Fig. 2a, Supplementary Fig. 2a). Moreover, the results of qRT-PCR and western blot analysis revealed that the accumulation levels of WYMV RNA and coat protein (CP) were significantly greater in L5 and L13 of NP-TaOST1B^Hap1^than in the wild-type plants (Fig. 2a, b). We also observed that the NP-TaOST1B^Hap1^-L5 and -L13 plants presented more severe mosaic symptoms than did the wild-type plants infected with WYMV (Fig. 2c). While the accumulation levels of WYMV RNA and CP in L7 and L10 of NP-TaOST1B^Hap4^ showed no difference compared with that in the wild-type plants (Supplementary Fig. 2a, b), and NP-TaOST1B^Hap4^ -L7 and -L10 plants presented mosaic symptoms similar to the wild-type plants (Supplementary Fig. 2c). These results indicated that*TaOST1B*^*Hap1*^ acts as a susceptibility gene to promote WYMV infection, *TaOST1B*^*Hap4*^ is the nonfunctional allele for WYMV infection. Furthermore, we employed CRISPR/Cas9 technology to knockout*TaOST1B*^*Hap1*^in Fielder, employing an sgRNA targeting a conserved region common to the three copies of *TaOST1B* on Chr. 2 A, 2B, and 2D (Fig. 2d). Through Hitom sequencing, we successfully identified *TaOST1B* knockout lines in which A copy, B copy, and D copy alone were edited, respectively (*taost1b*−2A, *taost1b*−2B, and *taost1b*−2D), and a knockout line where all three copies were edited (*taost1b*) (Fig. 2e, Supplementary Data 3). These mutants were planted in nursery with WYMV, the single mutant *taost1b*−2B and triple mutant *taost1b* exhibited markedly enhanced WYMV resistance phenotypes compared with Fielder plants, accompanied by a marked reduction in viral RNA and protein levels compared with those of Fielder (Fig. 2f–h). While single mutant *taost1b*−2A and *taost1b*−2D showed distinct, similar mosaic symptoms to Fielder plants, and viral RNA and protein levels showed no differences (Fig. 2f–h, Supplementary Fig. 2d, e). Moreover, transcriptional profiling of three copies before and after WYMV infection demonstrated that the expression levels of only the B copy were markedly upregulated post-infection, while expression levels of the A copy and D copy showed no significant changes (Supplementary Fig. 2f). These findings suggest that the *TaOST1B*^*Hap1*^−2B could play a dominant role in mediating WYMV infection.

### TaOST1B^Hap1^ interact with WYMV P1 to regulate N-glycosylation of P1 at 116

**Fig. 3:**
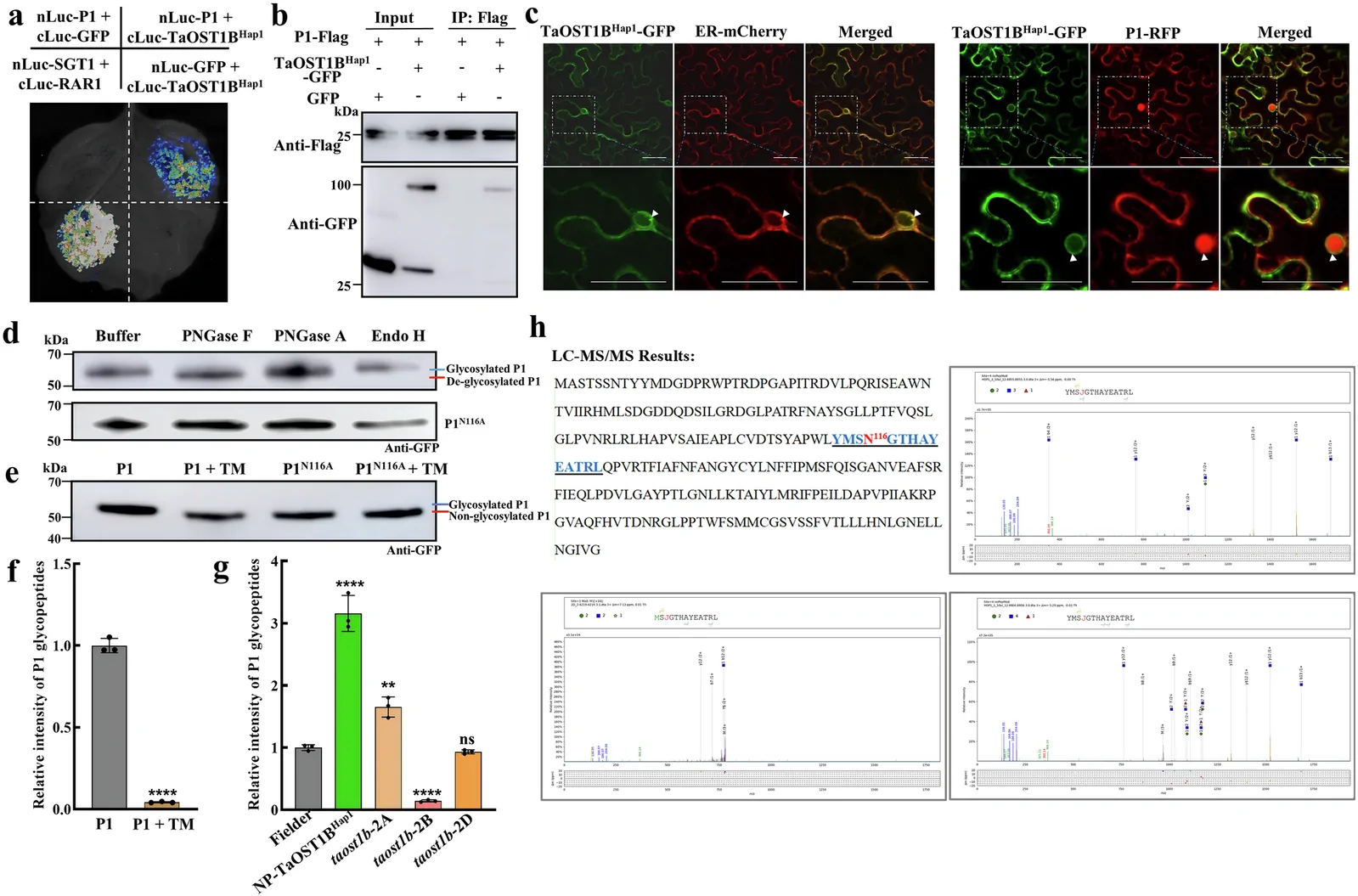
TaOST1B^Hap1^ interact with WYMV P1 to regulate the N-glycosylation of P1 at 116. **a** Interaction of TaOST1B^Hap1^ with P1 was detected by LCI assay in *N. benthamiana* leaves. The leaf areas infiltrated with *Agrobacterium* expressing nLuc-SGT1+cLuc-RAR1 were used as a positive control, nLuc-P1+cLuc-GFP and nLuc-GFP+cLuc-TaOST1B^Hap1^ were used as negative controls, respectively. Luciferase activity was captured using a low-light cooled CCD imaging apparatus at 2 days post-infiltration (dpi). **b** Co-immunoprecipitation (Co-IP) analysis of the interaction between TaOST1B^Hap1^ and P1. P1-Flag with GFP were the negative control groups, and P1-Flag with TaOST1B^Hap1^-GFP were tested. Samples were collected at 2 dpi, following which Co-IP with Flag beads was performed. Three times the experiment was repeated with similar results. **c** Subcellular localization of TaOST1B^Hap1^-GFP and colocalization of TaOST1B^Hap1^-GFP with P1-RFP in the leaf cells of *N. benthamiana* by confocal microscopy at 48 hpi. Localization of ER-mCherry was used as an ER marker. The corresponding region in the white box was enlarged below it. The arrow indicates the ER location. Bars indicate 50 μm. Three times the experiment was repeated with similar results. **d**, **e** P1 and P1^N116A^ size migration analysis of protein extracts from *N. benthamiana* cells expressing P1-GFP or P1^N116A^-GFP treated with EndoH, PNGase A, PNGase F glycosidases or a buffer control (**d**), and treated with or without TM (**e**). Western blotting was performed using GFP antibody. Three times the experiment was repeated with similar results. **f** Analysis of N-glycosylation abundance of P1. The intensity of P1 glycopeptides was examined by LC-MS/MS for TM-treated and -untreated P1. The plot is generated by GraphPad Prism software. The column height represents the relative intensities of the P1 glycopeptides from different samples on the site-specific level. Values are means ± SD (two-sided *t-*test, *n* = 3 technically independent replicates), ****P < 0.0001. **g** Analysis of N-glycosylation abundance of P1 in Fielder, NP-TaOST1B^Hap1^, *taost1b*−2A, *taost1b*−2B, *taost1b*−2D wheat plants. The intensity of P1 glycopeptides was examined by LC-MS/MS. The column height represents the relative intensities of the P1 glycopeptides from different samples on the site-specific level. Values are means ± SD (two-sided *t*-test, *n* = 3 technically independent replicates), ****P < 0.0001, **P < 0.01, ns *P* > 0.05. **h** Identification of N-glycosylated site and representative spectra of the intact N-glycopeptide from P1 by LC-MS/MS. The glycopeptide is shown as an underscore (upper left), and the amino acid highlighted in red is the N-glycosylated site. The remaining three panels present representative MS/MS spectra for qualitative identification of the detected glycoforms, revealing structural details of the peptide backbone and glycan composition. The red letter “J” indicates the glycosylated asparagine residue. Source data are provided as a Source Data file.

To understand the molecular mechanism by which TaOST1B^Hap1^ promotes WYMV infection, we initially predicted the potential N-glycosylation sites of WYMV-encoded proteins using a website (NetNGlyc 1.0 server, Supplementary Data 4), and a split luciferase complementation imaging (LCI) assay was subsequently performed in *Nicotiana benthamiana* leaves by coexpressing TaOST1B^Hap1^ and 10 WYMV-encoded proteins. The results showed that only P1 exhibited a discernible interaction with TaOST1B^Hap1^ (Fig. 3a, Supplementary Fig. 3a). The yeast two-hybride (Y2H) assay showed the same results (Supplementary Fig. 3b). Moreover, LCI assays demonstrated that neither TaOST1B-2A nor TaOST1B-2D interacts with P1, only TaOST1B^Hap1^−2B could interacts with P1 (Supplementary Fig. 3c). This interaction was further validated by a co-immunoprecipitation (Co-IP) assay (Fig. 3b). To determine the subcellular localization, TaOST1B, TaOST1B^Hap1^-GFP or TaOST1B^Hap4^-GFP was coexpressed with ER-mCherry (an endoplasmic reticulum marker, AtWAK2) in *N. benthamiana* leaves. The results showed that the fluorescent signals of TaOST1B^Hap1^ or TaOST1B^Hap4^-GFP almost completely overlapped with those of the ER marker (Fig. 3c, Supplementary Fig. 3d). TaOST1B^Hap1^-GFP and P1-RFP were subsequently coexpressed in *N. benthamiana* leaves. P1 and TaOST1B^Hap1^ exhibited colocalization in the ER (Fig. 3c). These results indicate that TaOST1B^Hap1^ may regulate the N-glycosylation of P1 in the ER.

To study whether P1 could be N-glycosylated in plants, P1-GFP and P1^N116A^-GFP (with a mutation in the predicted glycosylation site for P1) expressed in *N. benthamiana* were immunoprecipitated and treated with various glycosidases, including peptide-N-glycosidase F (PNGase F), peptide-N-glycosidase A (PNGase A), and endoglycosidase H (Endo H). Following treatment with either PNGase F or PNGase A, P1-GFP migrated faster than did Endo H and buffer (Fig. 3d). Compared with buffer-treated protein, P1^N116A^-GFP showed no shift upon treatment with any of the three enzymes (Fig. 3d). Subsequently, the immunoprecipitated P1 from WYMV-infected leaves was treated with PNGase F, and the mobility shift of P1 before and after digestion was analyzed using a P1-specific antibody. The results showed that PNGase F-treated P1 exhibited faster migration compared to the untreated controls (Supplementary Fig. 3e). Moreover, the migration rate of tunicamycin (TM, N-glycosylation inhibitor)-treated P1 was slightly changed compared with those of untreated P1 and P1^N116A^ (Fig. 3e). The nanoscale liquid chromatography-tandem mass spectrometry (LC-MS/MS) quantitative analysis showed that the relative intensity of P1 glycopeptides from TM-treated sample was significantly lower than that of untreated P1 (Fig. 3f, Supplementary Fig. 4a, Supplementary Data 5a), and the relative proportion of glycosylated P1 peptides also significantly decreased under TM treatment (Supplementary Fig. 4b, Supplementary Data 6a). Furthermore, to investigate whether TaOST1B^Hap1^ regulates the N-glycosylation of P1 in wheat, N-glycosylation intensity of P1 in Fielder, NP-TaOST1B^Hap1^, *taost1b−*2A, *taost1b−*2B, and *taost1b−*2D lines with WYMV infection were detected by LC-MS/MS. The results revealed that the relative intensity of P1 glycopeptides was significantly greater in NP-TaOST1B^Hap1^ plants than in Fielder plants, but significantly lower in *taost1b−*2B plants than in Fielder, while there was no decrease in *taost1b*-2A, *taost1b*-2D than in Fielder (Fig. 3g, Supplementary Fig. 4c, Supplementary Data 5b). The relative proportion of glycosylated P1 peptides also exhibited a similar trend in these wheat lines (Supplementary Fig. 4d, Supplementary Data 6b). The N-glycosylated site and representative higher-energy collisional dissociation fragmentation spectra of the N-glycopeptides of P1 in wheat are shown in Fig. 3h. These findings indicate that TaOST1B^Hap1^-2B is primarily required for glycosylation of P1 asparagine at 116.

### N-glycosylation of P1 facilitates entry into the nucleus to maintain VSR activity, promoting WYMV infection

**Fig. 4:**
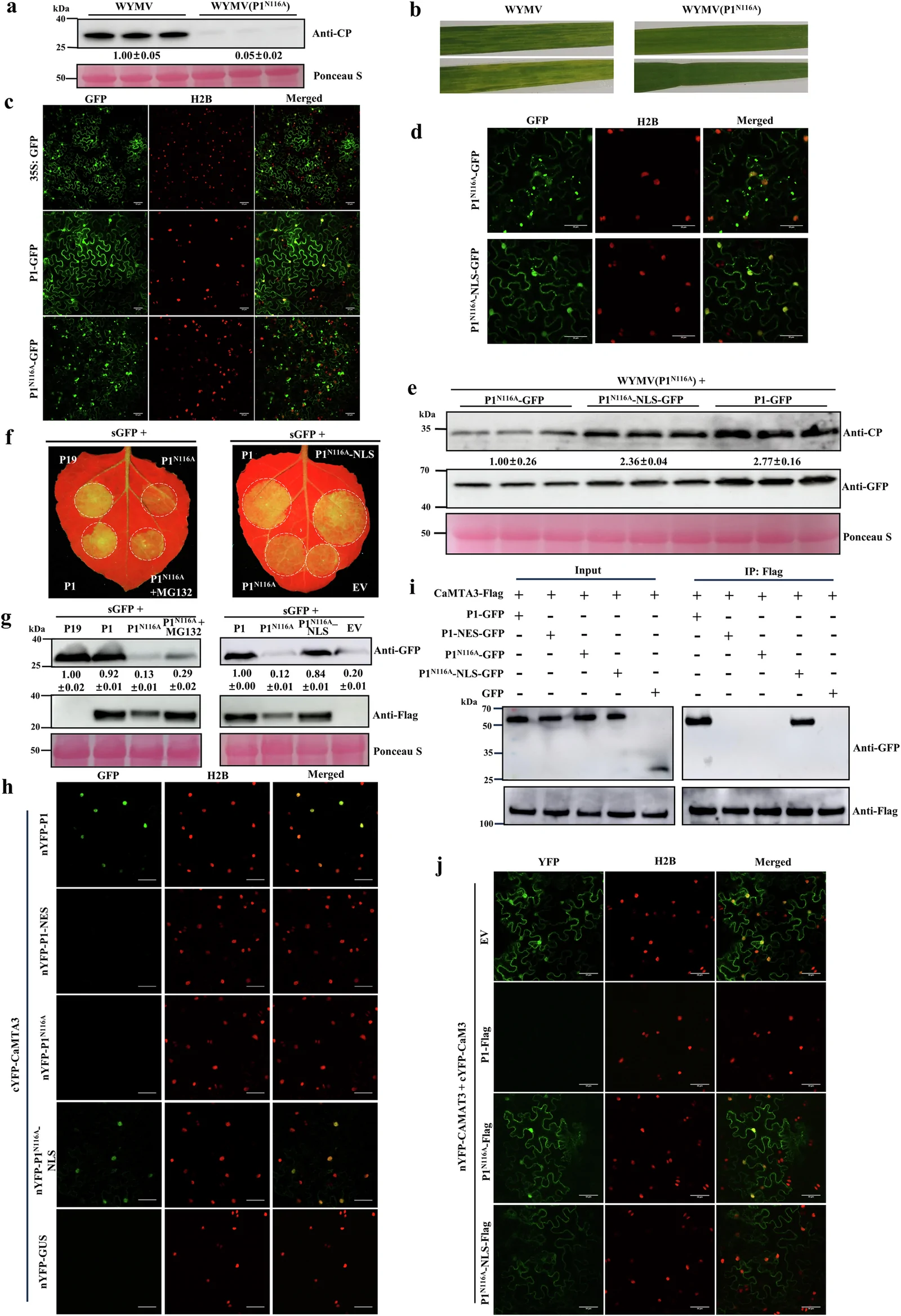
N-glycosylation of P1 assists it in entering the nucleus to maintain VSR activity. **a** The accumulation of WYMV-CP in the WYMV- or WYMV(P1^N116A^)-inoculated plants was determined by western blot assays. The CP-specific antibody was used. Each treatment included three biological replicates. Signal density of protein bands was quantified using Image J software; the values are the means ± SD. Ponceau staining shows sample loadings. **b** WYMV symptoms in systemic leaves of the WYMV- or WYMV(P1^N116A^)-inoculated plants at 40 dpi. **c** The subcellular localizations of P1-GFP and P1^N116A^-GFP in the leaf cells of H2B-RFP transgenic *N. benthamiana* by confocal microscopy at 48 hpi. Localization of 35S:GFP was used as control. The red fluorescence represents the nuclear signals of all cells and was used as a reference for the GFP signal. Bars indicate 50 μm. Three times the experiment was repeated with similar results. **d** The subcellular localizations of P1^N116A^-GFP and P1^N116A^-NLS-GFP in the leaf cells of H2B-RFP transgenic *N. benthamiana* by confocal microscopy at 48 hpi. Bars indicate 50 μm. Three times the experiment was repeated with similar results. **e** The accumulation of WYMV-CP in the WYMV(P1^N116A^)-inoculated plants expressing P1^N116A^-GFP or P1^N116A^-NLS-GFP was determined by western blot assays using CP-specific antibody. The accumulation of P1^N116A^ or P1^N116A^-NLS proteins was detected using GFP antibody. Each treatment included three biological replicates. Signal density of protein bands was quantified using Image J software; the values are the means ± SD. Ponceau staining shows sample loadings. **f** Suppression assay of local RNA silencing of P1, P1^N116A^ and P1^N116A^-NLS in *N. benthamiana* leaves. sGFP with P1-Flag, P1^N116A^-Flag or P1^N116A^-NLS-Flag were infiltrated into *N. benthamiana* leaves, respectively. MG132 was added to inhibit the possible degradation of P1^N116A^. EV was used as the negative control, and P19 was used as the positive control. The GFP fluorescence under long-wavelength UV light was photographed at 3 dpi. **g** Western blot analysis in the indicated samples in (**f**). Anti-GFP antibody was used to detect the accumulation of GFP, anti-Flag antibody was used to detect P1-Flag, P1^N116A^-Flag or P1^N116A^-NLS-Flag fusion protein. Each lane represents a sample from an individual experiment; three times the experiment was repeated with similar results. Signal density of protein bands was quantified using Image J software; the values are the means ± SD. Ponceau staining shows sample loadings. **h** Interactions of P1, P1-NES, P1^N116A^, and P1^N116A^-NLS with CaMTA3 in H2B-RFP transgenic *N. benthamiana* were examined using BiFC assay. GUS as the negative control. Bars indicate 50 μm. Three times the experiment was repeated with similar results. **i** Co-IP analysis of the interactions of P1, P1-NES, P1^N116A^, and P1^N116A^-NLS with CaMTA3. CaMTA3-Flag with GFP were the negative control groups, and P1, P1-NES, P1^N116A^, and P1^N116A^-NLS with CaMTA3-Flag were tested. Samples were collected at 2 dpi, following which Co-IP with Flag beads was performed. Three times the experiment was repeated with similar results. **j** Detection of NbCaM3 and NbCaMTA3 interaction using bimolecular fluorescence complementation (BiFC) assay through expressing P1, P1^N116A^ or P1^N116A^-NLS in *N. benthamiana*. Photos were taken 48 h post-infiltration. Bars indicate 50 μm. Three times the experiment was repeated with similar results. Source data are provided as a Source Data file.

To investigate the role of N-glycosylation of P1 at 116 in WYMV infection, we generated a mutant WYMV infectious clone, WYMV (P1^N116A^), by substituting the asparagine residue at position 116 of P1 with a nonglycosylated alanine residue (Supplementary Fig. 5a). The WT and mutant infectious clones were inoculated individually into *N. benthamiana* leaves through agroinfiltration. Western blot analysis revealed that the accumulation level of WYMV-CP in WYMV(P1^N116A^)-inoculated leaves was markedly lower than that in WYMV-inoculated leaves at 14 dpi (Fig. 4a). Wheat seedlings were subsequently inoculated with WYMV or WYMV(P1^N116A^). The qRT-PCR results revealed that the expression level of WYMV-CP in the systemic leaves of WYMV(P1^N116A^)-inoculated plants was significantly lower than that in the systemic leaves of WYMV-inoculated plants at 14 dpi (Supplementary Fig. 5b). At 40 dpi, the young developing leaves of wheat plants inoculated with WYMV(P1^N116A^) presented milder mosaic symptoms than those inoculated with WYMV (Fig. 4b). These findings suggest that N-glycosylation of P1 can promote WYMV infection in plants.

To explore the effect of P1 N-glycosylation on its function, we first examined the subcellular localization of P1 and P1^N116A^. The results revealed that P1 was distributed in both the nucleus and the cytoplasm (Fig. 4c), whereas P1^N116A^ presented weaker fluorescence signals in the cytoplasm, and the fluorescence signals in the nucleus almost disappeared, while obvious dot-like aggregates appeared in the cells (Fig. 4c). To further characterize their localization, P1-GFP or P1^N116A^-GFP and ER-mCherry were coexpressed with ER-mCherry in WYMV-infected and -uninfected *N. benthamiana* leaves. The results showed that P1^N116A^-GFP was mainly localized to ER-associated aggregates, whereas P1-GFP localized to both the nucleus and the ER membrane (Supplementary Fig. 5c). Western blot analysis revealed that P1 accumulation level was greater than P1^N116A^, especially in the nucleus (Supplementary Fig. 5d). In addition, we performed nuclear and cytoplasmic fractionation in NP-TaOST1B^Hap1^ lines, *taost1b-*2A, *taost1b-*2B, and *taost1b-*2D mutant lines, and wild-type wheat plants under WYMV infection. The accumulation levels of P1 in nuclear and cytoplasmic fractions were quantified using P1-specific antibodies. The results showed that total P1 accumulation in NP-TaOST1B^Hap1^ lines was higher than in both wild-type and *taost1b-*2B lines, and P1 accumulation in the nucleus of transgenic lines was elevated compared with wild-type plants, while P1 was barely detectable in the nuclear fraction of *taost1b-*2B lines, while there are no differences in the nuclear accumulation levels of P1 between the wild-type and *taost1b-*2A or *taost1b*-2D lines (Supplementary Fig. 6a). These findings suggest that N-glycosylation may maintain P1 protein stability and is essential for its nuclear localization. To further determine whether the loss of WYMV(P1^N116A^) pathogenicity is related to the reduced nuclear localization signal (NLS) of P1^N116A^, an NLS sequence was artificially added to the C-terminus of P1^N116A^ to generate the P1^N116A^-NLS-GFP fusion protein. P1^N116A^-NLS-GFP or P1^N116A^-GFP was expressed in *N. benthamiana* leaves inoculated with WYMV (P1^N116A^), and P1-GFP was expressed as the control. Laser confocal microscopy revealed that the fluorescence signal in the nucleus of the cells expressing P1^N116A^-NLS-GFP was stronger than that of the cells expressing P1^N116A^-GFP at 4 dpi (Fig. 4d). Western blot analysis revealed that the NLS enhanced the stability of P1^N116A^ and that the accumulation level of WYMV-CP in leaves expressing P1^N116A^-NLS-GFP was greater than that in leaves expressing P1^N116A^-GFP, but slightly weaker than that expressing P1-GFP (Supplementary Fig. 6b, Fig. 4e), which indicates that the nuclear localization of P1^N116A^ has the potential to partially restore the pathogenicity of WYMV (P1^N116A^).

The P1 reportedly acts as a VSR by interfering with CaM3-CaMTA3 interaction-mediated antiviral RNAi defense in plants^36^. To investigate the effect of N-glycosylation of P1 on VSR activity, P1-Flag, P1^N116A^-Flag or P1^N116A^-NLS-Flag was coexpressed with soluble GFP (sGFP) in *N. benthamiana* leaves. The empty vector (EV) and P19 were included in the assay as negative and positive controls, respectively. Leaf cells presented obvious green fluorescence at P1, P1^N116A^-NLS or P19 under long-wavelength ultraviolet (UV) light at 72 h post-infiltration (hpi), whereas leaf cells presented extremely weak green fluorescence at P1^N116A^, which was slightly enhanced by the addition of the proteasome inhibitor MG132 (Fig. 4f). The GFP accumulations were examined by western blotting (Fig. 4g). Further interaction analysis found that P1 could bind to CaMTA3 in the nucleus, while nonglycosylated P1^N116A^ failed to interact with CaMTA3. Upon fusion with an NLS, P1^N116A^-NLS regained the ability to bind CaMTA3 in the nucleus. In contrast, appending a nuclear export signal to P1 (P1-NES) abolished its interaction with CaMTA3 (Supplementary Fig. 6c, Fig. 4h, i). These results demonstrate that nuclear localization of P1 is required for its interaction with CaMTA3. Furthermore, nuclear-localized P1 and P1^N116A^-NLS disrupted the interaction between CaM3 and CaMTA3 and inhibited the expression of related genes in the Ca^2+^-activated RNAi pathway, whereas nonglycosylated P1^N116A^ could not block CaM3-CaMTA3 interaction, and the expression of *CaM3* and *RDR6* was significantly lower in samples expressing P1 or P1^N116A^-NLS than P1^N116A^ (Fig. 4j, Supplementary Fig. 6d). These results imply that the nuclear import of P1, even though nonglycosylation, could block the CaMTA3-CaM3 interaction maintaining VSR activity and promote WYMV infection.

### N-glycosylated P1 interacts with the TaIMP-α2 to be transported to the nucleus

**Fig. 5:**
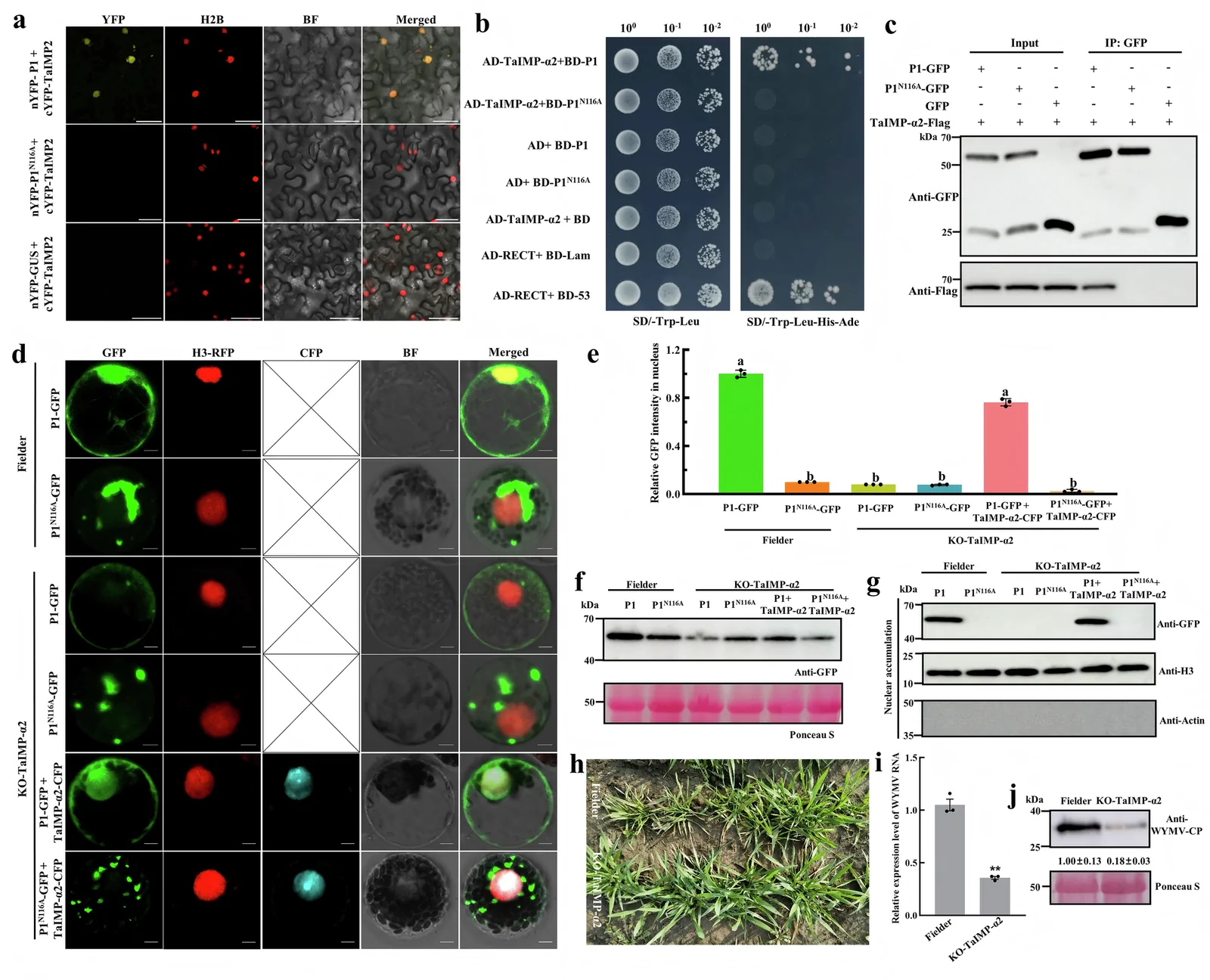
TaIMP-α2 can interact with P1 to transport it to the nucleus. **a** Interactions of P1 and P1^N116A^ with TaIMP-α2 proteins in *N. benthamiana* were examined using the BiFC assay. GUS as the negative control. Bars indicate 50 μm. **b** a Yeast two-hybrid assay for the interaction between interactions of P1 and P1^N116A^ with TaIMP-α2. Yeast co-transformed with AD-RECT + BD-53 or AD-RECT + BD-Lam served as a positive and negative control, respectively. **c** Co-IP analysis of the interactions of P1 and P1^N116A^ with TaIMP-α2. TaIMP-α2-Flag with GFP were the negative control groups, and P1-GFP and P1^N116A^-GFP with TaIMP-α2-Flag were tested. Samples were collected at 2 dpi, following which Co-IP with GFP beads was performed. Three times the experiment was repeated with similar results. **d**, **e** Subcellular localization of P1-GFP and P1^N116A^-GFP after being expressed in wheat protoplasts along with H3-RFP, which served as a nuclear marker. Bars indicate 5 μm (**d**). The relative intensities of nucleus-located GFP signals resulting from the six groups were statistically compared (**e**). Values were the means ± SE of three repeats, which were analyzed using one-way ANOVA tests; different letters represent significant differences, *P* < 0.05. **f** Verifying the expression of P1-GFP and P1^N116A^-GFP in transfected wheat protoplasts by western blotting with an anti-GFP antibody. Ponceau staining shows sample loadings. Three times the experiment was repeated with similar results. **g** Nuclear accumulation levels of the six groups were evaluated by western blotting. The purity of the nuclear protein samples was verified by the positive detection of Histone 3 (H3) but the absence of Actin. Three times the experiment was repeated with similar results. **h** Assessment of KO-TaIMP-α2 wheat plants for disease resistance in a WYMV-contaminated nursery at Yangzhou, Jiangsu Province, in 2024. **i**, **j** Detection of WYMV RNA and CP accumulation in the assayed wheat plants by qRT-PCR (**i**) and western blot (**j**) assays, respectively. *TaCDC* was used as the internal control gene; values are means ± SD (two-sided *t*-test, *n* = 3 biologically independent samples), **P < 0.01. Signal density of protein bands were quantified using Image J software; the values are the means ± SD. Ponceau staining shows sample loadings. Three times the experiment was repeated with similar results. Source data are provided as a Source Data file.

To explore the host factors that help P1 enter the nucleus, we identified host proteins that may interact with P1 in wheat via IP-MS. An importin subunit alpha protein, TaIMP-α2, was screened and identified. We then performed a biomolecular fluorescence complementation (BiFC), Y2H and CoIP assays to test the interaction of TaIMP-α2 with P1 or P1^N116A^. The results showed that TaIMP-α2 could interact with P1 in the nucleus (Fig. 5a–c). Moreover, Co-IP results showed that P1^N116A^-NLS restored its interaction with TaIMP-α2 (Supplementary Fig. 7a). Coincidentally, TaIMP-α2 could be required for the nuclear localization of VSR 17K encoded by the barley yellow dwarf virus (BYDV)^51^. We used the TaIMP-α2 mutant α1^AABBdd^/α2^aabbdd^ (KO-TaIMP-α2) from that study, and coexpressed Histone 3 (H3)-RFP, a nuclear marker, and P1-GFP or P1^N116A^-GFP in protoplasts of KO-TaIMP-α2 and Fielder (WT). Confocal microscopy revealed that P1-GFP was more strongly accumulated in the nucleus, and almost no obvious nuclear GFP signal was observed for P1^N116A-^GFP in Fielder; P1-GFP and P1^N116A-^GFP showed no visible nuclear localization signals in KO-TaIMP-α2 (Fig. 5d, e). Subsequently, we resupplemented TaIMP-α2-CFP in protoplasts of KO-TaIMP-α2 and found that the nuclear GFP fluorescence signal from P1-GFP was restored, whereas P1^N116A^-GFP still showed no obvious nuclear localization (Fig. 5d, e). The total and nuclear accumulation levels of P1-GFP and P1^N116A^-GFP in protoplasts were also determined via western blotting (Fig. 5f, g). In addition, we immunoprecipitated P1 from the nuclear protein fractions of wheat protoplasts expressing P1-GFP, which was treated with PNGase F, and the mobility shift was analyzed using a GFP antibody. The result showed that PNGase F-treated P1 exhibited faster migration compared to the untreated controls (Supplementary Fig. 7b). These results suggested that TaIMP-α2 was required for P1 transport to the nucleus, and the N-glycosylation form of P1 is present in the nucleus. In addition, we found that, compared with Fielder plants, KO-TaIMP-α2 plants presented milder mosaic symptoms in the WYMV-contaminated nursery (Fig. 5h), and lower viral RNA and protein levels (Fig. 5i, j).

### P1^N116A^ triggers ER stress and accelerates its degradation via the 26S proteasome

**Fig. 6:**
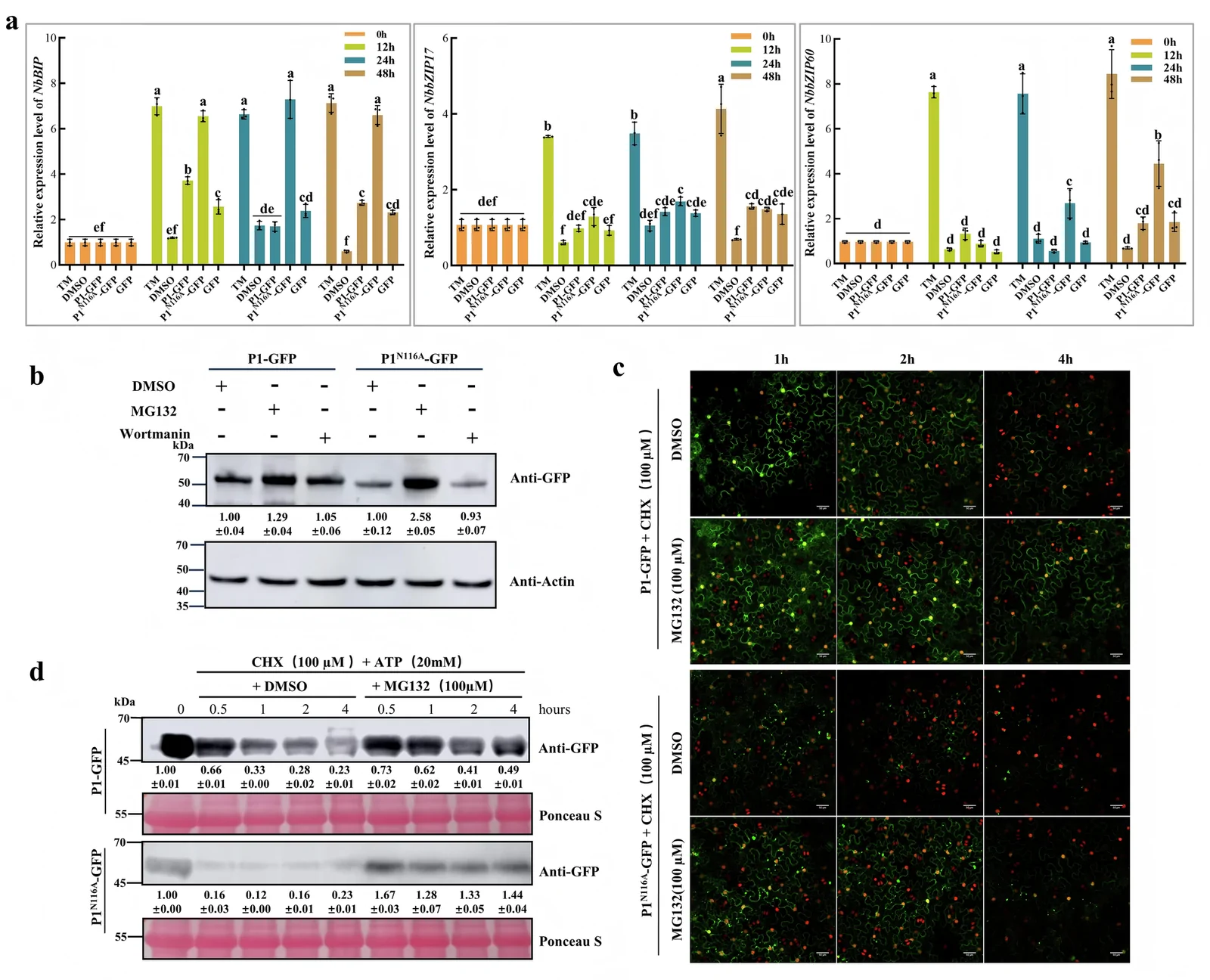
P1^N116A^ triggers ER stress and accelerates its degradation via the 26S proteasome. **a** The expression analysis of *NbBIP*, *NbbZIP17*, and *NbbZIP60*, endoplasmic reticulum (ER)-stress-related representative genes by qRT-PCR in samples expressing P1-GFP and P1^N116A^-GFP for time cross. 6 µM ER stress inducer Tunicamycin (TM) treatments were used as a positive control, and DMSO and GFP infiltration were used as negative control. *NbActin* was used as the internal control gene. Values are means ± SD (one-way ANOVA tests, *n* = 3 biologically independent samples), different letters represent significant differences, *P* < 0.05. **b** Effect of MG132 and Wortmannin on the stability of P1-GFP and P1^N116A^-GFP proteins expressed in wheat protoplasts by in vivo assay. The proteins were extracted at 4 h when protoplasts were treated with 50 µM MG132, Wortmannin or an equal volume of DMSO (control). Each lane represents a sample from an individual experiment; three times the experiment was repeated with similar results. Signal density of protein bands was quantified using Image J software; the values are the means ± SD. Anti-Actin was used as a protein loading control. **c** Effect of MG132 on the stability of P1 and P1^N116A^ protein by in vivo assay. 100 µM CHX, in the presence of 100 µM MG132 or an equal volume of DMSO (control) were infiltrated into H2B-RFP transgenic *N. benthamiana* leaves expressing P1-GFP or P1^N116A^-GFP at 48 hpi. The fluorescence signals of P1 and P1^N116A^ were photographed using confocal microscopy at different times after MG132 or DMSO infiltration. The red fluorescence represents the nuclear signals of all cells and was used as a reference for the GFP signal. Bars indicate 50 μm. Three times the experiment was repeated with similar results. **d** Effect of MG132 on the stability of P1 and P1^N116A^ protein by semi-in vivo assay. P1 and P1^N116A^ at different times after 100 µM CHX and 20 mM ATP treatment in the presence of 100 µM MG132 or an equal volume of DMSO (control). Each lane represents a sample from an individual experiment; three times the experiment was repeated with similar results. Signal density of protein bands was quantified using Image J software; the values are the means ± SD. Ponceau staining shows sample loadings. Source data are provided as a Source Data file.

The N-glycosylation process is associated with the folding of proteins, and the accumulation of misfolded proteins beyond the scope of ER processing can cause ER stress, triggering the unfolded protein response (UPR). Thus, three typical representative genes involved in the UPR process, *BIP*^52^, *bZIP17*/*bZIP28*^53^ and *bZIP60*^54^, were selected for analysis of whether N-glycosylation of P1 affects ER stress in the host. First, P1-GFP, P1^N116A^-GFP, P1^N116A^-NLS-GFP, and GFP were expressed in *N. benthamiana* leaves, and the ER stress inducer TM and DMSO were used as positive and negative controls, respectively. qRT-PCR analysis revealed that the expression levels of *NbBIP*, *NbbZIP17* and *NbbZIP60* were significantly upregulated at 12, 24 and 48 h after TM treatment, and there were no significant differences in the expression levels of these three genes in DMSO-treated leaves at different time points (Fig. 6a). The expression levels of *NbBIP* and *NbbZIP60* with P1^N116A^-GFP infiltration was significantly upregulated at 24 and 48 hpi, respectively (Fig. 6a). Similar to GFP-infiltrated leaves, P1-GFP also did induce expression of *NbBIP* and *NbbZIP60*, but the magnitude of changes was quantitatively significantly lower compared with P1^N116A^ (Fig. 6a), and P1^N116A^-NLS-GFP also led to a slight upregulation of *NbBIP* (Supplementary Fig. 7c). Moreover, the expression levels of *TaBIP*, *TabZIP28*, and *TabZIP60* were also detected in wheat plants inoculated with WYMV or WYMV(P1^N116A^) at 12, 24, 48 hpi. The results showed that the expression levels of *TaBIP* and *TabZIP60* were obviously upregulated under WYMV(P1^N116A^) infection, and their expressions were slightly upregulated under WYMV compared with before inoculation (Supplementary Fig. 7d). These findings suggest that nonglycosylated P1 may not fold correctly and thus trigger ER stress in plants.

Plants usually remove misfolded proteins through the ER-associated degradation pathway to alleviate ER stress^55^. To confirm and characterize the P1 and P1^N116A^ degradation processes, we performed in vivo degradation assays using wheat protoplasts. Compared with the DMSO control, treatment with MG132 led to markedly increased protein accumulation levels of P1-GFP, P1^N116A^-GFP, and P1^N116A^-NLS-GFP, especially P1^N116A^-GFP. In contrast, no obvious increase was detected upon treatment with Wortmannin (the agent of blocking autophagy formation) (Fig. 6b, Supplementary Fig. 7e). These results indicated that P1^N116A^ is predominantly degraded via the 26S proteasome pathway. Furthermore, the CHX, together with MG132 or an equal volume of DMSO, was infiltrated into *N. benthamiana* leaves expressing P1-GFP or P1^N116A^-GFP to further detect the proteasome-dependent degradation in vivo. The fluorescence signals of P1-GFP and P1^N116A^-GFP in MG132-treated leaves were slightly stronger than those in DMSO-treated leaves at the same time points (Fig. 6c). Moreover, total protein containing P1-GFP or P1^N116A^-GFP was analyzed with an anti-GFP antibody at different times after treatment with the protein translation inhibitor cycloheximide (CHX) in the presence or absence of ATP at 25 °C. 35S:GFP was used as the control. Supplementary Fig. 7f showed that P1^N116A^ was degraded more quickly than P1 in protein extracts with ATP, and 35S:GFP was not markedly degraded under any treatment. These results verified that the N116A mutation affected P1 protein stability. To determine how these proteins are degraded, in the presence of ATP, CHX together with MG132, or an equal volume of DMSO (control), was added to the total protein containing P1-GFP or P1^N116A^-GFP, following the same treatment conditions. Compared with those in the DMSO-treated samples, P1^N116A^ protein accumulation levels were clearly increased in the MG132-treated samples at the same time point, and a slightly greater accumulation of P1 was detected in the MG132-treated samples than in the DMSO-treated samples (Fig. 6d). In addition, we found that P1^N116A^ has higher ubiquitination levels than P1 (Supplementary Fig. 7g).

### The natural variants of *TaOST1B* affect the N-glycosylation and stability of P1

**Fig. 7:**
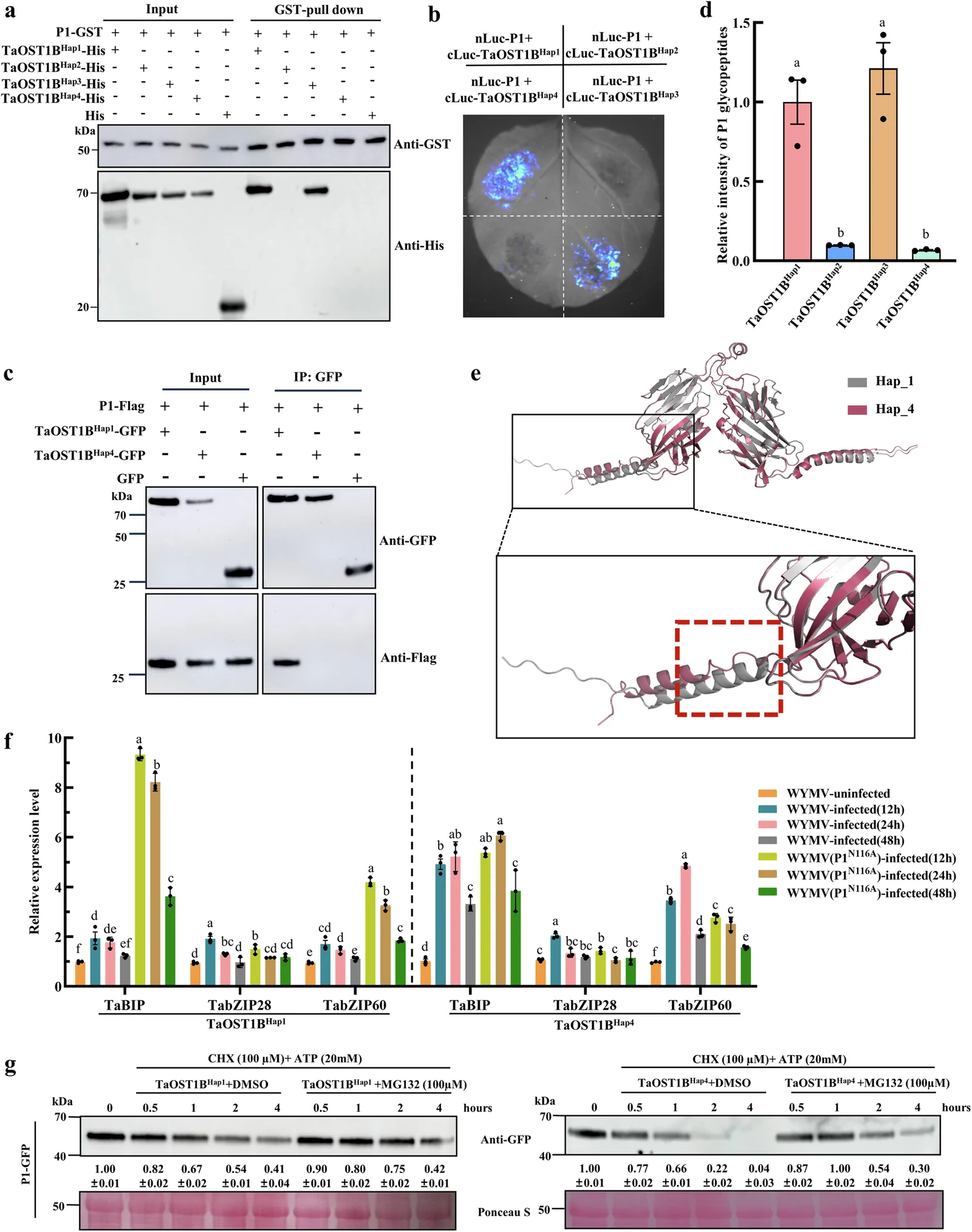
Natural variation of *TaOST1B* affects its regulation of WYMV infection. **a** GST pull-down assay was used to investigate the interaction between P1-GST and TaOST1B^Hap1^-His, TaOST1B^Hap2^-His, TaOST1B^Hap3^-His or TaOST1BHap^4^-His in vitro. His was used as a control. Three times the experiment was repeated with similar results. **b** Interaction of P1 with TaOST1B^Hap1^, TaOST1B^Hap2^, TaOST1B^Hap3^ or TaOST1B^Hap4^ were detected by LCI assay on *N. benthamiana* leaves. Luciferase activity was captured using a low-light cooled CCD imaging apparatus at 2 dpi. **c** Co-IP analysis of the interactions of P1 and TaOST1B^Hap1^ or TaOST1B^Hap4^ in wheat protoplasts. P1-Flag with GFP were the negative control groups, and P1-Flag with TaOST1B^Hap1^-GFP or TaOST1B^Hap4^-GFP were tested. The samples were collected 16 h after transfection, following which Co-IP with GFP beads was performed. Three times the experiment was repeated with similar results. **d** Analysis of N-glycosylation abundance of P1 in WYMV-infected wheat plants with *TaOST1B*^*Hap1*^, *TaOST1B*^*Hap2*^, *TaOST1B*^*Hap3*^ or *TaOST1B*^*Hap4*^. The intensity of P1 glycopeptides was examined by LC-MS/MS. The violin height represents the summed abundance of the P1 principal glycoforms on the site-specific level. Values are means ± SD (one-way ANOVA tests, *n* = 3 technically independent replicates), different letters represent significant differences, *P* < 0.01. **e** AlphaFold simulation analysis was performed to compare the protein model differences between TaOST1B^Hap1^and TaOST1B^Hap4^. The red box shows the differences in their N-termini. **f** The expression analysis of *TaBIP*, *TabZIP28*, and *TabZIP60*, ER stress-related representative genes by qRT-PCR in wheat plants with *TaOST1B*^*Hap1*^ or *TaOST1B*^*Hap4*^ before and after WYMV or WYMV(P1^N116A^) inoculation. *TaCDC* was used as the internal control gene. Values are means ± SD (one-way ANOVA tests, *n* = 3 biologically independent samples), different letters represent significant differences, *p* < 0.05. **g** Detection of P1 protein stability by semi-in vivo assay. P1-GFP was expressed in wheat protoplasts containing *TaOST1B*^*Hap1*^ or *TaOST1B*^*Hap4*^. The total protein extracts were treated with 100 µM CHX and 20 mM ATP in the presence of 100 µM MG132 or an equal volume of DMSO (control). Samples were detected at different times after treatment by western blotting. Each lane represents a sample from an individual experiment; three times the experiment was repeated with similar results. Signal density of protein bands was quantified using Image J software; the values are the means ± SD. Ponceau staining shows sample loadings. Source data are provided as a Source Data file.

To investigate the impact of natural variations in *TaOST1B* on the regulation P1, the four haplotypes-encoded proteins were all expressed in *E. coli* and ultimately purified (Supplementary Fig. 8a). A pull-down assay was performed to investigate the interaction differences in the interaction between P1 and these four proteins, and the results revealed that TaOST1B^Hap1^ and TaOST1B^Hap3^ could bind to P1, whereas TaOST1B^Hap2^ or TaOST1B^Hap4^ and P1 could not interact in vitro (Fig. 7a). The same results were obtained through the LCI assay in vivo (Fig. 7b). CoIP assay also confirmed the interaction between TaOST1B^Hap1^ and P1, while TaOST1B^Hap4^-GFP do not (Fig. 7c). Moreover, LC-MS/MS analysis showed that the relative intensity of P1 glycopeptides were significantly greater in WYMV-infected wheat plants with *TaOST1B*^*Hap1*^ or *TaOST1B*^*Hap3*^ genotype than in WYMV-infected wheat plants with *TaOST1B*^*Hap2*^ or *TaOST1B*^*Hap4*^ genotype (Fig. 7d, Supplementary Fig. 8b, c, Supplementary Data 5c), and the relative proportion of glycosylated P1 also showed a similar trend in these wheat plants (Supplementary Fig. 8d, Supplementary Data 6c). The variation of 2-amino acid deletion at positions 18 and 19 among these four haplotypes distinguishes their regulation P1. We further examined the interactions between P1 and the core domain Rophrin_I of TaOST1B^Hap1^ or TaOST1B^Hap4^. The result showed that both could interact with P1 (Supplementary Fig. 9a). However, when the corresponding N-terminal sequences were added to the Rophrin_I domain, TaOST1B^Hap1^ could still interact with P1, while the interaction of TaOST1B^Hap4^ with P1 was lost (Supplementary Fig. 9a). Further, we found that TaOST1B^Hap1^ with deletion mutation of the 18–19 amino acids also lost its interaction with P1 (Supplementary Fig. 9a), which suggested that the 2-amino acid at 18–19 position are important for the binding of TaOST1B to P1. Additionally, through protein model simulation, we discovered that the 2-amino acid deletion caused a change in the N-terminal helical structure of TaOST1B (Fig. 7e). Therefore, we speculated that this 2-amino acid deletion likely impairs binding through an allosteric effect on the protein’s overall folding. Then, wheat plants with *TaOST1B*^*Hap1*^ or TaOST1B^*Hap4*^ genotype were inoculated with WYMV or WYMV(P1^N116A^), respectively. The expression levels of *TaBIP*, *TabZIP28*, and *TabZIP60* were detected at 12, 24, and 48 hpi. We found that the expression levels of *TaBIP* and *TabZIP60* in wheat plants with *TaOST1B*^*Hap4*^ genotype were significantly upregulated under WYMV or WYMV(P1^N116A^) infection, whereas those in wheat plants with *TaOST1B*^*Hap1*^ genotype were markedly upregulated under WYMV(P1^N116A^) infection; their expressions were slightly upregulated at 12 hpi or 24 hpi and then restored to no significant differences at 48 hpi compared with before inoculation WYMV (Fig. 7f). Subsequently, wheat plants with *TaOST1B*^*Hap1*^ or *TaOST1B*^*Hap4*^ genotype were selected to detect P1 degradation, respectively. The ubiquitination of P1 in wheat plants with *TaOST1B*^*Hap1*^ genotype was weaker than that in wheat plants with *TaOST1B*^*Hap4*^ genotype, whereas the ubiquitination of P1^N116A^ was greater than that of P1 in wheat plants with *TaOST1B*^*Hap*^ genotype (Supplementary Fig. 9b). The semi-in vivo degradation assay revealed that the degradation rate of P1 in wheat plants with *TaOST1B*^*Hap1*^ genotype was lower than that in wheat plants with *TaOST1B*^*Hap4*^ genotype (Fig. 7g). These findings indicated that 2-amino acid deletion at positions 18 and 19 of TaOST1B could not bind to P1 for its N-glycosylation, thereby triggering ER stress and leading to the degradation of P1 via the 26S proteasome pathway.

## Discussion

N-glycosylation involves connections between glycans and protein molecules, which are necessary for the correct folding, stability maintenance, and intracellular localization of protein^56^. The OST complex-mediated transfer of core oligosaccharides is a central step in N-glycosylation^57^. The OST complex includes different subunits, which are responsible for substrate recognition, catalysis, and structural stability. Some of which have been reported to be involved in plant development and pathogen defense or pathogenicity. Mutation of OsDGL1, an ortholog of the yeast WBP1 subunit of the OST complex in rice, resulted in a shorter root cell length, a smaller root meristem, and cell death in the root^58^. The OST3/6 subunit specifically regulates interspecific gametophyte recognition in *Arabidopsis*^59^. OsDGL1 and OST3/6 are intrinsically required for plant growth and development. TaSTT3b-2B is specifically expressed after being attacked by *Rhizoctonia cerealis*; it resists pathogen infection by regulating the expression of a series of defense-related genes in wheat^60^. STT3 mediates full virulence through the regulation of fungal development, hyphal growth, and glycoprotein secretion in *Verticillium dahliae*^61^. In our study, TaOST1B, a subunit of the OST complex, was identified as associated with WYMV resistance through GWAS and fine mapping. It was specifically induced by WYMV (Fig. 1) and positively regulated WYMV infection in wheat (Fig. 2). This is the first investigation to uncover the positive role played by the OST subunit in plant viral pathogenicity. RNA viruses are obligate intracellular infectious pathogens; they usually hijack certain host factors to facilitate their own reproduction. TaOST1B^Hap1^should be required for the WYMV successful infection, as it can specifically interact with the WYMV-encoded P1 and regulate its N-glycosylation intensity (Figs. 3, 7). Whereas, there are differences in the N-terminal signal peptide sequences between TaOST1B^Hap4^ and TaOST1B^Hap1^, leading to alterations in the signal peptide cleavage site and consequently generating distinct mature N-termini (Supplementary Fig. 9c), which may affect the interaction of TaOST1B and P1. Moreover, P1 localizes to the nucleus and associates with the ER membrane, whereas N-glycosylation mediated by the OST complex occurs in the ER lumen. Although P1 lacks a canonical signal peptide, we speculate that it may access the ER lumen and undergo N-glycosylation during infection through non-canonical or host-assisted mechanisms. Similar ER lumen targeting and glycosylation have been reported for several viral proteins, such as hepatitis C virus E1/E2 and flavivirus NS1, which rely on complex membrane-associated or polyprotein-processing mechanisms^62,63^. Studies have reported that the OST1 can recognize nascent polypeptides that are destined to be glycosylated in yeast; its two luminal domains, NTD1 and NTD2, can immobilize glycosylated peptides^15,64^. The interaction between TaOST1B and P1 suggests that it may be responsible for recognizing the nascent peptide of P1. However, the potential roles of other OST subunits in the P1 N-glycosylation process and the mechanism by which N-terminal variation in TaOST1B mediates its interaction with P1 require further investigation.

In plant-virus coevolution, viruses can hijack host factors to functionally modify viral proteins to facilitate infection; such host factors are known as susceptibility (*S*) genes, which are essential for the pathogenicity of viruses. Resistance mediated by *S* gene mutations usually has the potential durability and broad spectrum. Thus, modification of the *S* gene constitutes an important strategy to improve crop resistance^65^. For example, the mutation of all three *TaMLO* homeologs confers heritable broad-spectrum resistance to powdery mildew in wheat^66^. The effector PsSpg1, which is secreted by *Puccinia striiformis* f. sp. *tritici* (*Pst*), can bind receptor-like kinase TaPsIPK1, increasing its kinase activity and nuclear localization to promote *Pst* parasitism, whereas *TaPsIPK1* knockout confers robust rust resistance^67^. Directionally modifying the N-glycosylation site of the maize immune receptor ZmLecRK1 confers resistance to *Fusarium graminearum* by preventing its targeting by the effector FgLPMO9A^30^. In this study, *TaOST1B*^*Hap1*^−2B was a host susceptibility gene required for WYMV successful infection, and knocking out *TaOST1B* can enhance wheat resistance to WYMV (Fig. 2). Moreover, we found that the triple mutant lines *taost1b* exhibited markedly slower seedling emergence compared to wild-type and single mutant lines (Supplementary Fig. 10a), but no notable differences were observed in panicle length, seed size, and thousand-grain weight among these lines (Supplementary Fig. 10b–d). Plant height and spikelets per panicle were markedly reduced in *taost1b* lines compared to wild-type and single mutants, while tiller number was significantly higher in *taost1b*, *taost1b*−2B, and *taost1b*−2A lines compared to wild-type and *taost1b*−2D lines (Supplementary Fig. 10e–h). The effects of these growth processes were predominantly observed in the triple mutant, but not in the single-copy knockout lines. These results suggest functional redundancy among the three TaOST1B homeologs in wheat development. Specifically, *TaOST1B*−2A and *TaOST1B*−2D may partially compensate for the loss of *TaOST1B*−2B in wheat growth processes, although their precise regulatory pathways and molecular mechanisms require further investigation. In addition, we also found that there are no obvious differences for agronomic traits at maturity among wheat plants corresponding to the four haplotypes of *TaOST1B* (Supplementary Fig. 11). Although TaOST1B^Hap2/Hap4^, which has enhanced WYMV resistance, may be rare in wheat germplasm, it is valuable for improving viral resistance. Furthermore, we performed a Blast analysis on the orthologs with amino acid sequence identities exceeding 80% were found mainly in cereal species, and all of them contain the conserved Ribophorin_I domain of the oligosaccharyltransferase (Supplementary Fig. 12a). Therefore, we speculate that TaOST1B is likely a susceptibility protein that evolved under the specific selective pressure of cereal-infecting viruses. We extended our analysis to other related viruses, including Wheat spindle streak mosaic virus (WSSMV), Barley yellow mosaic virus (BaYMV), and Barley mild mosaic virus (BaMMV), which share phylogenetic proximity and host range with WYMV. Sequence alignment revealed that the asparagine residue at position 116 of P1 is highly conserved in the homologous proteins encoded by these viruses (Supplementary Fig. 12b). Online prediction via the NetNGlyc 1.0 server revealed that VSRs encoded by these viruses all contain potential N-glycosylation sites. This leads us to hypothesize that OST-mediated glycosylation may be a conserved virulence strategy employed by most viruses. Moreover, we found that TaOST1B^Hap1^ in relation to CWMV also enhanced CWMV infection in wheat (Supplementary Fig. 12c–e), which suggested that TaOST1B-mediated glycosylation could be utilized by CWMV. Therefore, we propose that most viruses require host OST-mediated N-glycosylation to facilitate successful viral infection, which might make the host OST a promising broad-spectrum antiviral target.

Plants can resist viral infection through fundamental RNAi immune defense, whereas plant viruses encode at least one VSR to inhibit this antiviral response through various mechanisms. The VSR activity of P1 might be associated with its subcellular distribution^35^. Our study revealed that P1 was well distributed in the cytoplasm and nucleus of *N. benthamiana* epidermal cells, whereas the nonglycosylated P1^N116A^ presented a granular structure in the cytoplasm and a few nuclei (Fig. 4c, Supplementary Fig. 5d). P1^N116A^ lost VSR activity, which was restored after enforced localization of P1^N116A^ into the nucleus (Fig. 4f, g). Moreover, the pathogenicity of the WYMV(P1^N116A^) was almost completely lost after infection of the host (Fig. 4a, b). In contrast, virulence was markedly increased after supplementation with P1^N116A^-NLS (Fig. 4e). These findings indicate that the nuclear localization of P1 is necessary for its VSR activity and the virulence of WYMV. Similarly, nuclear targeting of the 17K protein in BYDV is likely important for its ability to suppress host antiviral RNAi defense^68,69^. The nuclear transport receptor HvIMP-α can interact with 17K, and the mutations in *TaIMP-α1* and *TaIMP-α2* increase resistance to BYDV in wheat^50^. P1 was not localized in the nucleus when lacking TaIMP-α2, and the addition of TaIMP-α2 restored its nuclear localization (Fig. 5d–g). Importin-α typically recognizes the NLS of protein and guides it into the nucleus through the nuclear pore complex^70^. P1 contains an NLS by prediction (cNLS Mapper). In our study, P1 and P1^N116A^-NLS, but not P1^N116A^, interacted with TaIMP-α2 (Fig. 5a–c, Supplementary Fig. 7a), indicating that the primary effect of nonglycosylation is on the nuclear targeting of P1, rather than on its overall protein folding. An early study on VSR revealed that a calmodulin (CaM)-like protein can regulate antiviral RNAi in plants^71^. A recent study revealed that CaM3 can activate CaMTA3, a CaM-binding transcriptional activator3, which subsequently targets the promoters of two essential genes, *RNA-dependent RNA polymerase6* (*RDR6*) and *Bifunctional nuclease2* (*BN2*), in the RNAi pathway and stimulates their transcription^72,73^. WYMV-encoded P1 was identified as a VSR interfering with the interaction between NbCaM and NbCaMTA3 to suppress RNA silencing^36^. Consistent results were also obtained in this study. The nucleus-localized P1, but not its cytoplasmic counterpart, could bind to CaMTA3 in the nucleus (Fig. 4h, i). This interaction disrupted the CaM3-CaMTA3 complex, thereby downregulating *RDR6* expression (Fig. 4j, Supplementary Fig. 6d). Collectively, these findings indicated that glycosylation-mediated nuclear import of P1 allows it to access and disrupt the interaction of CaMTA3-CaM3, thereby executing its VSR function.

Underglycosylation typically causes accumulation of protein aggregates in the ER lumen, leading to an imbalance in ER homeostasis, which triggers ER stress^74^. Eukaryotic cells can ease ER stress through different strategies, including the UPR, ER-associated degradation (ERAD), and autophagy, which promote protein folding or eliminate abnormal proteins to restore ER function^75^. BIP plays an important role in assisting protein folding and in the processes of ERAD^76^. The expression of UPR genes is regulated by two pivotal ER-located transcription factors, *bZIP28*/*bZIP17* and *bZIP60*, in *Arabidopsis*^53,77^. In this study, P1^N116A^ displayed obvious aggregation in cells (Figs. 4c, 5d). *NbBIP* and *NbbZIP60*, a chaperone and a transcription factor associated with the ER stress pathway, were significantly more highly transcribed in *N. benthamiana* leaves expressing P1^N116A^ than in those expressing P1 (Fig. 6a). Moreover, the expression of *TaBIP* and *TabZIP60* was significantly upregulated in wheat plants with *TaOST1B*^*Hap4*^ genotype under WYMV or WYMV(P1^N116A^) infection (Fig. 7f). This may be due to the inability of underglycosylated P1 to fold correctly, causing cellular ER stress that activates *bZIP60-*regulated UPR. ERAD is a conserved and multistep process involving substrate recognition, delivery to the cytoplasm, and ubiquitination and degradation by the 26S proteasome^78^. The E3 ligase hydroxymethylglutaryl reductase degradation 1 (HRD1), a core component of the ERAD pathway, can interact with the TGB1-3 protein encoded by beet necrotic yellow vein virus and ubiquitinate TGB1-3, thereby facilitating its degradation through the 26S proteasome to inhibit viral infection^79^. A recent study reported that the ER membrane-localized protein ZmNLD interacts with RING domain-containing E3-ubiquitin ligases to form a branch of the ERAD pathway independent of classic HRD1 in maize^80^. Our study revealed that nonglycosylated P1^N116A^ has stronger ubiquitination and faster 26S proteasome-mediated degradation rates than N-glycosylated P1 (Figs. 6b, c, 7g, Supplementary Figs. 7e, f, 9b). These results suggested that P1 can evade the host’s ubiquitination and subsequent degradation by undergoing N-glycosylation. However, how glycosylation and ubiquitination coordinately regulate WYMV infection in wheat, as well as the host factors and regulatory mechanisms underlying the ubiquitination of nonglycosylated P1, remain future directions.

**Fig. 8:**
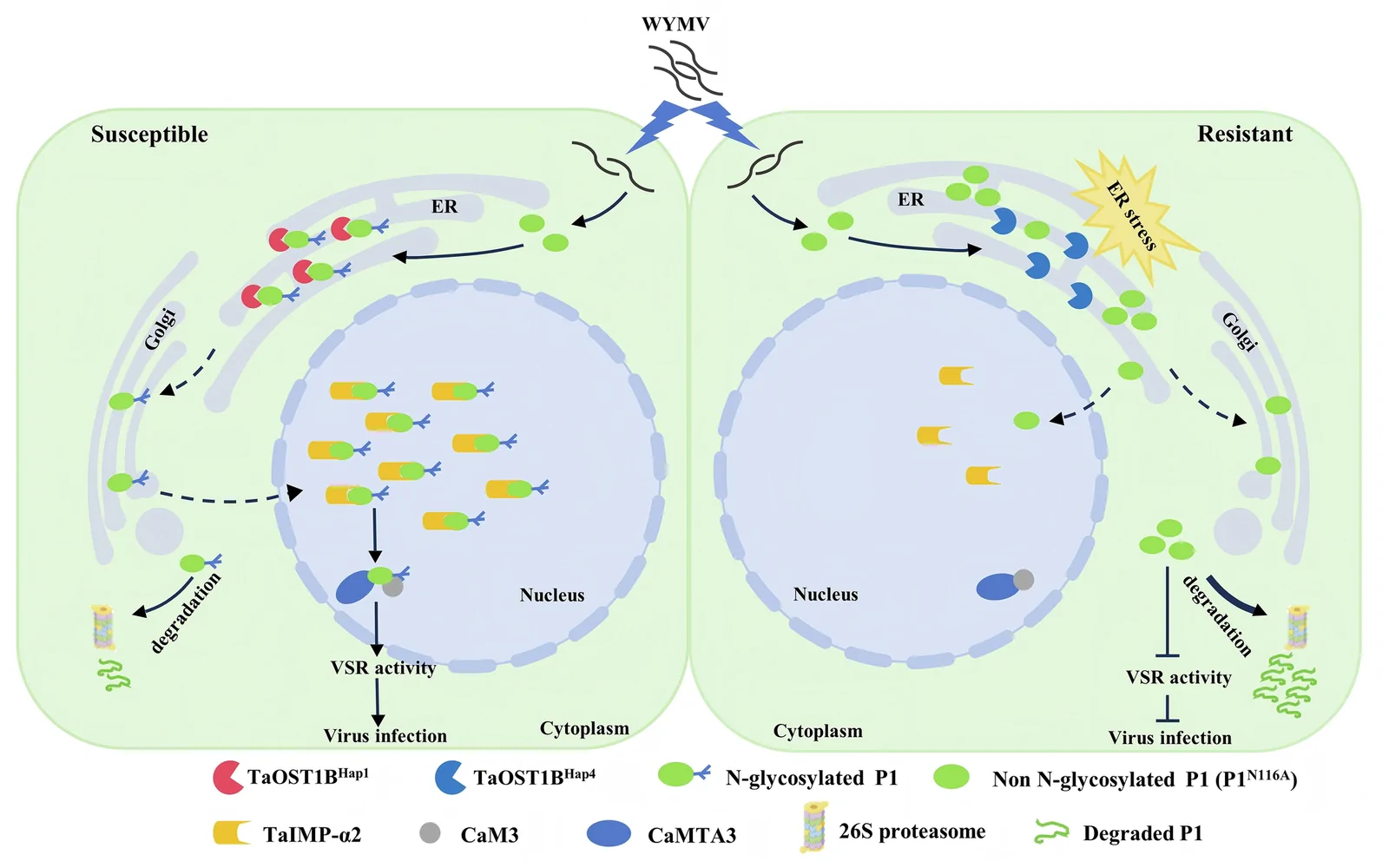
Model for natural variant of the oligosaccharyltransferase *TaOST1B* improves resistance to virus by disrupting the viral hijacking in wheat. During WYMV infection, TaOST1B^Hap1^ localized in the ER could bind and N-glycosylate P1 protein, N-glycosylated P1, interact with the nuclear transport protein TaIMP-α2 and successfully transport into the nucleus to disrupt CaM3-CaMTA3 interaction, thereby function as the VSR to facilitate WYMV infection. While TaOST1B^Hap4^ is incapable of binding to P1 resulting in fail for N-glycosylating, which causes P1 gathering in the ER and sequentially triggering ER stress to degrade nonglycosylated P1 by 26S proteasome, thereby losing VSR function and inhibiting WYMV infection.

In summary, we propose a model illustrating a form of viral pathogenesis in plants. During WYMV infection, the susceptibility TaOST1B^Hap1^ located in the ER can interact with WYMV P1 to N-glycosylate it, which promotes the binding of P1 to the nuclear transport protein TaIMP-α2 and enhances its nuclear accumulation. N-glycosylation serves as a specific signal for P1 nuclear trafficking, ensuring the nuclear-localized P1 binds to CaMTA3, disrupting the CaM3-CaMTA3 interaction, thereby exerting VSR function to facilitate WYMV infection. The natural variant TaOST1B^Hap4^, lacking the ability to bind and N-glycosylate P1, causes the accumulation of underglycosylated P1 in the ER and loss of VSR function, triggering ER stress to degrade nonglycosylated P1 via the 26S proteasome, thereby limiting WYMV infection (Fig. 8). This finding enriches our understanding of the function of oligosaccharyltransferase in plant-virus interactions.

Limitations of the study: TaOST1B is an oligosaccharyltransferase subunit that mediates N-glycosylation of P1. However, the precise molecular mechanism underlying its recognition of the P1 nascent peptide remains unclear. Future studies should employ structural analyses to elucidate their binding mechanism. Additionally, the WYMV resistance-enhanced haplotypes TaOST1B^Hap2^/TaOST1B^Hap4^ are rare in wheat germplasms, which may constrain their direct breeding utility. Nevertheless, they represent promising targets for wheat improvement through strategies such as marker‑assisted selection and gene editing.

## Methods

### Plant materials and growth conditions

In this study, 480 wheat (*Triticum aestivum*) accessions composed of 106 historical varieties, 243 seedling strains and 131 varieties from HuangHuai Valley as the associated population^49,50,81^ were used to perform GWAS. All plants were grown in a WYMV nursery field in Xiping (33.40 °N, 113.3 °E) during 2016/2017 (one replication), 2017/2018 (two replications), and 2018/2019 cropping seasons (two replications). A residual heterozygous line (RHL) population resulting from UC1110 and PI610750 (UP-RIL) composed of 453 lines for fine mapping. The UP-RHL populations were also planted in the same field in Xiping with two replications. The highly susceptible variety Xinmai 18 was planted every 10th row as the control. Disease severity was investigated at the jointing stage when infection of Xinmai 18 had reached the infection type (IT) level 3^49,50^. All materials are used in conventional field management practices.

Wheat cv. Yangmai 158 (Susceptible cultivar) was used to investigate instantly the roles of the candidate gene. Wheat cv. Fielder (middle-resistant cultivar) was used to generate *TaOST1B* expression and *TaOST1B* knockout transgenic lines. The WYMV infectious clones pCB301-35S-R1 and pCB301-35S-R2 were obtained from a published source and were used to infect wheat plants^82^. The inoculated plants were grown in a growth chamber maintained at 8 °C. For field assessments, the *TaOST1B* transgenic and knockout lines were grown in a nursery with WYMV in Yangzhou, China.

### Genotyping and genome-wide association study

The 480 wheat accessions were genotyped using the wheat Pan800K array. After quality control, only markers with minor allele frequency (MAF) > 0.05 and missing data <20% in the association panel were kept for GWAS analysis using PLINK software^83,84^. The GWAS analysis was implemented using the GAPIT package of R software and was based on a mixed linear model (PCA + K)^85,86^. The threshold of the significant *P*-value was set as 1.0E-4 for the associated population. TASSEL v5.2 software was used to analyze linkage disequilibrium (LD) and chromosome decay distance of Chr. 7B^87^. r^2^ values were estimated for all pairwise marker comparisons with a sliding window size of 500 SNPs. The LD decay over genetic distance was calculated by fitting a nonlinear model with the modified Hill and Weir approach^88^, with the r^2^ threshold set at 0.3.

### Fine mapping

The UC1110 and PI610750 population was genotyped using 695 markers. 11 additional markers were developed, including 4 kompetitive allele specific PCR (KASP) and 7 insertion/deletion (InDel) markers. Linkage mapping was performed using IciMapping 4.1. PVE was used to evaluate the genetic effects of the identified QTLs. The threshold of the LOD score was set to 2.5.

### Barley stripe mosaic virus-induced gene silencing

The 200-bp fragment from the conserved coding region of four candidate genes was reversely inserted into the plasmid pBSMV-γ to produce the recombinant vector pBSMV-γTaOST1B for silencing genes; all primers are listed in Supplementary Data 7. The linearized plasmid (pBSMV-α, pBSMV-β, pBSMV-γ, and pBSMV-γTaOST1B) was prepared for obtaining the capped transcripts through a Message T7 in vitro transcription kit (Ambion, Austin, TX; Promega, Shenzhen, China) following the manufacturer’s instructions. The resulting transcripts were mixed and then diluted with 7 μL inoculation buffer (0.06 M potassium phosphate, 0.1 M glycine, 1% bentonite, 1% sodium pyrophosphate decahydrate, 1% celite, pH 8.5) in a ratio 1:1:1:7. The second fully unfolded leaves of Yangmai 158 with two-leaf stage were rub-inoculated with BSMV:TaOST1B. BSMV:TaPDS and BSMV:00 were used as references. The fourth fully expanded leaves with visible viral infection symptoms were collected and inoculated with WYMV. At 14 dpi, the system leaves were collected for testing the accumulation level of WYMV to evaluate disease resistance.

### qRT-PCR analysis

Total RNAs were extracted from seedling leaves of the wheat plants using the plant RNA extraction kit (Magen, R4165-02), then reverse transcribed into cDNAs by the HiScript II 1st Strand cDNA Synthesis Kit (Vazyme Biotech Co., Ltd.) following the manufacturer’s protocol. Expression level of genes was detected using AceQ qPCR SYBR Green Master Mix (Vazyme) through ABI Q5 Sequence Detection System (Applied Biosystems, Foster City, CA, USA). Each qRT-PCR assay was repeated at least three times with three independent RNA preparations and *TaCDC*, cell division cycle gene (XM_020313450 [https://www.ncbi.nlm.nih.gov/nuccore/2158015422]) or *NbActin*, was used as internal reference genes for analysis to calculate the fold changes in gene expression. The comparative 2^–ΔΔCT^ method was used to quantify relative gene expression. All gene-specific primers for qRT-PCR are shown in Supplementary Data 7.

### Western blot analysis

Fresh leaf tissues were ground into a fine powder in liquid nitrogen and then added moderate extraction buffer (100 mM Tris-HCl, pH 8.8, 60% SDS and 2% β-mercaptoethanol), the cells were fully lysed by vortexing and left on ice for 30 min. The extracts were centrifuged at 13,523×*g* for 10 min at 4 °C, supernatants were transferred to new microcentrifuge tubes, and 5× SDS loading buffer was added (1:4, v/v), and then incubated at boiling water for 10 min. Prepared protein samples were separated in SDS-PAGE gels through electrophoresis, and then transferred onto nitrocellulose membranes. The blots were incubated in a blocking buffer (5 % skim milk in 1× PBS) for 60 min and followed by detection with specific primary antibodies and then a horseradish peroxidase (HRP)-conjugated anti-mouse or anti-rabbit secondary antibody. These antibodies were anti-CP and anti-P1 (1:2000, prepared by Huaan Bio, Hangzhou, Zhejiang, China, stored in our lab), anti-GFP (1:5000, TransGen Biotech, Beijing, China, Cat. No. HT801), anti-Flag (1:5000, TransGen Biotech, Beijing, China, Cat. No. HT201), anti-His (1:5000, TransGen Biotech, China, Cat. No. HT501), anti-GST (1:5000, TransGen Biotech, China, Cat. No. HT601), anti-actin (1:10000, Huaan Bio, Hangzhou, Zhejiang, China, Cat. No. ET1701-80), anti-H3 (1:10000, Huaan Bio, Hangzhou, Zhejiang, China, Cat. No. ET1701-64), anti-Ub (1:3000, Abcam, United Kingdom, Cat. No. ab230145), anti-Mouse (1:5000, Abbkine, USA, Cat. No. A21010), anti-Rabbit (1:5000, Abbkine, USA, Cat. No. A21020). Detection signal was visualized using an Amersham Imager 680 machine (GE Healthcare BioSciences, Pittsburgh, PA, USA).

### Prediction of N-glycosylation sites

The online website (https://services.healthtech.dtu.dk/services/NetNGlyc-1.0/), which examines the amino acid sequence with N-X-S/T motifs (where X is any amino acid except Proline), was used to predict potential N-glycosylation sites in WYMV-encoded proteins.

### Plasmid construction

The full-length coding sequences (CDS) of WYMV P1, P2, P3, Vpg, CP, NIb, 14K, 7K, NIa, and CI were respectively amplified from a WYMV infectious clone. TaOST1B^Hap1^, TaOST1B^Hap2^, TaOST1B^Hap3^ and TaOST1B^Hap4^ were cloned from Chinese Spring, Yanmai998, Yangmai158 and Jimai22 cDNA, respectively. The mutant P1^N116A^, mutant infectious clones WYMV(P1^N116A^) were constructed using the overlap PCR. CDS without termination codon of P1, P2, P3, Vpg, CP, NIb, 14K, 7K, NIa, and CI were inserted into pCambia1300-nLuc, and the four haplotypes of TaOST1B were respectively inserted into pCambia1300-cLuc using an In-fusion Cloning kit (Takara Bio, Kusatsu, Japan). CDS without stop codon of TaOST1B, P1 and P1^N116A^ were inserted into pGWB505 (GFP-tag), pGWB454 (RFP-tag) and pGWB511 (Flag-tag) using the Gateway technology (Invitrogen) to generate TaOST1B^Hap1^-GFP, P1-GFP, P1^N116A^-GFP, P1-RFP, P1-Flag. TaOST1B^Hap1^-His, TaOST1B^Hap2^-His or TaOST1B^Hap3^-His and TaOST1B^Hap4^-His were constructed by inserting the CDS without a stop codon into pET-32a (+) via In-fusion cloning. P1-GST was constructed using the same method. All primers were listed in Supplementary Data 7.

### LCI assay

All pCambia1300-nLuc and pCambia1300-cLuc recombinant plasmids were separately transformed into *Agrobacterium* strain GV3101. *Agrobacterium* cells carrying cLuc-TaOST1B^Hap1^ (OD600 about 0.6) and an equal amount of *Agrobacterium* cells carrying nLuc-P1, nLuc-P2, nLuc-P3, nLuc-Vpg, nLuc-CP, nLuc-NIb, nLuc-14K, nLuc-7K, nLuc-NIa, or nLuc-CI were mixed, respectively. *Agrobacterium* cells carrying TaOST1B^Hap4^-cLuc (OD600 about 0.6) and an equal amount of *Agrobacterium* cells carrying P1-nLuc were mixed and then infiltrated into leaves of *N. benthamiana*. After 72 h, the same leaves were incubated with 0.2 mM luciferin (Thermo Scientific, USA) dissolved in ddH_2_O supplemented with 0.01% Triton X-100 at room temperature for 5 min. The luciferase activity was subsequently observed via a low-light cooled charge-coupled device (CCD) imaging system (SOPHIA 2048B). cLuc-TaOST1B^Hap1^+nLuc and cLuc+nLuc-P1 were used as negative control, cLuc-SAR+nLuc-SGT1 were used as positive control.

### Yeast two-hybrid assay

Y2H assays were performed following the method described in the Takara protocol handbook (Takara Bio Inc, Japan). In brief, yeast cells (AH109) carrying the co-transformed plasmids (AD-TaOST1B^Hap1^/BD-P3, BD-Vpg, BD-CP, BD-NIb, BD-14K, BD-NIa, BD-CI, BD-7K, BD-P2, BD-P1, AD-TaOST1B^Hap1^/BD, AD/BD-P1) were plated onto a low stringency selective medium lacking tryptophan and leucine (SD/-Trp-Leu) to confirm the transformation and then plated onto a high stringency selective medium lacking tryptophan, leucine, histidine, and adenine (SD/-Trp-Leu-His-Ade) to analyze the interaction. Transformants carrying pGBKT7-Lam and pGADT7-RECT were used as negative controls, and pGBKT7-53 and pGADT7-RECT were used as the positive control.

### Co-IP assay

To verify the interaction between TaOST1B and P1, TaOST1B-GFP and P1-Flag were expressed in the leaves of *N. benthamiana* by agroinfiltration, GFP was used as control. Total proteins were extracted using lysis buffer (10 mM Tris-Cl pH 7.5, 150 mM NaCl, 0.5 mM EDTA, 0.5 % Nonide P40 Substitute, 1× protease inhibitor cocktail) at 48 h after agroinfiltration, and co-immunoprecipitation was then performed using Anti-Flag® M2 magnetic beads (MilliporeSigma). The samples were detected by western blotting using an anti-GFP antibody (1:5000, TransGen Biotech, Beijing, China, Cat. No. HT801).

### Pull-down assay

Purified TaOST1B^Hap1^-His, TaOST1B^Hap2^-His, TaOST1B^Hap3^-His or TaOST1B^Hap4^-His with purified P1-GST were co-incubated at 4 °C in a 1× PBS buffer containing 1 mM PMSF for 1 h, His was used as a control. 25 μL immobilized glutathione beads were then added into each sample, followed by another 1 h incubation at 4˚C. After three to five rinses in 1× PBS buffer, 2× SDS sample buffer was added to the beads of each sample. The samples were boiled for 10 min and then analyzed through western blot assays using an anti-His antibody (1:5000, TransGen Biotech, Beijing, China, Cat. No. HT501).

### Subcellular localization

Through the *Agrobacterium*-mediated transient expression system, TaOST1B^Hap1^-GFP and ER-mCherry or P1-RFP were co-infiltrated into the leaves of *N. benthamiana*. TaOST1B^Hap1^-GFP, P1-GFP and P1^N116A^-GFP were individually infiltrated into H2B-RFP transgenic *N. benthamiana* leaves; GFP was used as a control. The fluorescence signals were observed and photographed 2 days after infiltration using a confocal microscope (Leica TCS SP8 X).

### N-glycosylation analysis of P1

To confirm the N-glycosylation of P1 protein, immunoprecipitation of P1-GFP and P1^N116A^-GFP was performed using the ChromoTek GFP-Trap® Magnetic Agarose (Proteintech) following the instructions. Immunoprecipitation of P1 from WYMV-infected wheat plants was performed using antigen affinity-purified P1 antibody coupled with Protein A/G Magnetic Beads (Vazyme) following the instructions. Immunoprecipitated proteins were collected using acidic elution buffer (200 mM glycine, pH 2.5) and neutralization buffer (1.5 M Tris-HCl, pH 8.5). Then, the eluted proteins were treated with PNGase F, PNGase A or EndoH (New England Biolabs) at 37 °C for 1 h, as described by the manufacturer. Reaction buffer (50 mM Tris-Cl pH 7.5, 10% Nonidet P-40, 0.5% sodium dodecyl sulfate, 40 mM dithiothreitol) was used as a control.

### Protein chymotrypsin digestion, purification, and glycopeptide enrichment

Coomassie-stained P1 bands from 11 samples were respectively cut across the entire length of the preparative gel, and subjected to in-gel chymotrypsin digestion according to standard protocols with minor modifications. Briefly, excised bands were cut into 1 mm^2^ cubes and destained in 50 mM ammonium bicarbonate (NH_4_HCO_3_, Sigma) in 50% acetonitrile (Sigma) before dehydration in 100% acetonitrile. In situ reduction and alkylation were achieved by incubating the gel pieces in 10 mM dithiothreitol (DTT, sigma) in 50 mM NH_4_HCO_3_ at 56 °C for 1 h, followed by incubation in 55 mM iodoacetamide (IAA, sigma) for 30 min at room temperature in the dark. The alkylated protein samples were then digested with chymotrypsin (Promega, V1062) at 37 °C overnight. After digestion, the supernatant was dried using a Speed Vacuum and desalted using a C18 tip. For glycopeptides enrichment, a homemade ZIC-HILIC stage-tip containing 15 mg of ZIC-HILIC particles (Merck Millipore) was used. The ZIC-HILIC stage tips were activated by washing three times with deionized water before equilibration with loading buffer (80% ACN/0.1% TFA, v/v). The dried peptide mixture was dissolved in 200 μL of loading buffer and loaded onto the stage tips three times at 1016×*g* for 10 min to maximize binding. Non-glycopeptides were removed from the HILIC particles by washing four times with loading buffer. Finally, the enriched glycopeptides were eluted with 300 μL of 0.1% TFA followed by 50 μL of 25 mM NH_4_HCO_3_ and 50 μL of 50% ACN. The combined eluents were dried using Speed Vacuum and stored before further analysis.

### Identification of N-glycosylation sites and N-glycopeptides by LC-MS/MS

The obtained glycopeptides were separated and analyzed using a Vanquish Neo UHPLC system coupled to an Orbitrap Eclipse mass spectrometer (Thermo Fisher Scientific). Each sample undergoes three technical replicates. The glycopeptides were dissolved in solvent A (0.1% formic acid in water) and loaded onto a C18 trap column (Thermo Scientific Acclaim PepMap 100, 75 μm × 2 cm, C18, 3 μm, 100 Å) and separated by the analytical column (Thermo Scientific Acclaim PepMap 100, 75 μm × 25 cm, C18, 2 μm, 100 Å) at a flow rate of 300 nL/min. The linear gradient was set as follows: 2–35% solvent B (0.1% formic acid in 80% acetonitrile) for 110 min, 35–50% solvent B for 12 min, 50–90% solvent B for 17 min, and maintained at 90% solvent B for an additional 10 min. The electrospray voltage was set at 2.2 kV, and the temperature of the ion transfer capillary was set at 320 °C. The mass spectrometer was operated in data-dependent acquisition mode with a cycle time of 3 s. Full MS scans were performed over m/z 375–2000 with a mass resolution of 120,000, and the RF lens set as 40%. The AGC target was set to 120 %, and the maximum injection time was set to 50 ms. For MS/MS, a defined isolation window of 4 m/z, first mass of 120 m/z, AGC target of 1000%, maximum injection time of 250 ms, and the stepped HCD collision energy of 20%, 30%, and 40% were used.

### Database searching for intact N-glycopeptides

The raw data were searched for glycopeptide matches using the pGlyco3 software. The search parameters were set as follows: protein modifications, carbamidomethylation (C) (fixed), oxidation (M) (variable), and acetyl [ProteinN-Term] (variable); enzyme specificity, chymotrypsin; maximum missed cleavages, 2; precursor ion mass tolerance, 10 ppm; and fragment tolerance, 20 ppm. The pGlyco glycan database named “pGlyco-N-plant-multi.gdb” was used for glycopeptide identification. The cutoff false discovery rate (FDR) for all peptide identifications was controlled below 1%. The N-glycopeptides that were identified by pGlyco3 software were further validated manually to ensure the accuracy of the results. The quantification results were obtained through the pGlycoQuant software. The quantification procedure was applied to the pGlyco identification results with MS1 spectral intensity. The quantification type was DDA label-free. All other parameters were the default. Prior to downstream analysis, the quantitative values obtained from pGlycoQuant were filtered, including missing value filtering and coefficient of variation (CV < 0.25) control. The intensities of all identified glycoforms were listed in Supplementary Data 5.

### Isolation of nucleoplasmic protein

The *N. benthamiana* leaves expressing P1-GFP or P1^N116A^-GFP were collected. The total proteins were extracted using fivefold volume of 0.4 M sucrose, 10 mM Tris (pH 8.0), 10 mM MgCl_2_, 5 mM β-mercaptoethanol, 0.1 mM PMSF and 1× cocktail (cOmplete™, EDTA-free protease inhibitor, Roche). The sample was vortexed and then set on ice for 30 min followed by applying miracloth filtration, 20 min centrifugation at 1500×*g*. The soluble supernatant was collected first for 10 min centrifugation, and again at 16,000×*g*, supernatants containing cytoplasmic proteins were collected. The sediment obtained by the first step of centrifugation was resuspended again in a fivefold volume of 0.25 M sucrose, 10 mM Tris (pH 8.0), 10 mM MgCl_2_, 1 % Triton X-100, 5 mM β-mercaptoethanol, 0.1 mM PMSF and 1× cocktail, and then centrifuged at 16,000×*g* for 10 min. The supernatants containing nuclear proteins were then collected and rinsed three times using 1.7 M sucrose, 10 mM Tris (pH 8.0), 2 mM MgCl_2_, 0.15% Triton X-100, 5 mM β-mercaptoethanol, 0.1 mM PMSF and 1× cocktail, 10 min centrifugation again at 16,000×*g*. The insoluble nuclear protein was prepared by adding 10 mM Tris (pH 7.5), 20 mM KCl, 2 mM EDTA, 2.5 mM MgCl_2_, 20% glycerinum, 250 mM sucrose and 5 mM dithiothreitol solution. 5× sample buffer was added (1:4, v/v) to each soluble cytoplasmic fraction or the insoluble nuclear protein fraction, followed by 10 min boiling. The resulting samples were analyzed via western blotting using anti-GFP (1:5000, TransGen Biotech, Beijing, China, Cat. No. HT801), anti-Actin and anti-Histone H3 antibody (1:10000, Huaan Bio, Hangzhou, Zhejiang, China, Cat. No. ET1701-80, ET1701-64).

### Semi-in vivo and in vivo protein degradation

For semi-in vivo protein degradation analysis, P1-GFP and P1^N116A^-GFP were expressed in *N. benthamiana* leaves by agroinfiltration and collected at 2 dpi; GFP was used as a control. The three samples were separately extracted with lysis buffer (10 mM Tris/Cl pH 7.5, 150 mM NaCl, 0.5 mM EDTA, 0.5 % Nonidet P40 substitute, 1× cocktail (cOmplete™, EDTA-free protease inhibitor, Roche)) as described above. For analysis of P1 and P1^N116A^ degradation by 26S proteasome, 20 mM ATP, 100 µM CHX, and 100 µM MG132 were added to the protein extracts, and DMSO was used as control. The mixtures were incubated at room temperature (25 °C) with agitation in an Eppendorf Thermomixer. Samples were removed at different time points and reactions stopped by the addition of 5× sample buffer (1:4, v/v) and boiled for 10 min for western blotting analysis. For in vivo analysis of P1 and P1^N116A^ protein degradation by the 26S proteasome, P1-GFP and P1^N116A^-GFP were expressed in *N. benthamiana* leaves by agroinfiltration, and the 100 µM CHX together with 100 µM MG132 in DMSO solution or an equal volume of DMSO control solution was infiltrated into the same leaves at 2dpi. Infiltrated leaves were observed and photographed at different time points after infiltration using a confocal microscope (Leica TCS SP8 X). The in vivo degradation assays using wheat protoplasts were performed with minor modifications based on the protocol reported by Mituła et al.^89^; the protoplasts expressing the target protein were treated with 50 µM MG132 or Wortmannin for 4 h, and DMSO was used as control.

### Reporting summary

Further information on research design is available in the Nature Portfolio Reporting Summary linked to this article.

## Supplementary information

Supplementary Information

Description of Additional Supplementary Files

Supplementary Data 1

Supplementary Data 2

Supplementary Data 3

Supplementary Data 4

Supplementary Data 5

Supplementary Data 6

Supplementary Data 7

Reporting Summary

Transparent Peer Review file

## Source data

Source Data

## Data Availability

Data supporting the findings of this work are available within the paper and its Supplementary Information files. The mass spectrometry proteomics data have been deposited to the ProteomeXchange Consortium via the iProX partner repository with the dataset identifier PXD079174 (https://www.iprox.cn/page/home.html). Source data are provided with this paper.
