# Supplementary material for: The oligosaccharyltransferase TaOST1B promotes viral infection by enhancement of RNA silencing suppression in wheat
Source: Nat Commun. 2026 Jul 15;17:8688. doi: 10.1038/s41467-026-75397-8 (PMC13490409; doi:10.1038/s41467-026-75397-8)
Supplement: Supplementary file 1 [file 41467_2026_75397_MOESM10_ESM.pdf]

nature portfolio

Corresponding author(s): Jianping Chen and Jian Yang

Last updated by author(s): June 25, 2026

Reporting Summary

Nature Portfolio wishes to improve the reproducibility of the work that we publish. This form provides structure for consistency and transparency in reporting. For further information on Nature Portfolio policies, see our Editorial Policies and the Editorial Policy Checklist.

Statistics

For all statistical analyses, confirm that the following items are present in the figure legend, table legend, main text, or Methods section.

n/a	Confirmed	
☐	☒	The exact sample size (*n*) for each experimental group/condition, given as a discrete number and unit of measurement
☐	☒	A statement on whether measurements were taken from distinct samples or whether the same sample was measured repeatedly
☐	☒	The statistical test(s) used AND whether they are one- or two-sided<br>*Only common tests should be described solely by name; describe more complex techniques in the Methods section.*
☒	☐	A description of all covariates tested
☒	☐	A description of any assumptions or corrections, such as tests of normality and adjustment for multiple comparisons
☐	☒	A full description of the statistical parameters including central tendency (e.g. means) or other basic estimates (e.g. regression coefficient) AND variation (e.g. standard deviation) or associated estimates of uncertainty (e.g. confidence intervals)
☐	☒	For null hypothesis testing, the test statistic (e.g. *F*, *t*, *r*) with confidence intervals, effect sizes, degrees of freedom and *P* value noted<br>*Give P values as exact values whenever suitable.*
☒	☐	For Bayesian analysis, information on the choice of priors and Markov chain Monte Carlo settings
☒	☐	For hierarchical and complex designs, identification of the appropriate level for tests and full reporting of outcomes
☒	☐	Estimates of effect sizes (e.g. Cohen's *d*, Pearson's *r*), indicating how they were calculated

Our web collection on statistics for biologists contains articles on many of the points above.

Software and code

Policy information about availability of computer code

Data collection	Nanotemper NT.115.<br>Confocal microscopic images were aquired with a TCS SP8 X confocal microscopy.<br>ThermoFisher QuantStudio 5 for qPCR data collection and analysis.<br>The raw data were searched for glycopeptide matches using the pGlyto3 software.
Data analysis	1. Microsoft excel<br>2. GraphPad Prism 8<br>3. SPSS 16.0 software<br>4. Image J<br>5. IciMapping 4.1<br>6. PLINK<br>7. GAPIT-R Package<br>8. TASSEL v5.2<br>9. pGlycoQuant<br>10. MEGA 11<br>11. Jalview

For manuscripts utilizing custom algorithms or software that are central to the research but not yet described in published literature, software must be made available to editors and reviewers. We strongly encourage code deposition in a community repository (e.g. GitHub). See the Nature Portfolio guidelines for submitting code & software for further information.

Data

Policy information about availability of data

All manuscripts must include a data availability statement. This statement should provide the following information, where applicable:
- Accession codes, unique identifiers, or web links for publicly available datasets
- A description of any restrictions on data availability
- For clinical datasets or third party data, please ensure that the statement adheres to our policy

Data supporting the findings of this work are available within the paper and its Supplementary Information files, or from the corresponding author upon request. Source data are provided with this paper.

Research involving human participants, their data, or biological material

Policy information about studies with human participants or human data. See also policy information about sex, gender (identity/presentation), and sexual orientation and race, ethnicity and racism.

Reporting on sex and gender	n/a
Reporting on race, ethnicity, or other socially relevant groupings	n/a
Population characteristics	n/a
Recruitment	n/a
Ethics oversight	n/a

Note that full information on the approval of the study protocol must also be provided in the manuscript.

Field-specific reporting

Please select the one below that is the best fit for your research. If you are not sure, read the appropriate sections before making your selection.

- [x] Life sciences
- [ ] Behavioural & social sciences
- [ ] Ecological, evolutionary & environmental sciences

For a reference copy of the document with all sections, see nature.com/documents/nr-reporting-summary-flat.pdf

Life sciences study design

All studies must disclose on these points even when the disclosure is negative.

Sample size	Sample sizes were not predetermined, but chosen to be similar to common sample sizes in previous studies in the field.
Data exclusions	We did not exclude any data points arbitrarily.
Replication	All attempts at replication were successful. The number of replicates is indicated in the corresponding figure legend and/or in the corresponding material and method section.
Randomization	All samples from each experiments was collected randomly for further analysis (e.g. qRT-PCR and western blot assay)
Blinding	All investigation were blinded to group allocation during data collection and analysis.

Reporting for specific materials, systems and methods

We require information from authors about some types of materials, experimental systems and methods used in many studies. Here, indicate whether each material, system or method listed is relevant to your study. If you are not sure if a list item applies to your research, read the appropriate section before selecting a response.

Materials & experimental systems

n/a	Involved in the study	
[ ]	[x]	Antibodies
[x]	[ ]	Eukaryotic cell lines
[x]	[ ]	Palaeontology and archaeology
[x]	[ ]	Animals and other organisms
[x]	[ ]	Clinical data
[x]	[ ]	Dual use research of concern
[ ]	[x]	Plants

Methods

n/a	Involved in the study	
[x]	[ ]	ChIP-seq
[x]	[ ]	Flow cytometry
[x]	[ ]	MRI-based neuroimaging

Antibodies

Antibodies used	1. WYMV CP-specific antibody, WYMV P1-specihc antibody, CWMV CP-specific antibody (prepared by Huaan Biotechnology Co., Ltd, Hangzhou, Zhejiang, China, and stored in our lab).<br>2. Flag, His, GST and GFP monoclonal antibody (TransGen Biotech, Beijing, China, HT201-01, HT501-01, HT601-01 and HT801-01).<br>3. Ubiquitin antibody (Abcam, Cambridge, United Kingdom, EPR8830).<br>4. Actin and H3 antibody (Huaan Biotechnology Co., Ltd, Hangzhou, Zhejiang, China, ET1701-80, ET1701-64).<br>5. Anti-Mouse and Rabbit (Abbkine Scientific Co., Ltd, California, USA, A21010 and A21020).
Validation	All antibodies expect for WYMV CP, P1 and CWMV CP are commercially available and validated in the literature as cited on the manufacturers websites as well as by the datasheets they provide. Antibody validation and validation criteria are available on the following websites:<br>Flag antibody:https://www.transgen.com/antibody_tag/371.html<br>His antibody: https://www.transgen.com/antibody_tag/385.html<br>GST antibody: https://www.transgen.com/antibody_tag/388.html<br>GFP antibody:https://www.transgen.com/antibody_tag/390.html<br>Ubiquitin antibody: https://www.abcam.com/en-us/products/primary-antibodies/ubiquitin-antibody-epr8830-bsa-and-azide-free-ab230145<br>Actin antibody:https://huabio.cn/products/Actin-antibody-ET1701-80<br>H3 antibody:https://huabio.cn/products/Histone-H3-antibody-ET1701-64<br>Anti-Mouse: https://www.abbkine.com/product/hrp-goat-anti-mouse-igg-a21010/<br>Anti-Rabbit: https://www.abbkine.com/product/hrp-goat-anti-rabbit-igg-a21020/

Dual use research of concern

Policy information about dual use research of concern

Hazards

Could the accidental, deliberate or reckless misuse of agents or technologies generated in the work, or the application of information presented in the manuscript, pose a threat to:

No	Yes	
[x]	[ ]	Public health
[x]	[ ]	National security
[x]	[ ]	Crops and/or livestock
[x]	[ ]	Ecosystems
[x]	[ ]	Any other significant area

Experiments of concern

Does the work involve any of these experiments of concern:

No	Yes	
☒	☐	Demonstrate how to render a vaccine ineffective
☒	☐	Confer resistance to therapeutically useful antibiotics or antiviral agents
☒	☐	Enhance the virulence of a pathogen or render a nonpathogen virulent
☒	☐	Increase transmissibility of a pathogen
☒	☐	Alter the host range of a pathogen
☒	☐	Enable evasion of diagnostic/detection modalities
☒	☐	Enable the weaponization of a biological agent or toxin
☒	☐	Any other potentially harmful combination of experiments and agents

Plants

Seed stocks	The seeds of TaIMP-α2 mutant α1AABBdd/α2aabbdd were collected from National Wheat Engineering Research Center, College of Agronomy, Henan Agricultural University; Others seeds which used in this study were stored in our lab.
Novel plant genotypes	We obtained the TaOST1B transgenic lines with its native promoter and the TaOST1B knockout lines in wheat.
Authentication	To screen the positive TaOST1B transgenic plants with its native promoter, the insertion of the expression vector into the genome was detected by PCR and the expression level of TaOST1B was detected by qRT-PCR.<br>To screen the positive the TaOST1B knockout lines, DNA fragment corresponding to the sgRNA-targeted DNA sequence regions was amplified from genomicDNA of knockout plants using corresponding primers and Hitom sequencing was carried out to examine the insertion or deletion at the target site.
