# Supplementary material for: The oligosaccharyltransferase TaOST1B promotes viral infection by enhancement of RNA silencing suppression in wheat
Source: Nat Commun. 2026 Jul 15;17:8688. doi: 10.1038/s41467-026-75397-8 (PMC13490409; doi:10.1038/s41467-026-75397-8)

nature portfolio

Peer Review File

The oligosaccharyltransferase TaOST1B promotes viral infection by enhancement of RNA silencing suppression in wheat

Corresponding Author: Professor Jian Yang

This file contains all reviewer reports in order by version, followed by all author rebuttals in order by version.

Version 0:

Reviewer comments:

Reviewer #1

(Remarks to the Author)
This study identified wheat oligosaccharyltransferase TaOST1B as a susceptibility gene for Wheat yellow mosaic virus (WYMV) through GWAS and QTL mapping. It negatively regulates wheat resistance to WYMV. TaOST1B^Hap1 interacts with WYMV's viral suppressor of RNA silencing (VSR) P1, mediates P1's N-glycosylation to promote P1's nuclear accumulation, maintains P1's RNAi-suppressing activity, and facilitates WYMV infection. In contrast, non-glycosylated P1 loses its VSR function, triggers endoplasmic reticulum (ER) stress, and is degraded by the 26S proteasome. Natural variants of TaOST1B cannot bind P1, leading to the same P1 degradation and reduced WYMV infection. Knocking out TaOST1B enhances WYMV resistance, while overexpressing TaOST1B^Hap1 increases susceptibility. The discovery that an oligosaccharyltransferase is hijacked by a viral VSR to promote plant viral infection reveals that N-glycosylation controls VSR function via nuclear localization and protein stability, linking ER stress and proteasomal degradation to antiviral defense. Key natural variations in TaOST1B provide a target for breeding WYMV-resistant wheat and shed light on plant-virus co-evolution. However, I still have several concerns.
1. The article establishes a model where a 2-amino acid deletion in natural TaOST1B variants (e.g., Hap2/Hap4) abolishes interaction with WYMV P1, but it fails to clarify the direct causal link between this deletion and impaired binding. It only infers a conformational change or binding site disruption without providing structural evidence (e.g., protein modeling, binding domain mapping) to explain how these specific residues (positions 18–19) mediate TaOST1B-P1 interaction. This creates a logical gap: the reader cannot confirm whether the deletion directly alters the P1-binding interface or indirectly affects protein folding, weakening the mechanistic coherence.
2. Additionally, the connection between P1's nuclear localization and RNAi suppression is somewhat underdeveloped. The article states that nuclear P1 blocks the CaM3-CAMTA3 interaction, but it does not explain why cytoplasmic P1 (e.g., non-glycosylated P1^N116A cannot interfere with this cytoplasmic interaction. This inconsistency in subcellular functional specificity is not resolved, leading to ambiguity in the model's logic.
3. The article identifies the 2B copy of TaOST1B as the dominant mediator of WYMV infection (via knockout of 2A/2B/2D copies), but it lacks experiments to validate why 2A and 2D copies are non-functional. For example: No tests were conducted to determine if TaOST1B-2A or TaOST1B-2D can interact with P1 or mediate P1 glycosylation (e.g., LCI, Co-IP, or LC-MS/MS for glycosylation intensity in 2A/2D knockout lines). The article only attributes non-functionality to unchanged expression post-WYMV infection, but does not rule out intrinsic functional defects (e.g., defective catalytic domains), leaving the "dominance of 2B" weakly supported.
4. The article claims non-glycosylated P1 is degraded via the 26S proteasome (using MG132, a proteasome inhibitor), but it does not exclude the involvement of autophagy, another major pathway for ER stress-induced protein degradation. No experiments (e.g., using autophagy inhibitors like wortmannin, or testing autophagy-related gene expression) were performed to confirm that the proteasome is the sole or primary degradation route. This omission weakens the evidence for the "26S proteasome-mediated degradation" claim.
5. The article concludes that TaOST1B^Hap2/Hap4 confer WYMV resistance based on reduced infection indices in field tests, but it does not address whether these variants have pleiotropic effects (e.g., on wheat growth, yield, or resistance to other pathogens). For example, oligosaccharyltransferases are involved in general protein glycosylation—disrupting their function might impair essential plant processes, but this is not evaluated.
6. The frequency of Hap2/Hap4 in global wheat germplasm. If these variants are rare, their utility for breeding is limited, but the article provides no data on their distribution, making the "breeding target" conclusion overly optimistic.

7. The article claims TaOST1B is the key mediator of P1 glycosylation, but it does not rule out the possibility of redundant glycosyltransferases in wheat. This makes the conclusion that "TaOST1B is essential for P1 glycosylation" less rigorous.
8. The discussion compares TaOST1B to other OST subunits (e.g., OsDGL1, TaSTT3b-2B) but fails to explore why WYMV specifically hijacks TaOST1B (rather than other OSTs) for P1 glycosylation. It also does not address whether TaOST1B's susceptibility function is a conserved trait in grasses or a wheat-specific adaptation, missing opportunities to link the findings to plant-virus co-evolutionary dynamics.
9. The discussion focuses narrowly on WYMV and P1, with no analysis of whether the "OST-mediated VSR glycosylation" mechanism applies to other plant viruses. For example, it does not discuss if VSRs from related viruses (e.g., barley yellow mosaic virus) rely on similar glycosylation pathways, which would contextualize the findings as a broader antiviral target or a WYMV-specific strategy.
10. The discussion does not acknowledge key gaps (e.g., the role of 2A/2D TaOST1B copies, alternative P1 degradation pathways) or propose future experiments to fill them. Instead, it emphasizes the study's contributions without critically evaluating its limitations.

Reviewer #2

(Remarks to the Author)
This study identifies TaOST1B, which encodes an oligosaccharyltransferase in wheat, through a combination of genome-wide association study (GWAS), biparental QTL mapping, and fine genetic mapping. The results demonstrate that TaOST1B acts as a susceptibility gene that promotes Wheat yellow mosaic virus (WYMV) infection. The authors conducted extensive molecular investigations to elucidate the mechanism by which TaOST1B facilitates viral infection. They showed that overexpression of TaOST1B enhanced WYMV accumulation, whereas CRISPR/Cas9-mediated knockout increased resistance, confirming its role as a susceptibility factor. Mechanistically, TaOST1B interacts with the viral silencing suppressor P1 and mediates its N-glycosylation at asparagine 116. This modification promotes the binding of P1 to the nuclear import protein TaIMP-α2, facilitating P1's nuclear accumulation. Nuclear-localized P1 maintains its viral suppressor of RNA silencing (VSR) activity, thereby enhancing WYMV infection. These findings were strongly supported by experiments using a non-glycosylatable P1 mutant ($P1^{N116A}$), which lost VSR activity, accumulated poorly in the nucleus, and failed to support efficient viral infection. In summary, through integrated genetic and molecular functional analyses, this study reveals a novel virulence strategy whereby WYMV hijacks the host glycosylation machinery to enhance viral infection. The identified susceptibility genes for WYMV infection, TaOST1B and TaIMP-α2, represent promising targets for future breeding of WYMV-resistant wheat varieties. Overall, I find this study is well-designed, but needs some revisions. Please find my comments below.
1. Some susceptibility genes have conserved housekeeping functions. Knocking them out can sometimes cause side effects that impair plant growth. In this study, both TaOST1B and TaIMP-α2 function as susceptibility genes for WYMV infection, which raises the question of whether knocking these two genes out causes any deleterious effects?
2. TaOST1B encodes an oligosaccharyltransferase. Does oligosaccharyltransferase catalyze only the N-glycosylation of substrates? Does TaOST1B catalyze N-glycosylation at residues other than asparagine 116 of P1? What type of N-glycans are attached through this N-glycosylation?

Lines 41 to 46: Please revise the sentences for better clarity, as not everyone knows that γb is one of the viral RNA strands of BSMV (Barley stripe mosaic virus).
Lines 31 to 50: It would be better to include abbreviations for the plant viruses where applicable, such as Chinese wheat mosaic virus (CWMV).
Lines 51 to 83: This manuscript studies the N-glycosylation of WYMV P1 and its consequences. I strongly recommend citing the recently published paper "An apoplastic fungal effector disrupts N-glycosylation of ZmLecRK1, inducing its degradation to suppress disease resistance in maize", which investigates the N-glycosylation of a maize receptor-like kinase by a fungal effector. Please also discuss it in the Discussion section.
Line 138: Change "the novel" to "the other".
Lines 142 to 145: The description is unclear, please rephrase.
Line 146: Change "locus" to "interval".
Line 147: The term "missense" is used in comparison to which line? This word is typically used to describe mutations compared to the wild type.
Line 150: "significantly upregulated post-viral infection in wheat" – in which wheat line?
Line 196: "NP-TaOST1BHap1" should be "NP-TaOST1BHap4".
Line 132: MG132 first appears here; more information about it should be provided at this point.
Line 322: How can LC-MS be used to identify P1-interacting proteins? Do you mean IP-MS (Immunoprecipitation-Mass Spectrometry)?
Fig. 1A: The recombination breakpoints should be positioned midway between the two flanking markers.
Fig. 1B and 1C: How were these graphs generated? There are no error bars in Fig. 1B, and the methods lack descriptions for these analyses.
Fig. 1G: A scale bar is needed to indicate gene size.
Fig. 4F: Please describe what the letters T, C, and N stand for.
Figure S4 is miswritten as Figure 4.

Reviewer #3

(Remarks to the Author)
The manuscript by Liu et al. reports a wheat oligosaccharyltransferase (TaOST1B) enhances WYMV infection through improving P1 N-glycosylation and stability. While, some natural variant of TaOST1B fails to bind P1 and regulate its N-glycosylation, which induces ER stress and proteasome-mediated degradation to attenuate WYMV infection. In summary, the study provides interesting genetic results showing the positive roles of TaOST1B in WYMV infection. However, the molecular mechanism is not convincing and need more evidence.
Major comments
1: As show in Figure 4K, the CAMAT3 and CaM3 interaction mainly occurs in the cytoplasm. How the nucleus-localized P1 or P1N116-NLS is required for the VSR activity through inhibiting the interaction of CAMAT3 and CaM3? Therefore, the real function of nucleus localization of N-glycosylated P1 remains elusive, probably in a independent way with CAMAT3 and CaM3.
2: The genetic results indicate that TaOST1B, rather than TaOST1A or TaOST1D, plays dominant roles in WYMV infections. The authors should demonstrate the difference between TaOST1B and TaOST1A/D, and explain the reason. Even more, it should be confirmed whether TaOST1A/D could interact with, glycosylate, and trigger the nuclear localization of P1?
3: As shown in Figure 6, N116A easily forms cytoplasmic bodies and induce ER stress, leading degradation via the 26S proteasome. These results are probably the real reason, rather than the result, for the effect of N116A and unglycosylation on virus infection. Thus, the nucleus localization of N-glycosylated P1 may be unrelated with the VSR and virus infection.
4: In Figure 2, all the genes should have at least two homozygous mutant lines to provide solid genetic results.

Minor Comments:
1: The title is confusing, because the main part of this work explain virus improvement by some variant of OST1B.
2: In the abstract, the full names of some abbreviations should be indicated, like TaMP-A2, CaM3, and CAMTA3.
3: In the final model, the specific VSR activities of P1 or P1N116A can be indicated.

Version 1:

Reviewer comments:

Reviewer #1

(Remarks to the Author)
I think the authors have addressed all my concerns.

Reviewer #2

(Remarks to the Author)
The authors have performed additional experiments and properly addressed all of my concerns. I have no further comments.

Reviewer #4

(Remarks to the Author)
This study demonstrates that the WYMV virus uses the glycosylation machinery of the plant host to prevent silencing of its RNA and promote infection. N-glycosylation of P1 enables nuclear localization, which appears to be crucial for the suppression of silencing. This is a highly interesting and groundbreaking story, as it links N-glycosylation in the endoplasmic reticulum (ER) to the so far unknown role of an N-glycan on the viral protein in the cytoplasm/nucleus. However, I have serious concerns about some of the data, and further experiments and explanations are needed to substantiate the findings.

1. The identified WYMV resistant and susceptible TaOST1B haplotypes differ in their 5'-coding regions. This region encodes the signal peptide of TaOST1B that is required for targeting to the ER. This is not mentioned by the authors, and it has important implications. Signal peptides are typically cleaved co-translationally, early during the translocation of the protein into the ER. So, how can the differences in the signal peptide sequence or its cleavage affect the interaction of TaOST1B with P1 that appears mediated by another TaOST1B protein domain? It should be analyzed by MS if the N-terminus of transiently expressed TaOST1B-Hap1 and TaOST1B-Hap4 differ in their N-terminal end.

2. The displayed protein-protein interactions are not entirely reliable due to their inherent limitations, which may result in the failure to detect genuine, biologically relevant interactions. The presence or absence of the signal peptide or individual amino acids may cause misfolding or poor expression, resulting in negative LCI results that are misinterpreted as a lack of interaction, when in fact they may be due to a lack of expression. I am concerned because the cLuc domain in the pCAMBIA1300-cLuc system is fused to the N-terminus of TaOST1B, masking the signal peptide so that it is neither recognized as a signal peptide nor cleaved. The same applies to the Y2H system, and both systems are out of their subcellular context, as TaOST1B is likely a membrane-anchored protein, with most of the protein in the ER lumen. The authors should carefully consider their data and provide additional controls or evidence. For example, show more co-IP data of plant-produced proteins like TaOST1B-HAP1 and TaOST1B-HAP4 with P1.

3. P1 is translated in the cytosol and a previous study (Sun et al., 2014 DOI: 10.1111/mpp.12109) has shown that it is associated with the ER membrane, presumably on the cytosolic side. The N-glycosylation by the oligosaccharyltransferase complex takes place in the lumen of the ER. So where is P1 localized in the infected plant cells and how is it targeted to the ER lumen for glycosylation? Does it have a signal peptide? Is it in the ER? The shown confocal images of P1 are not

entirely clear and differ from the images of Sun et al. 2014. Since it frequently happens that the fluorescent protein tags are cleaved off in the cytoplasm it is important to show relevant immunoblots of the intact fusion proteins without any quantitative amounts of free RFP/GFP that may label the cytosol and nucleus. The provided blots in the supplement lack the region below 40 KD.
Is P1-N11A-GFP in the ER as stated in line 292? This is not obvious from the shown images in S5C, where also the ER marker does not show a very clear ER labeling. Please provide better images. In addition, I would like to see also P1 co-expressed with the ER marker and the subcellular localization of both TaOST1B-Hap1 and TaOST1B-Hap4 with an ER marker.
The confocal data with H2B-RFP in Fig. 4C and 6D display also chloroplasts. For clarity it would be better to filter them out or at least mention that the nucleus and chloroplasts are visible in the images. Is H2B-RFP also present in the images in Fig. 6D, it appears so, but it is not indicated. Also, please mention that the RFP signal was used as a reference for the GFP signal.

4. Detection of the P1 glycosylation is essential for the overall conclusions. Yet the shown data raise some questions. Upon digestion with PNGases a shift in mobility is detectable for P1-PNGase or upon incubation with TM, but P1-N116A which lacks the N-glycosylation does not display a difference in mobility compared to P1. Do you have an explanation? To confirm the glycosylation of P1 from WYMV infected wheat, protein extracts from infected plants should be digested with PNGases and the shift in mobility should be shown with the P1 antibody as used in Fig. S5. Due to the lower molecular weight of P1 compared to P1-RFP the differences should be better visible. Moreover, also the N fraction in Fig. 5D should be digested with PNGases to confirm that glycosylated P1 is in the nucleus.

5. I did not understand how the intensity of glycopeptides for P1 was determined in Fig 3H. How was P1 purified from wheat? What does the graph show and how was it normalized? How much non-glycosylated peptide was identified compared to glycosylated P1 peptide? In my opinion it would be easier for the reader to provide relative amounts of glycosylated vs non-glycosylated peptide for every used line/condition. Also, the abundance data and spectra shown for the glycopeptides (Fig. 3F, 3G. 7C and Fig. S4) are not easy to understand. Please provide an additional table listing the relative amounts of all identified glycan structures.

6. In many of the shown immunoblot data there are quantitative data without any statistics. Yet, in the text, “significant” differences are frequently mentioned. Please add the relevant statistics, define the error bars and number of replications and how the bands were normalized. All this information is missing (e.g. for Fig 4E).

7. The degradation assay only showed small differences, and it is questionable whether any of the shown differences are statistically significant. How informative is the semi in vivo degradation assay? This assay was done using protein extracts. The used buffer is not clearly defined in the methods (e.g. which “protease” cocktail was used?) and likely includes a reducing agent and several protease inhibitors. While none of these will likely inhibit the proteasome, there is the question whether any conclusion can be made related to the involvement of autophagy. The inhibition of trafficking by wortmannin is not relevant in a non-cellular context and vacuolar proteases might be inhibited by the presence of the inhibitor cocktails and PMSF in the extraction buffer. Please comment on that and consider performing an in vivo assay with wheat protoplasts to provide clearer data on the involvement of the proteasome.

8. Absence of glycosylation may cause misfolding. It is suggested that nonglycosylated P1 may not fold correctly and thus trigger ER stress. However, attachment of an NLS and targeting of nonglycosylated P1 to the nucleus seems to restore the P1 suppressor function. Is this possible when nonglycosylated P1 is misfolded? Does P1-N116A-NLS trigger ER stress, is it degraded by the proteasome and does it interact with TaIMP-alpha2? The results of these experiments will provide valuable insights into the role of the P1 N-glycan.

9. Related to the model shown in Fig. 8. How is P1 transported back from the ER to the cytosol for localization in the nucleus? The presence of processed N-glycans instead of ER-characteristic mannosidic ones suggests that P1 is trafficking through the Golgi, this should be indicated in the illustration.

Version 2:

Reviewer comments:

Reviewer #4

(Remarks to the Author)
The revisions have improved the clarity and presentation of the data, and the authors have addressed most of my concerns. However, there are still some issues that need to be addressed.

Major issue:
Confirmation of the P1 glycosylation is essential for the overall conclusion. Yet the provided data and explanations are insufficient, and the MS data lack some clarity. The HILIC enrichment should be used for the MS-based identification of glycan structures linked to the P1 peptide. In addition to the MS/MS data, the full MS1 scans should be shown, all peaks related to glycopeptides should be annotated in the MS1 spectra and the relative intensities should be given in the Supplementary data.

The shown data for glycosylation intensity (Fig. 3F and 7D) are not informative and I would expect greater differences, for example, upon Tm treatment. This indicates a bias caused by the HILIC enrichment. To obtain more quantitatively balanced data on the amounts of non-glycosylated vs. glycosylated P1, the mixture of peptides and glycopeptides without HILIC enrichment needs to be compared. If the more highly abundant non-glycosylated peptides suppress the signal, then PNGase digestion of the glycopeptide is recommended. This will allow a more true relative comparison of glycosylated vs non-glycosylated P1. This data should be shown instead of the data shown in Fig. 3F and 7D.

Minor points:
In the text (e.g. lines 302, 305, 315, 371, 460, 464) significant is used to describe the differences. However, none of the shown data contains a statistic showing that the differences are indeed significant. Please change the text accordingly or provide the necessary information and statistic tests.
The figure legends still lack some relevant information. For example, the shown variations (standard deviation?) are not defined (e.g. Fig. 4G, 6B, 6D, 7G, S2E, S5D,S6A, S6B, S7F, S12E).
Which wheat variety was used for the wheat protoplasts experiments or purification (Fig. S3E).
In Figure 6D the 2h value for P1-GFP is 1.28, this is likely not correct.
Please describe more precisely how the P1 protein was purified (line 796).
Please define the used protease inhibitor cocktail (line 860, line 882).
The semi-in vivo degradation assay still mentions the use of wortmannin (line 884).
In Figure S12, refer to panel “D” in the legend. Define the marker bands in D.

Version 3:

Reviewer comments:

Reviewer #4

(Remarks to the Author)
I appreciate the authors’ efforts to revise the manuscript and respond to prior concerns. However, the experimental issues related to the amount of glycosylated versus non-glycosylated P1 remain insufficiently addressed.

The provided data still do not show the relative proportion (%) of glycosylated P1 for each treatment and plant line used. It is also unclear why the reported relative intensity ratio of glycosylated to non-glycosylated P1 is 1. If this were the case, it would imply that 50% of total P1 is glycosylated and 50% is non-glycosylated, which appears unlikely.

The glycosylation site occupancy (%) should instead be calculated as the intensity of the PNGase F digestion–derived deamidated peptide divided by the sum of the intensities of the deamidated peptide and the corresponding non-glycosylated (unmodified) peptide.

I assume that these calculations are already included in the additional datasets; however, these data should also be presented clearly in the main body of the manuscript.

Open Access This Peer Review File is licensed under a Creative Commons Attribution 4.0 International License, which permits use, sharing, adaptation, distribution and reproduction in any medium or format, as long as you give appropriate credit to the original author(s) and the source, provide a link to the Creative Commons license, and indicate if changes were made.
In cases where reviewers are anonymous, credit should be given to 'Anonymous Referee' and the source.
The images or other third party material in this Peer Review File are included in the article's Creative Commons license, unless indicated otherwise in a credit line to the material. If material is not included in the article's Creative Commons license and your intended use is not permitted by statutory regulation or exceeds the permitted use, you will need to obtain permission directly from the copyright holder.
To view a copy of this license, visit https://creativecommons.org/licenses/by/4.0/

We sincerely thank the reviewers for their constructive suggestions to further improve the manuscript. In the following, we respond point-by-point to all the comments and questions raised by the reviewers and indicate corresponding changes made in the revised manuscript.

Reviewer #1 (Remarks to the Author)

This study identified wheat oligosaccharyltransferase TaOST1B as a susceptibility gene for Wheat yellow mosaic virus (WYMV) through GWAS and QTL mapping. It negatively regulates wheat resistance to WYMV. TaOST1B^{Hap1} interacts with WYMV's viral suppressor of RNA silencing (VSR) P1, mediates P1's N-glycosylation to promote P1's nuclear accumulation, maintains P1's RNAi-suppressing activity, and facilitates WYMV infection. In contrast, non-glycosylated P1 loses its VSR function, triggers endoplasmic reticulum (ER) stress, and is degraded by the 26S proteasome. Natural variants of TaOST1B cannot bind P1, leading to the same P1 degradation and reduced WYMV infection. Knocking out TaOST1B enhances WYMV resistance, while overexpressing TaOST1B^{Hap1} increases susceptibility. The discovery that an oligosaccharyltransferase is hijacked by a viral VSR to promote plant viral infection reveals that N-glycosylation controls VSR function via nuclear localization and protein stability, linking ER stress and proteasomal degradation to antiviral defense. Key natural variations in TaOST1B provide a target for breeding WYMV-resistant wheat and shed light on plant-virus co-evolution. However, I still have several concerns.

1. The article establishes a model where a 2-amino acid deletion in natural TaOST1B variants (e.g., Hap2/Hap4) abolishes interaction with WYMV P1, but it fails to clarify the direct causal link between this deletion and impaired binding. It only infers a conformational change or binding site disruption without providing structural evidence (e.g., protein modeling, binding domain mapping) to explain how these specific residues (positions 18-19) mediate TaOST1B-P1 interaction. This creates a logical gap: the reader cannot confirm whether the deletion directly alters the P1-binding interface or indirectly affects protein folding, weakening the mechanistic coherence.

Response: Thank you for your comment. First, we analyzed the conserved domain of TaOST1B and found that it mainly consists of a core Ribophorin_I domain (Figure A below). We initially examined the interactions between P1 and the Ribophorin_I domains of TaOST1B^{Hap1} or TaOST1B^{Hap4}. The LCI assays showed that both Ribophorin_I domains of TaOST1B^{Hap1} and TaOST1B^{Hap4} could interact with

P1 (Figure B below). However, when the corresponding N-terminal sequences were added to the Ribophorin_I, respectively. Hap1 could still interact with P1, while the interaction of Hap4 with P1 was lost. Further, we made a deletion mutation of the 18-19 amino acids in Hap1 and found that the mutant also lost its interaction with P1 (Figure B below), which suggested that 18-19 amino acids are important for the binding of TaOST1B to P1. Additionally, through protein model simulation, we discovered that the 2-amino acid deletion caused a change in the N-terminal helical structure of TaOST1B (Figure C below). Therefore, we speculated that this 2-amino acid deletion likely impairs binding through an allosteric effect on the protein's overall folding. These findings have been supplemented in lines 424-435 and Fig. 7 of revised manuscript.

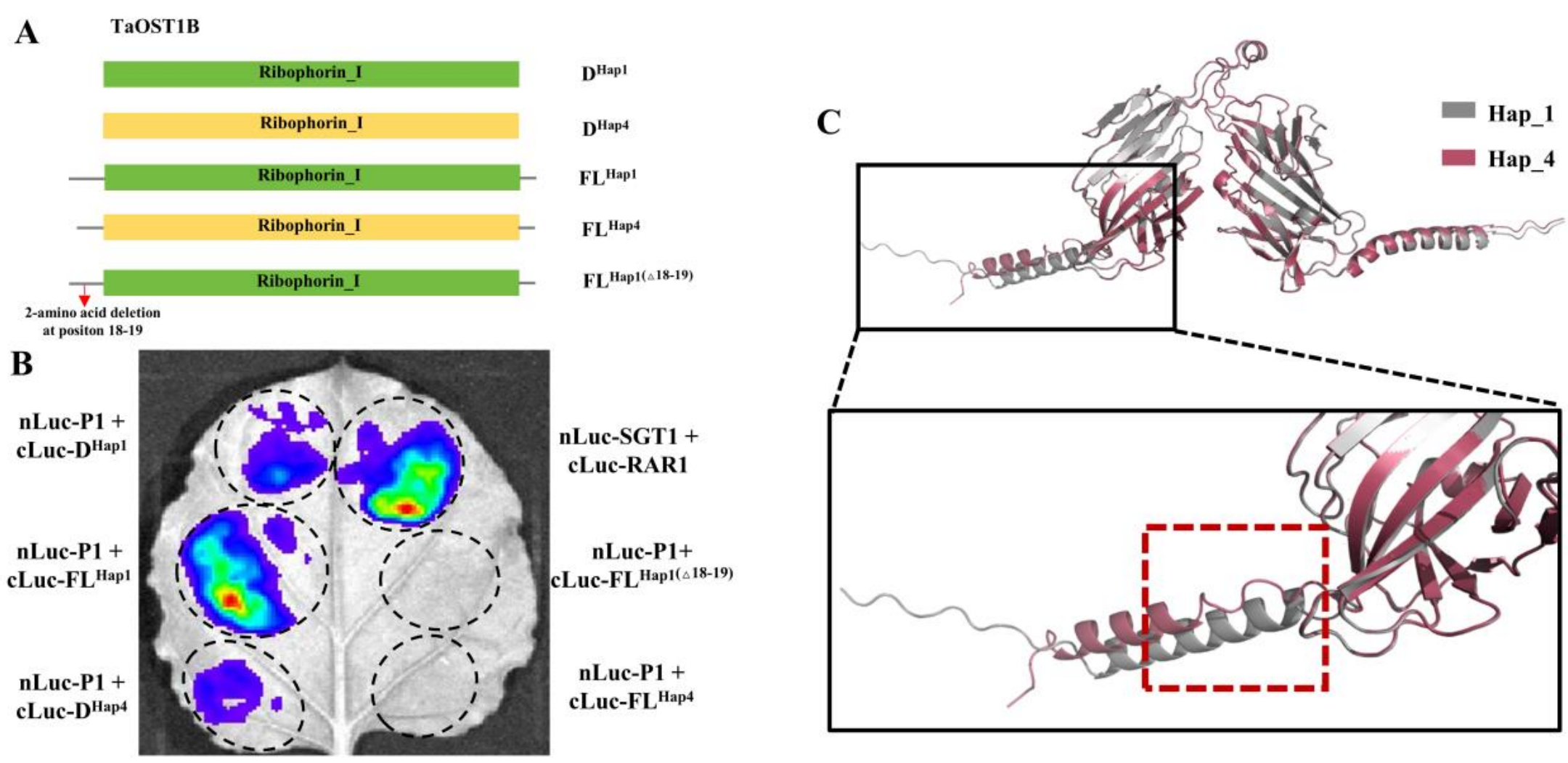

2. Additionally, the connection between P1's nuclear localization and RNAi suppression is somewhat underdeveloped. The article states that nuclear P1 blocks the CaM3-CAMTA3 interaction, but it does not explain why cytoplasmic P1 (e.g., non-glycosylated $P1^{N116A}$ cannot interfere with this cytoplasmic interaction. This inconsistency in subcellular functional specificity is not resolved, leading to ambiguity in the model's logic.

Response: Thank you for your comment. Our interaction analysis revealed that CaM3-CaMTA3 interaction was detectable in both the cytoplasm and nucleus (Figure below). The new interaction analysis found that P1 could bind to CaMTA3 in the nucleus, while non-glycosylated $P1^{N116A}$ failed to interact with CaMTA3 (Figure A, B below). Upon fusion with a nuclear localization signal (NLS), $P1^{N116A}$-NLS regained the ability to bind CaMTA3 in the nucleus. In contrast, appending a nuclear

export signal to P1 (P1-NES) abolished its interaction with CaMTA3 (Figure A, B below). These results demonstrate that nuclear localization of P1 is required for its interaction with CaMTA3. We further found that nuclear-localized P1 and P1^{N116A}-NLS disrupted the CaMTA3-CaM3 interaction participating in the RNAi pathway, non-glycosylated P1^{N116A} didn't (Figure C below, Fig. 4F, 4G). The cytoplasmic P1 does not interact with CaMTA3, so it cannot disrupt the CaMTA3-CaM3 interaction. These findings indicate that the nuclear import of P1, even though non-glycosylation, could block the interaction CaMTA3 and CaM3. These data have been supplemented in lines 328-341 and Fig. 4 of revised manuscript.

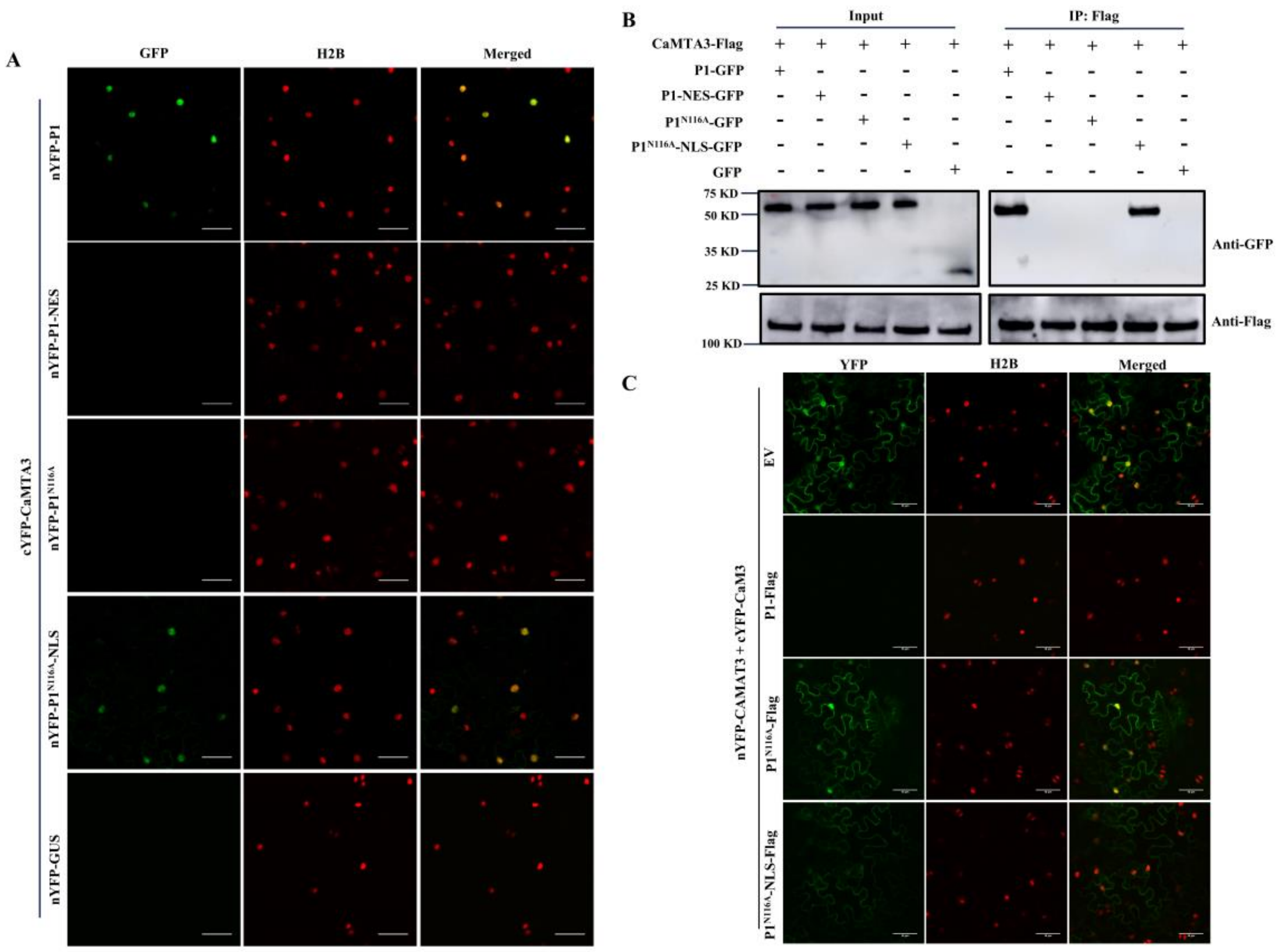

3. The article identifies the 2B copy of TaOST1B as the dominant mediator of WYMV infection (via knockout of 2A/2B/2D copies), but it lacks experiments to validate why 2A and 2D copies are non-functional. For example: No tests were conducted to determine if TaOST1B-2A or TaOST1B-2D can interact with P1 or mediate P1 glycosylation (e.g., LCI, Co-IP, or LC-MS/MS for glycosylation intensity in 2A/2D knockout lines). The article only attributes non-functionality to unchanged expression post-WYMV infection, but does not rule out intrinsic functional defects (e.g., defective catalytic domains), leaving the "dominance of 2B" weakly supported.

Response: Thank you for your comment. The gene expression results showed that after WYMV infection, the expression of *TaOST1B*-2B significantly increased, while there were no obvious changes in *TaOST1B*-2A and *TaOST1B*-2D (Figure A below). Furthermore, LCI assays demonstrated that neither TaOST1B-2A nor TaOST1B-2D interacts with P1 (Figure B below). We detected the N-glycosylation intensity of P1 in WYMV-infected *TaOST1B*-2A, *TaOST1B*-2B, *TaOST1B*-2D knockout lines and wild-type Fielder plants. The results showed no significant difference in N-glycosylation intensity of P1 among *TaOST1B*-2A, *TaOST1B*-2D knockout lines and wild-type plants (Figure C below). Therefore, the low expression of *TaOST1B*-2A and *TaOST1B*-2D and the loss of their binding ability to P1 may result in their non-functionality for WYMV infection. These data have been supplemented in lines 231-233, 266-268 and Fig. 3 and Fig. S3 of revised manuscript.

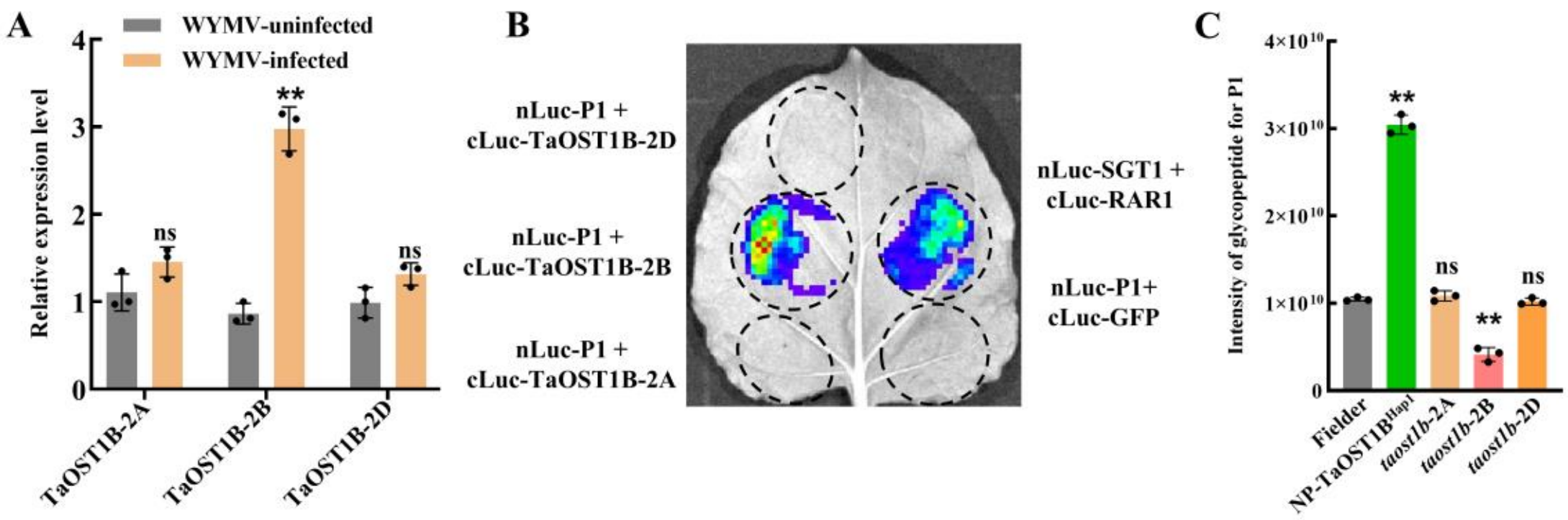

4. The article claims non-glycosylated P1 is degraded via the 26S proteasome (using MG132, a proteasome inhibitor), but it does not exclude the involvement of autophagy, another major pathway for ER stress-induced protein degradation. No experiments (e.g., using autophagy inhibitors like wortmannin, or testing autophagy-related gene expression) were performed to confirm that the proteasome is the sole or primary degradation route. This omission weakens the evidence for the "26S proteasome-mediated degradation" claim.

Response: Thank you for your comment. In the first submission of our manuscript, we employed the the proteasome inhibitor MG132 and autophagy inhibitor wortmannin to investigate the degradation pathways of P1 and non-glycosylated P1^{N116A}. Compared with those in the DMSO-treated samples, non-glycosylated P1^{N116A} protein accumulation levels were clearly increased in the MG132-treated samples at the same time point, wortmannin-treated non-glycosylated P1^{N116A} showed no significant accumulation changes latterly compared with the control (Figure below). This demonstrates that non-glycosylated P1 is primarily degraded through the 26S proteasome. The results of the wortmannin

treatment were previously shown in the supplemental figure, we have adjusted it to Fig. 6 in revised manuscript.

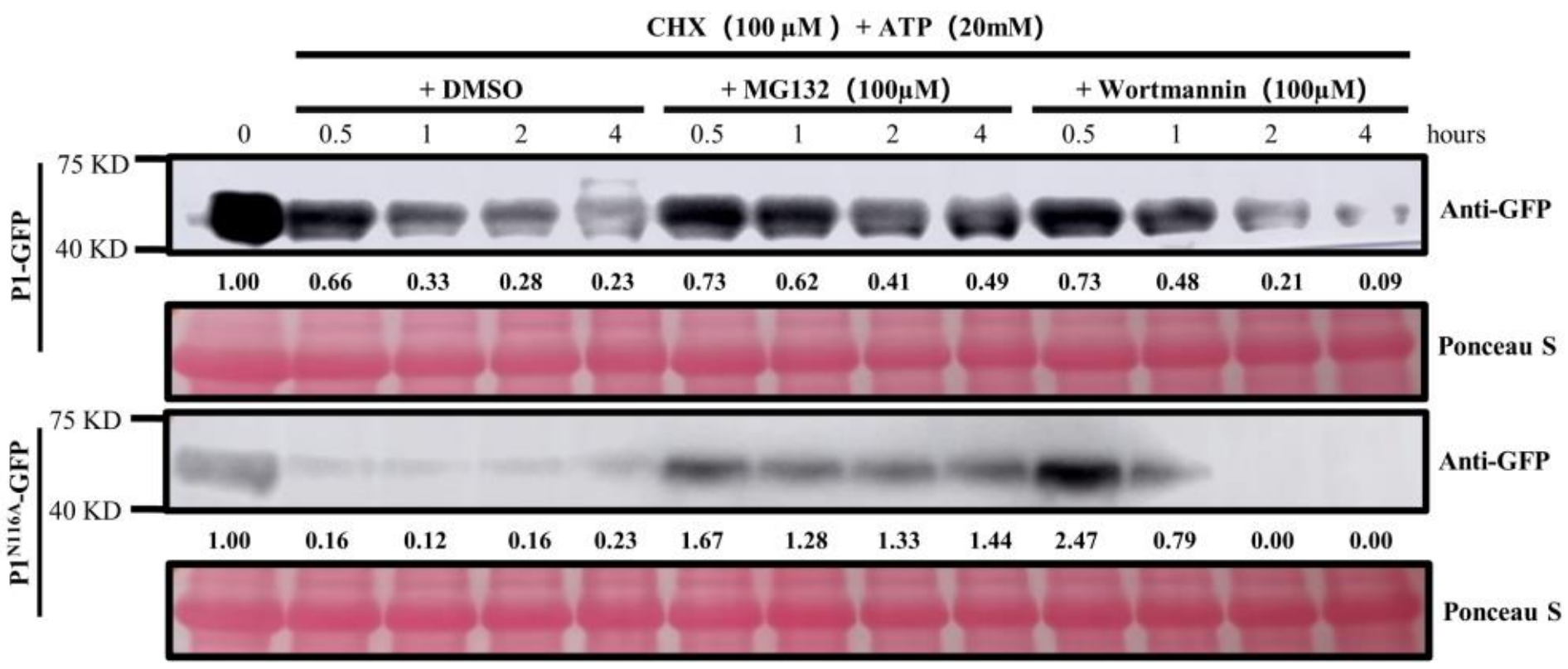

5. The article concludes that TaOST1B$^{Hap2/Hap4}$ confer WYMV resistance based on reduced infection indices in field tests, but it does not address whether these variants have pleiotropic effects (e.g., on wheat growth, yield, or resistance to other pathogens). For example, oligosaccharyltransferases are involved in general protein glycosylation—disrupting their function might impair essential plant processes, but this is not evaluated.

Response: Thank you for the important comment. We measured some agronomic traits of wheat plants corresponding the four haploid at maturity, including plant height, tiller number, panicle length, spekelets number per panicle, seed size and thousand-grain weight. We found that there were no significant differences among them (Figure below). Furthermore, we also evaluated the seedling growth of *TaOST1B* knockout lines in controlled environments and yield-related traits at maturity in the field. The results showed that the triple mutant lines *taost1b* exhibited significantly slower seedling emergence compared to wild-type and single mutant lines. Additionally, plant height and spikelets per panicle were markedly reduced in *taost1b* lines relative to wild-type and single mutants, but tiller number was significantly higher in *taost1b*, *taost1b*-2B, and *taost1b*-2A lines compared to wild-type and *taost1b*-2D lines. However, no notable differences were observed in panicle length, seed size, and thousand-grain weight among these lines (Figure below). The effects of these growth processes were predominantly observed in the triple mutant, but not in the single-copy knockout lines. This demonstrates that there is significant functional redundancy among the TaOST1B three homoeologs for wheat plant growth. These data have been supplemented in lines 500-514 and Fig. S8 and S9.

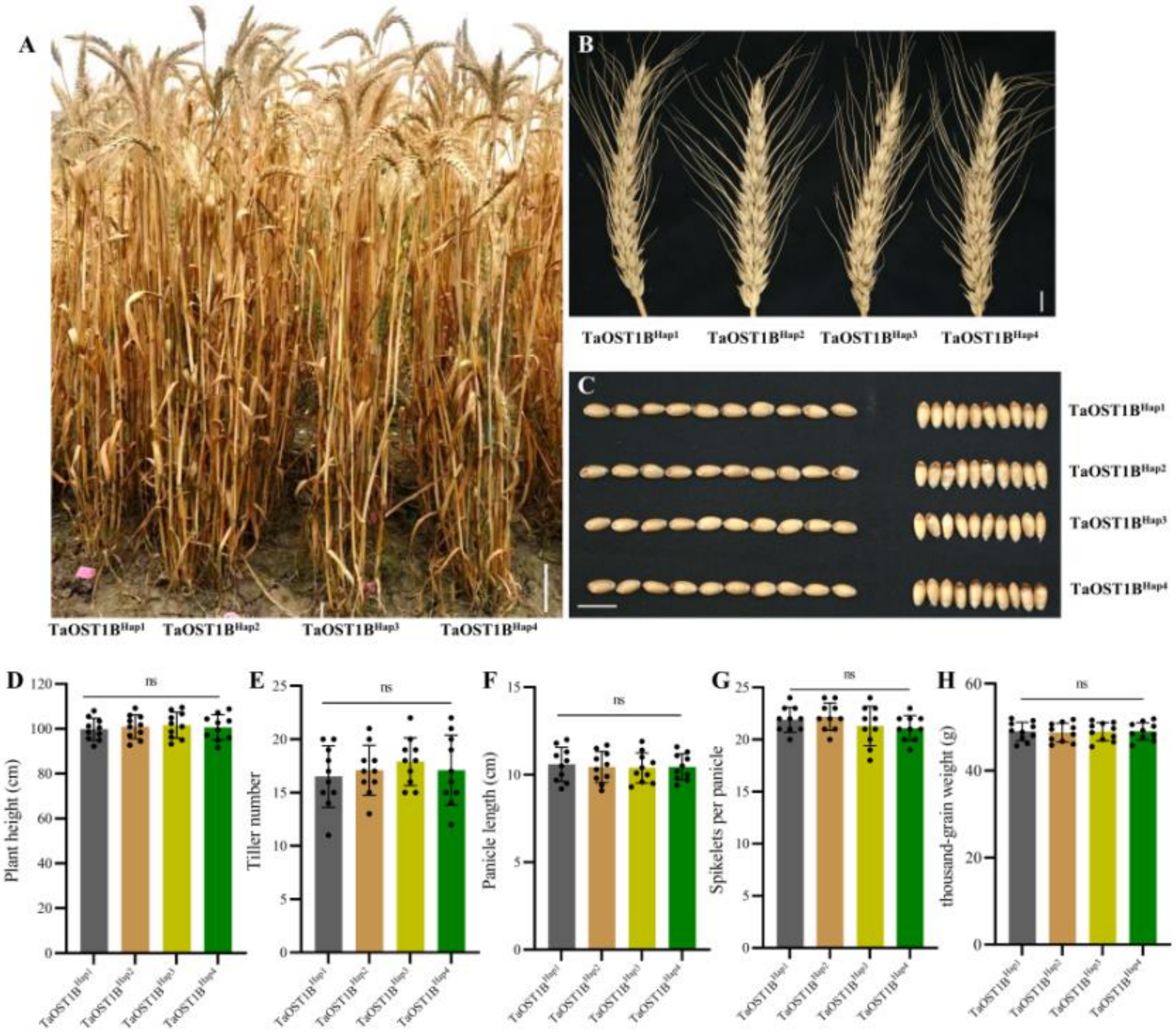

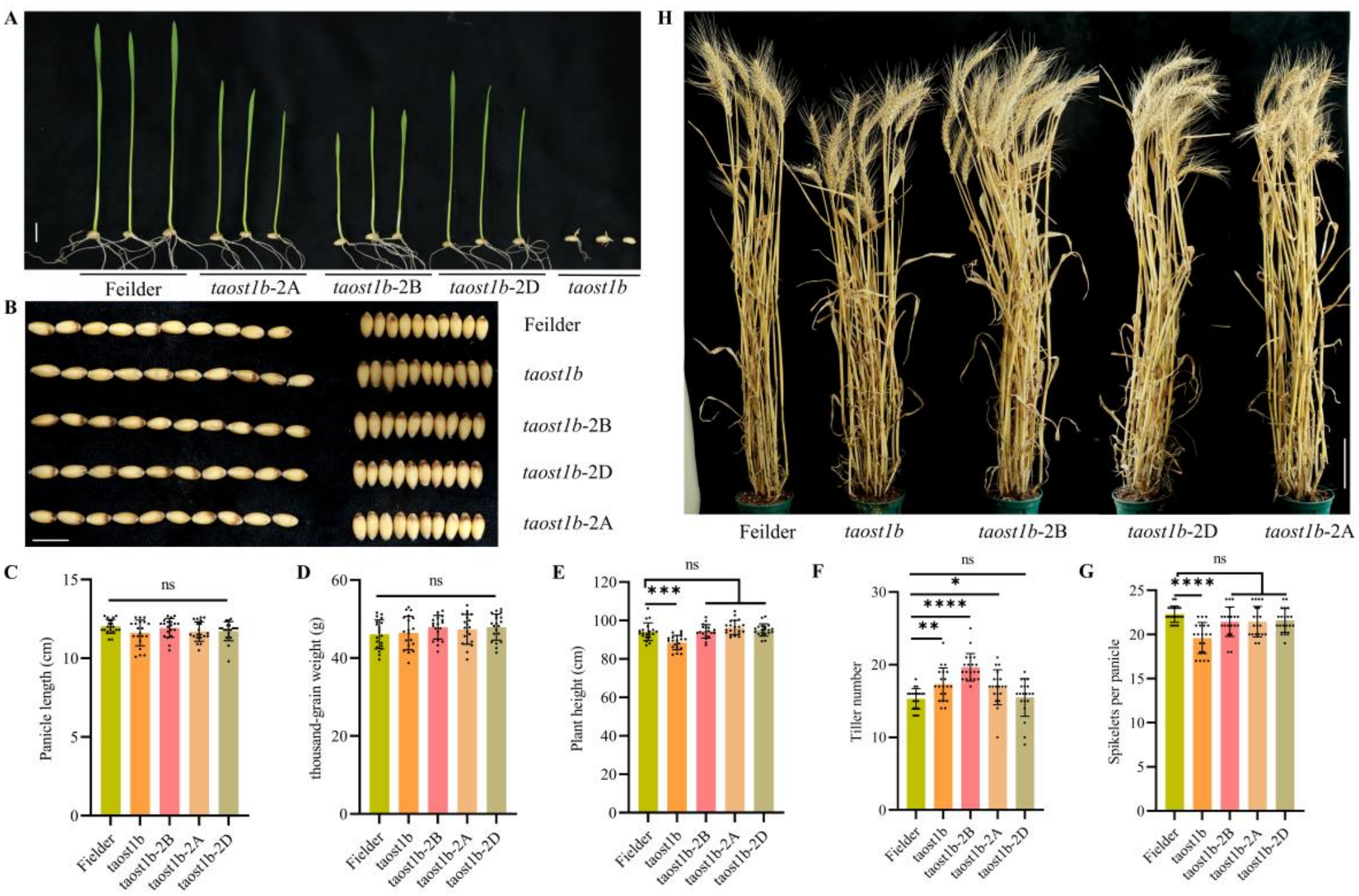

6. The frequency of Hap2/Hap4 in global wheat germplasm. If these variants are rare, their utility for breeding is limited, but the article provides no data on their distribution, making the "breeding target" conclusion overly optimistic.

Response: Thank you for your insightful comment. We found that the frequency of Hap2/Hap4 is only ~4% in our natural populations and wheat varieties of public databases. We speculated that this natural

variation is likely rare in global wheat germplasm. In response, we have toned down our conclusions throughout the text, removing the overly optimistic statement about being a direct "breeding target". Since Hap2/Hap4 represent valuable haplotypes enhancing WYMV resistance, their identification enables their use as potential targets in future wheat improvement programs. We have supplemented these in lines 514-516 of the revised manuscript.

7. The article claims TaOST1B is the key mediator of P1 glycosylation, but it does not rule out the possibility of redundant glycosyltransferases in wheat. This makes the conclusion that "TaOST1B is essential for P1 glycosylation" less rigorous.

Response: Thank you for your comment. We found that $TaOST1B^{Hap1}$ can promote WYMV infection through GWAS, fine mapping, expression pattern analysis, genetic validation. Further biochemical experiments revealed that TaOST1B is required for P1 N-glycosylation. In response, we have toned down our statements throughout the manuscript, and we have replaced "TaOST1B is essential for P1 glycosylation" with "TaOST1B is required for P1 glycosylation" in lines 267-268 of revised manuscript.

8. The discussion compares TaOST1B to other OST subunits (e.g., OsDGL1, TaSTT3b-2B) but fails to explore why WYMV specifically hijacks TaOST1B (rather than other OSTs) for P1 glycosylation. It also does not address whether TaOST1B's susceptibility function is a conserved trait in grasses or a wheat-specific adaptation, missing opportunities to link the findings to plant-virus co-evolutionary dynamics.

Response: Thank you for your constructive comments. The OST complex includes different subunits, which are responsible for substrate recognition, catalysis, and structural stability. OsDGL1 and OST3/6 are intrinsically required for the plant growth and development. TaSTT3b-2B is specifically expressed after being attacked by *Rhizoctonia cerealis*, it resists pathogen infection by regulating the expression of a series of defense-related genes. In our study, TaOST1B was specifically induced by WYMV and positively regulated WYMV infection in wheat. Studies have reported that the OST1 can recognize nascent polypeptides that are destined to be glycosylated in yeast (Yan et al., 1999; Bai et al., 2018). RNA viruses are obligate intracellular infectious pathogens, they usually rely on recruiting certain host factors to facilitate own reproduction. TaOST1B is required for successful WYMV infection by interacting with the viral-encoded P1, likely recognition its nascent peptide to enhance P1 N-glycosylation. This modification activates P1 function as viral suppressor of RNA silencing (VSR),

thereby promoting viral infection. However, the potential roles of other OST subunits in the P1 N-glycosylation process will also be a major focus in our future research. In response, we have revised the discussion section in lines 459-485 of the manuscript.

Additionally, we performed a Blast analysis against the EnsemblPlants database to identify orthologs of TaOST1B in grasses. We found that orthologs with amino acid sequence identities exceeding 80% were found mainly in cereal species, and all of them contain the conserved Ribophorin_I domain of the oligosaccharyltransferase (Figure below). Based on this, we speculate that TaOST1B is likely a susceptibility protein that evolved under the specific selective pressure of cereal-infecting viruses. We have supplemented discussion in lines 516-521 and Fig. S10 of revised manuscript.

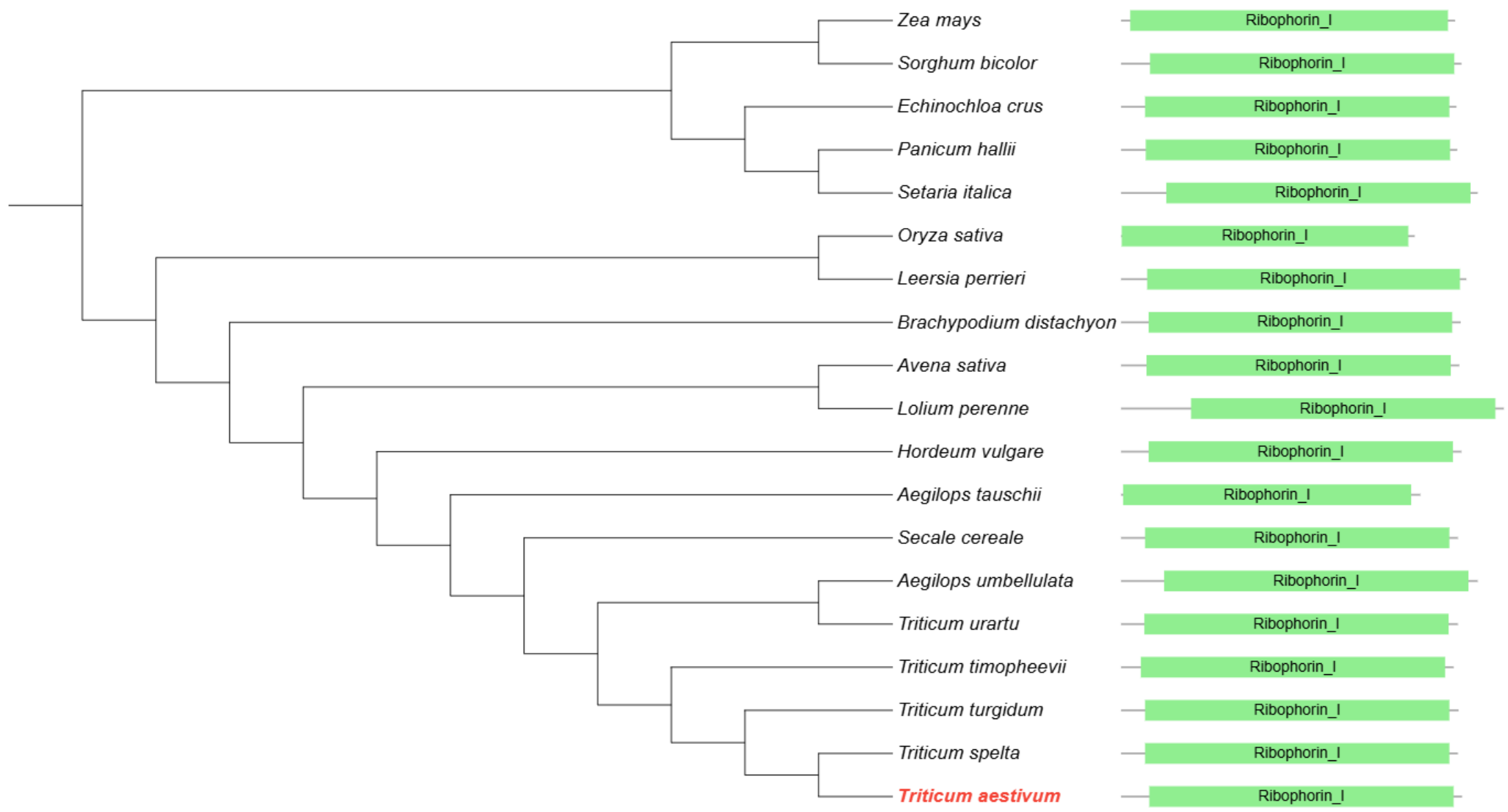

9. The discussion focuses narrowly on WYMV and P1, with no analysis of whether the "OST-mediated VSR glycosylation" mechanism applies to other plant viruses. For example, it does not discuss if VSRs from related viruses (e.g., barley yellow mosaic virus) rely on similar glycosylation pathways, which would contextualize the findings as a broader antiviral target or a WYMV-specific strategy.

Response: Thank you for your comment. We used Wheat spindle streak mosaic virus (WSSMV), Barley yellow mosaic virus (BaYMV), Barley mild mosaic virus (BaMMV), and Oat mosaic virus (OMV) as key examples, given their close phylogenetic relationship to WYMV. Through online website NetNGlyc 1.0 server, we predicted that the VSRs encoded by these viruses all contained potential N-glycosylation sites. Sequence alignment revealed that the asparagine residue at position 116 of P1 is highly conserved in the homologous proteins encoded by these viruses (Figure A below). We

therefore hypothesize that their VSRs might also be subjected to similar OST-mediated glycosylation. Additionally, we have preliminarily tested the function of TaOST1B in relation to the Chinese wheat mosaic virus (CWMV), which belongs to *Virgavirdae*, *Furovirus*, could co-infect with WYMV. We found that TaOST1B also enhanced CWMV infection in wheat (Figure B-D below), which suggested that TaOST1B-mediated glycosylation could be utilized by CWMV. Therefore, we propose that most plant viruses require host OST-mediated N-glycosylation to facilitate successful viral infection, which might make the host OST a new broad-spectrum antiviral target. We have supplemented discussion in lines 521-534 and Fig. S10 of revised manuscript.

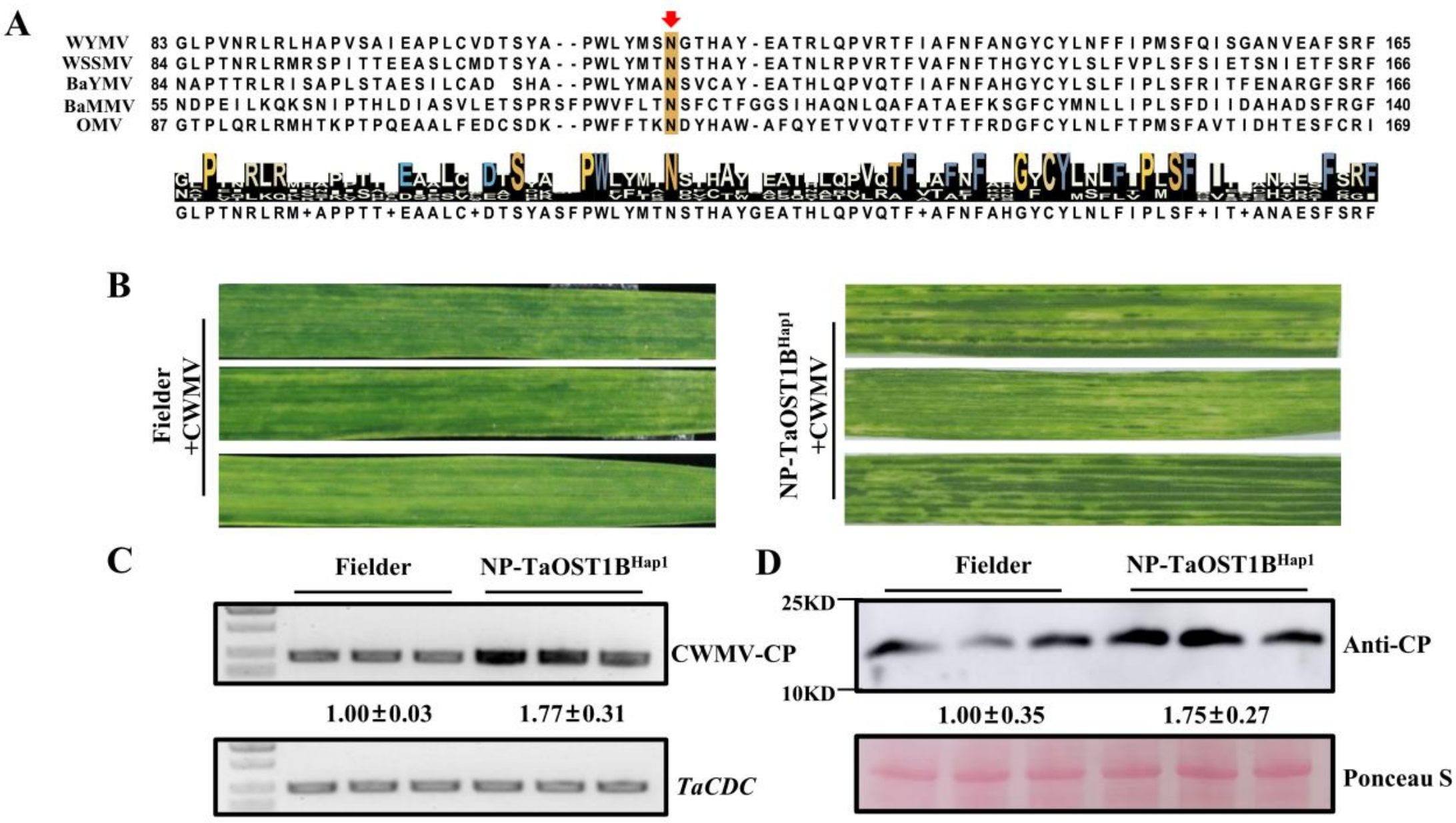

10. The discussion does not acknowledge key gaps (e.g., the role of 2A/2D TaOST1B copies, alternative P1 degradation pathways) or propose future experiments to fill them. Instead, it emphasizes the study's contributions without critically evaluating its limitations.

Response: Thank you for your constructive comment. In response, we have expanded the Discussion section to address key remaining questions and outline future research directions. Our data show that TaOST1B-2B is required for P1 N-glycosylation and successful WYMV infection. While simultaneous knockout of all three TaOST1B copies enhanced WYMV resistance, it also led to significantly delayed seedling emergence and reduced plant height at maturity. In contrast, single knockout of the 2B copy alone increased viral resistance without obvious growth defects, whereas single mutants of the 2A or 2D copy neither affected WYMV infection nor plant growth. These results suggest functional redundancy among the three TaOST1B homeologs in wheat development. Specifically, TaOST1B-2A

and TaOST1B-2D may partially compensate for the loss of TaOST1B-2B in wheat growth processes, although their precise regulatory pathways and molecular mechanisms require further investigation.

Underglycosylation commonly results in protein misfolding and aggregate accumulation within the ER lumen, triggering ER stress. Eukaryotic cells alleviate this stress through ER-associated degradation (ERAD). The E3 ligase hydroxymethylglutaryl reductase 1 (HRD1) a core component of the ERAD pathway, can interact with the TGB1-3 protein encoded by beet necrotic yellow vein virus and ubiquitinate TGB1-3, thereby facilitating its degradation through the 26S proteasome to inhibit viral infection (Guo et al., 2025). Our study showed that non-glycosylated P1 displayed obvious aggregation in cells and triggered ER stress, it has stronger ubiquitination and faster 26S proteasome-mediated degradation rates than N-glycosylated P1. These indicate that P1 could evade the host's ubiquitination and subsequent degradation by undergoing N-glycosylation. However, how glycosylation and ubiquitination coordinately regulate WYMV infection in wheat, as well as the host factors and regulatory mechanisms underlying the ubiquitination of non-glycosylated P1 protein, remain key directions for our future research.

Limitations of the study: TaOST1B is an oligosaccharyltransferase subunit that mediates N-glycosylation of P1. However, the precise molecular mechanism underlying its recognition of the P1 nascent peptide remains unclear. Future studies should employ structural analyses to elucidate their binding mechanism. Additionally, the WYMV resistance-enhanced haplotypes TaOST1B^{Hap2} and TaOST1B^{Hap4} are rare in wheat germplasms, which may constrain their direct breeding utility. Nevertheless, they represent promising targets for wheat improvement through strategies such as marker-assisted selection and gene editing.

These points have now been incorporated into the revised manuscript in lines 498-512, 587-600, and 613-620 of the Discussion section.

Reviewer #2 (Remarks to the Author)

This study identifies TaOST1B, which encodes an oligosaccharyltransferase in wheat, through a combination of genome-wide association study (GWAS), biparental QTL mapping, and fine genetic mapping. The results demonstrate that TaOST1B acts as a susceptibility gene that promotes Wheat yellow mosaic virus (WYMV) infection. The authors conducted extensive molecular investigations to elucidate the mechanism by which TaOST1B facilitates viral infection. They showed that

overexpression of TaOST1B enhanced WYMV accumulation, whereas CRISPR/Cas9-mediated knockout increased resistance, confirming its role as a susceptibility factor. Mechanistically, TaOST1B interacts with the viral silencing suppressor P1 and mediates its N-glycosylation at asparagine 116. This modification promotes the binding of P1 to the nuclear import protein TaIMP-α2, facilitating P1's nuclear accumulation. Nuclear-localized P1 maintains its viral suppressor of RNA silencing (VSR) activity, thereby enhancing WYMV infection. These findings were strongly supported by experiments using a non-glycosylatable P1 mutant ($P1^{N116A}$), which lost VSR activity, accumulated poorly in the nucleus, and failed to support efficient viral infection. In summary, through integrated genetic and molecular functional analyses, this study reveals a novel virulence strategy whereby WYMV hijacks the host glycosylation machinery to enhance viral infection. The identified susceptibility genes for WYMV infection, TaOST1B and TaIMP-α2, represent promising targets for future breeding of WYMV-resistant wheat varieties. Overall, I find this study is well-designed, but needs some revisions. Please find my comments below.

1. Some susceptibility genes have conserved housekeeping functions. Knocking them out can sometimes cause side effects that impair plant growth. In this study, both TaOST1B and TaIMP-α2 function as susceptibility genes for WYMV infection, which raises the question of whether knocking these two genes out causes any deleterious effects?

Response: Thank you very much for your question. We measured some agronomic traits of TaIMP-α2 knockout lines and the wild-type Fielder at maturity, including plant height, tiller number, panicle length, spekelets number per panicle, seed size and thousand-grain weight. We found that there were no significant differences between KO-TaIMP-α2 and Fielder (Figure below). We focused on evaluating the seedling growth of TaOST1B knockout lines in controlled environments and yield-related traits at maturity in the field. The results showed that the triple mutant lines *taost1b* exhibited significantly slower seedling emergence compared to wild-type and single mutant lines. Additionally, plant height and spikelets per panicle were markedly reduced in taost1b lines relative to wild-type and single mutants, but tiller number was significantly higher in *taost1b*, *taost1b*-2B, and *taost1b*-2A lines compared to wild-type and *taost1b*-2D lines. However, no notable differences were observed in panicle length or thousand-grain weight among these lines (Figure below). The effects of these growth processes were predominantly observed in the triple mutant, but not in the single-copy knockout lines. This demonstrates that there is significant functional redundancy among the TaOST1B three

homoeologs for wheat plant growth. These data have been supplemented in lines 500-512 and Fig. S8.

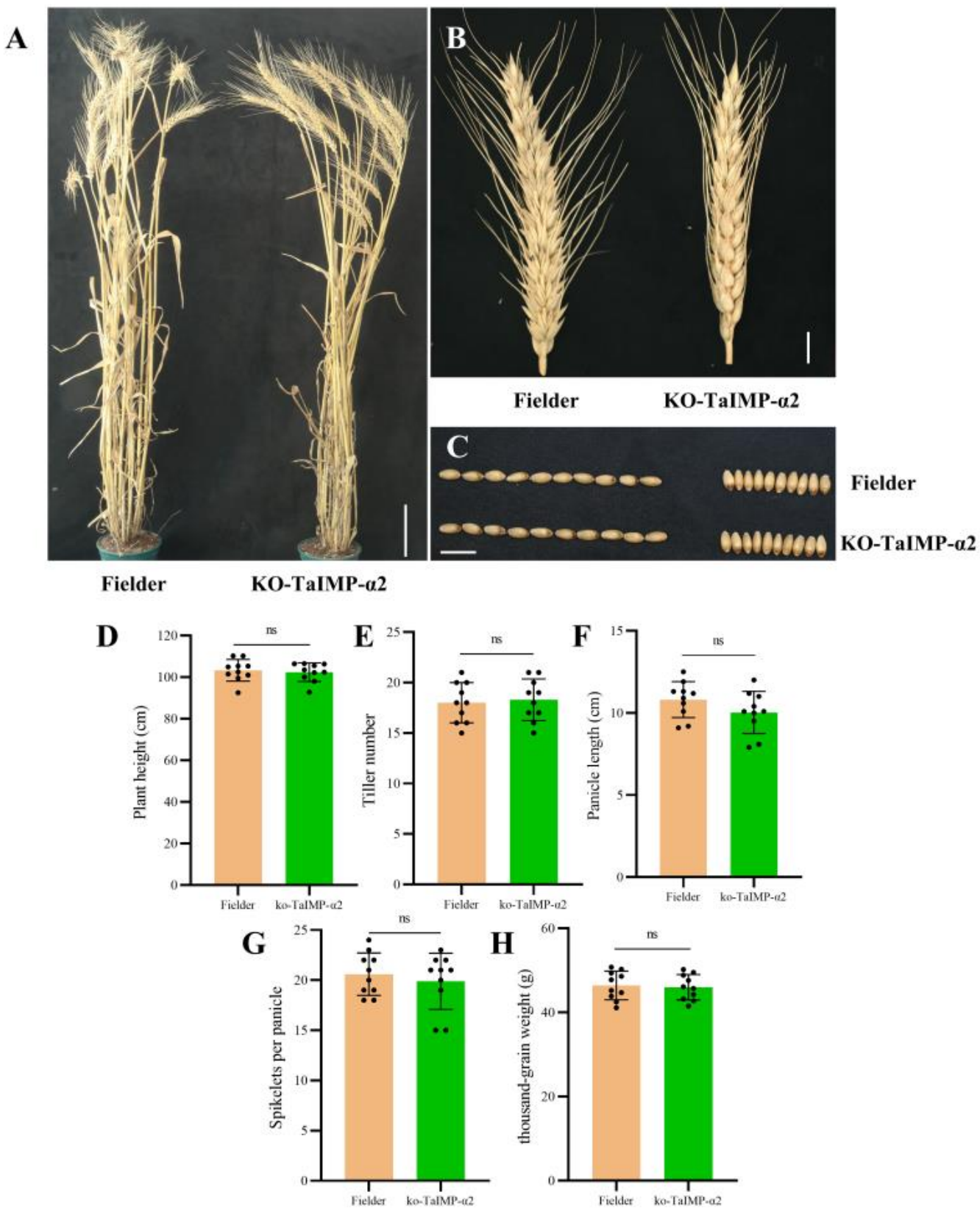

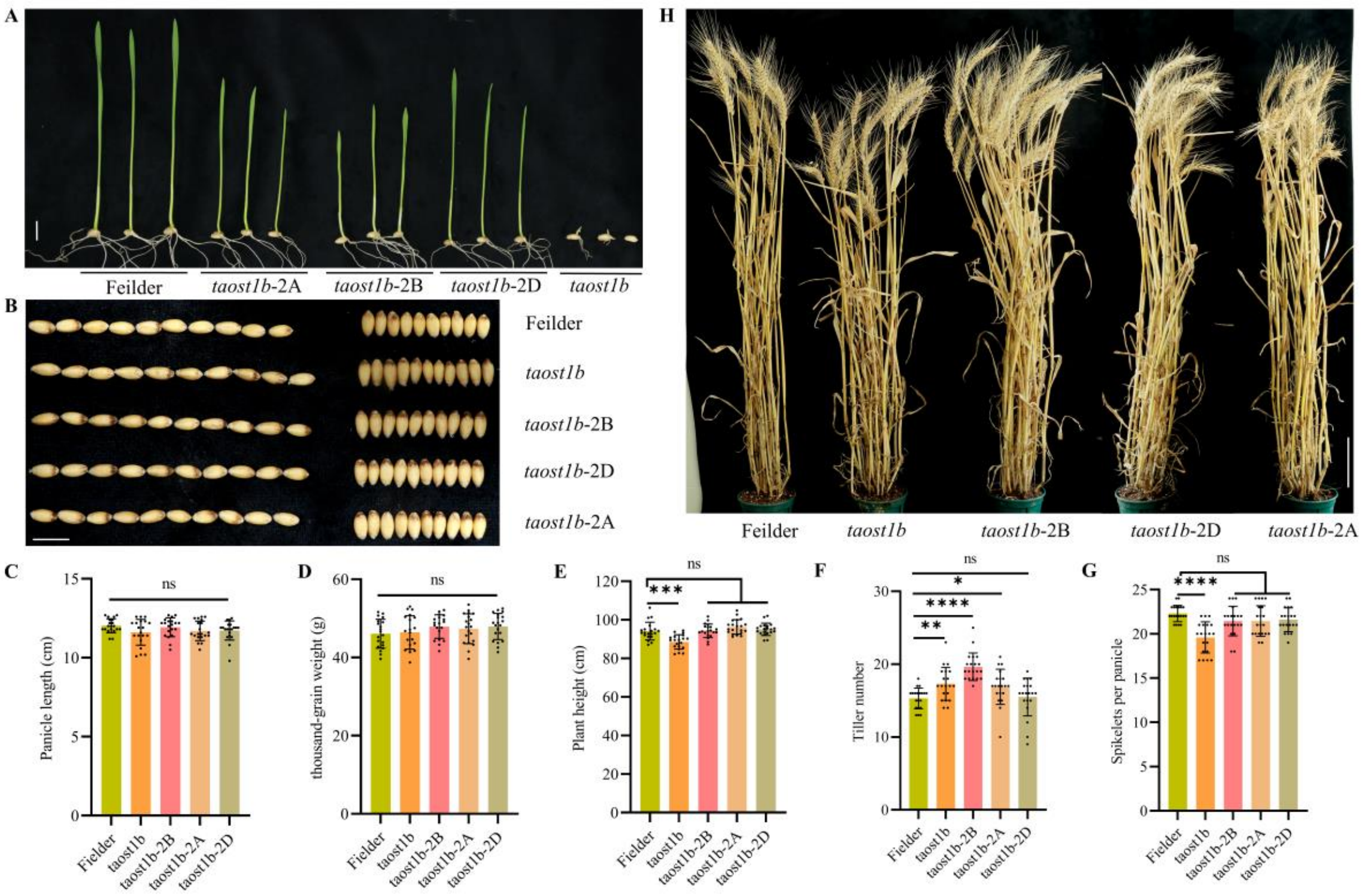

2. TaOST1B encodes an oligosaccharyltransferase. Does oligosaccharyltransferase catalyze only the N-glycosylation of substrates? Does TaOST1B catalyze N-glycosylation at residues other than

asparagine 116 of P1? What type of N-glycans are attached through this N-glycosylation?

Response: Thank you for your important questions. It is well-established that the primary and canonical function of oligosaccharyltransferase (OST) is to catalyze the transfer of the core oligosaccharide from a dolichol pyrophosphate-linked donor to an asparagine (Asn) residue within the Asn-X-Ser/Thr (X not Pro) motif of nascent polypeptides. In research, we found that WYMV-encoded P1 specifically interacted with TaOST1B to regulate its N-glycosylation, and multiple rounds of mass spectrometry determined that Asn-116 is the sole N-glycosylated residue in the P1 protein (Fig. 3G). The representative higher-energy collisional dissociation fragmentation spectra of the N-glycopeptides of P1 showed that the attached N-glycans are primarily paucimannose and complex/hybrid chains containing fucose and xylose (Fig. 3G, S4B, S7B).

3. Lines 41 to 46: Please revise the sentences for better clarity, as not everyone knows that γb is one of the viral RNA strands of BSMV (Barley stripe mosaic virus).

Response: Thank you for your comment. We have revised these sentences.

4. Lines 31 to 50: It would be better to include abbreviations for the plant viruses where applicable, such as Chinese wheat mosaic virus (CWMV).

Response: Thank you for your comment. We have added abbreviations for the plant viruses where applicable.

5. Lines 51 to 83: This manuscript studies the N-glycosylation of WYMV P1 and its consequences. I strongly recommend citing the recently published paper "An apoplastic fungal effector disrupts N-glycosylation of ZmLecRK1, inducing its degradation to suppress disease resistance in maize", which investigates the N-glycosylation of a maize receptor-like kinase by a fungal effector. Please also discuss it in the Discussion section.

Response: Thank you for your comment. We have cited this latest research in lines 81-84 of the revised manuscript and discussed it in line 495-498.

6. Line 138: Change "the novel" to "the other".

Response: Thank you for your comment. We have changed "the novel" to "the other".

7. Lines 142 to 145: The description is unclear, please rephrase.

Response: Thank you for your comment. We have rephrased these sentences.

8. Line 146: Change "locus" to "interval".

Response: Thank you for your comment. We have changed "locus" to "interval".

9. Line 147: The term "missense" is used in comparison to which line? This word is typically used to describe mutations compared to the wild type.

Response: Thank you for your correct and important observation. We performed a comparative analysis of the gene sequences between parental lines. To enhance precision, we have replaced the term "missense" with "non-synonymous" in our description.

10. Line 150: "significantly upregulated post-viral infection in wheat" – in which wheat line?

Response: Thank you for your question. We analyzed the expression patterns of these genes in the susceptible line PI610750 both before and after infection with WYMV. We have incorporated this information into the revised manuscript.

11. Line 196: "NP-TaOST1B^{Hap1}" should be "NP-TaOST1B^{Hap4}".

Response: Thank you for your comment. We have corrected "NP-TaOST1B^{Hap1}" to "NP-TaOST1B^{Hap4}".

12. Line 312: MG132 first appears here; more information about it should be provided at this point.

Response: Thank you for your comment. We have supplemented related information of MG132 at first appear.

13. Line 322: How can LC-MS be used to identify P1-interacting proteins? Do you mean IP-MS (Immunoprecipitation-Mass Spectrometry)?

Response: Thank you for your comment. This is my mistake. It should be Immunoprecipitation-Mass Spectrometry (IP-MS). We have corrected that.

14. Fig. 1A: The recombination breakpoints should be positioned midway between the two flanking markers.

Response: Thank you for your comment. We have adjusted the position of the recombination breakpoints in Fig. 1A.

15. Fig. 1B and 1C: How were these graphs generated? There are no error bars in Fig. 1B, and the methods lack descriptions for these analyses.

Response: Thank you for pointing out these omissions. These graphs were generated using GraphPad Prism 9.0. The data for Fig. 1B was obtained from the Hexaploid Wheat Gene Expression Database (Chinese Spring Development) in WheatOmics 1.0, while the data for Fig. 1C was derived from qRT-PCR analysis. We have provided a detailed description in updated the figure legends accordingly.

16. Fig. 1G: A scale bar is needed to indicate gene size.

Response: Thank you for your comment. We have added the scale bar to indicate gene size in Fig. 1G.

17. Fig. 4F: Please describe what the letters T, C, and N stand for.

Response: Thank you for your comment. We have supplemented the means of these letters in figure legends.

18. Figure S4 is miswritten as Figure 4.

Response: Thank you for your comment. We have corrected it.

Reviewer #3 (Remarks to the Author)

The manuscript by Liu et al. reports a wheat oligosaccharyltransferase (TaOST1B) enhances WYMV infection through improving P1 N-glycosylation and stability. While, some natural variant of TaOST1B fails to bind P1 and regulate its N-glycosylation, which induces ER stress and proteasome-mediated degradation to attenuate WYMV infection. In summary, the study provides interesting genetic results showing the positive roles of TaOST1B in WYMV infection. However, the molecular mechanism is not convincing and need more evidence.

1. As show in Figure 4K, the CAMAT3 and CaM3 interaction mainly occurs in the cytoplasm. How the nucleus-localized P1 or P1^{N116A}-NLS is required for the VSR activity through inhibiting the interaction of CAMAT3 and CaM3? Therefore, the real function of nucleus localization of N-glycosylated P1 remains elusive, probably in a independent way with CAMAT3 and CaM3.

Response: Thank you very much for your question. In our initial submission, the effect of P1 on the CaMTA3-CaM3 interaction was examined in wild-type *N. benthamiana* plants, where their interaction was detectable in both the cytoplasm and nucleus. To clarify this finding, we repeated the experiment using a transgenic *N. benthamiana* line stably expressing the nuclear marker H2B-RFP. This result confirmed that the CaMTA3-CaM3 interaction signal in nucleus overlapped with the H2B-RFP, offering more precise localization than our initial data. In addition, The new interaction analysis found that P1 could bind to CaMTA3 in the nucleus, while non-glycosylated P1^{N116A} failed to interact with CaMTA3 (Figure A, B below). Upon fusion with a nuclear localization signal (NLS), P1^{N116A}-NLS regained the ability to bind CaMTA3 in the nucleus. In contrast, appending a nuclear export signal to P1 (P1-NES) abolished its interaction with CaMTA3 (Figure A, B below). These results demonstrate that nuclear localization of P1 is required for its interaction with CaMTA3. While N-glycosylation acts

as a specific signal that promotes nuclear trafficking of P1. We further found that nuclear-localized P1 and P1^{N116A}-NLS disrupted the CaMTA3-CaM3 interaction participating in the RNAi pathway to act as VSR function, cytoplasmic P1^{N116A} didn't (Figure C below). This indicates that the nuclear import of P1, even though non-glycosylation, could block the CaMTA3-CaM3 interaction. These data have been supplemented in lines 328-341 and Fig. 4 of revised manuscript.

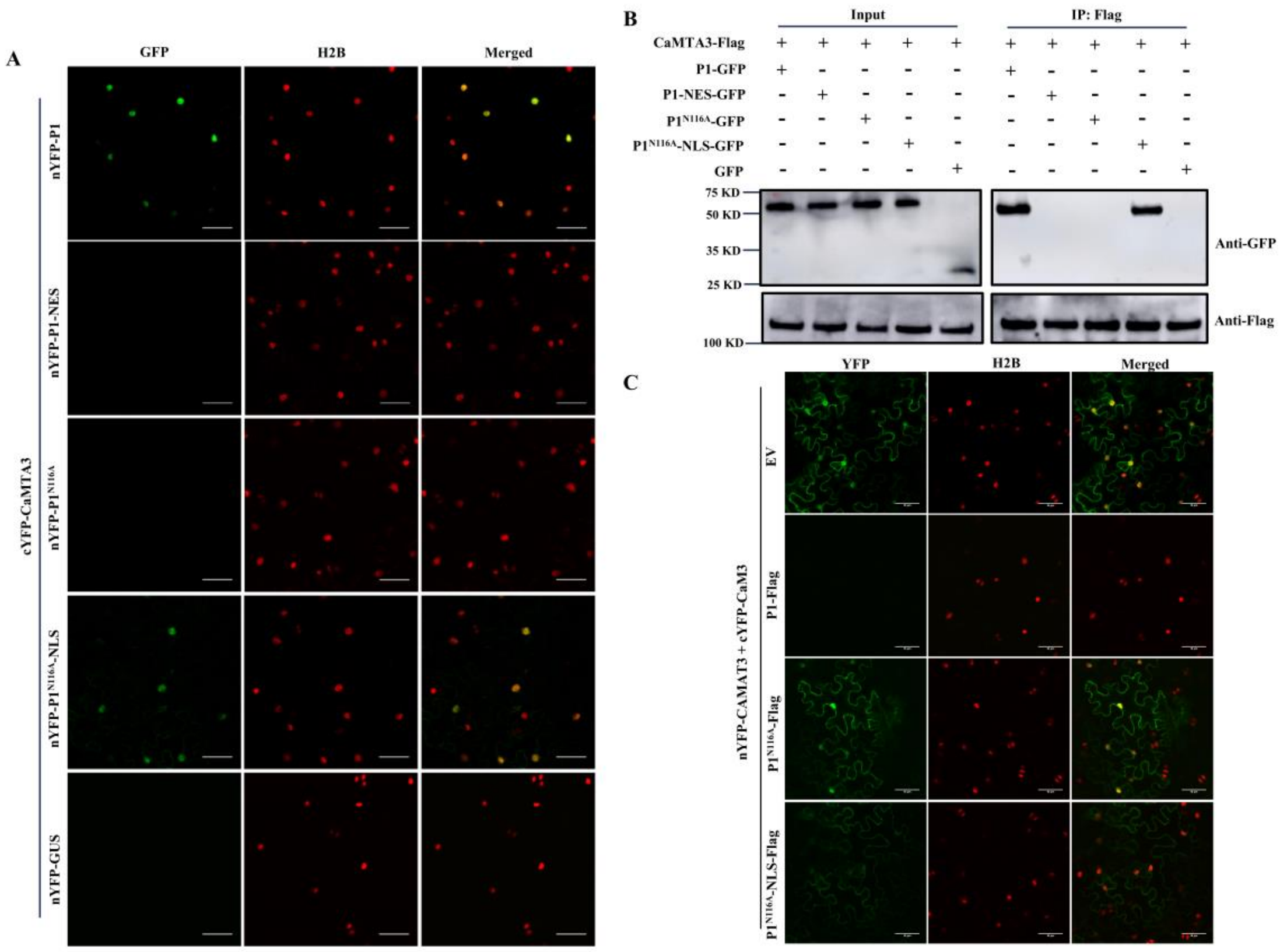

2. The genetic results indicate that TaOST1B, rather than TaOST1A or TaOST1D, plays dominant roles in WYMV infections. The authors should demonstrate the difference between TaOST1B and TaOST1A/D, and explain the reason. Even more, it should be confirmed whether TaOST1A/D could interact with, glycosylate, and trigger the nuclear localization of P1?

Response: Thank you for your critical question. We first performed LCI assay, the results demonstrated that neither TaOST1B-2A nor TaOST1B-2D interacts with P1 (Figure A below). Furthermore, we detected the N-glycosylation intensity of P1 in WYMV-infected *TaOST1B*-2A, *TaOST1B*-2D knockout lines and wild-type plants. The results showed no significant difference in N-glycosylation intensity of P1 among *TaOST1B*-2A, *TaOST1B*-2D knockout lines and wild-type plants (Figure B below). We further examined the nucleo-cytoplasmic distribution of the P1 in WYMV-infected TaOST1B-2A/2D knockout lines and wild-type plants. The results showed no

obvious difference in the nuclear accumulation levels of P1 among *TaOST1B*-2A, *TaOST1B*-2D knockout lines and wild-type plants (Figure C below). These findings indicate that *TaOST1B*-2A and *TaOST1B*-2D are non-functional in the N-glycosylation and nuclear distribution of P1. Moreover, the gene expression results showed that after WYMV infection, the expression of *TaOST1B*-2B significantly increased, while there were no obvious changes in *TaOST1*B-2A and *TaOST1B*-2D (Fig. S2F). Therefore, their non-functionality could be due to the low expression and the loss of their binding ability to P1. We have included these data in lines 231-233, 265-268, 302-304 and Fig. 3, S3, and S5 of revised manuscript.

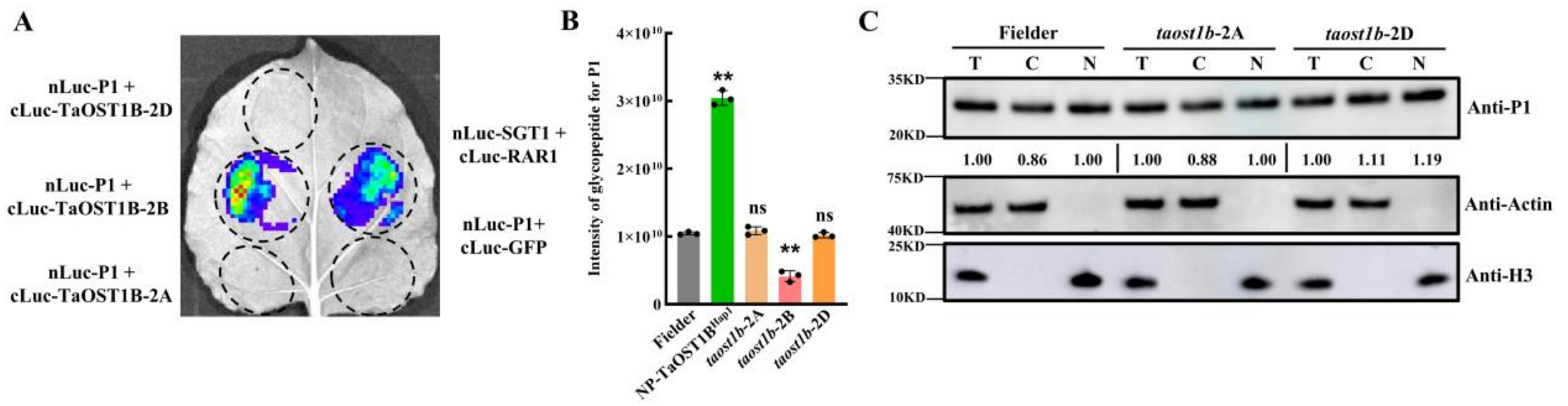

3. As shown in Figure 6, N116A easily forms cytoplasmic bodies and induce ER stress, leading degradation via the 26S proteasome. These results are probably the real reason, rather than the result, for the effect of N116A and unglycosylation on virus infection. Thus, the nucleus localization of N-glycosylated P1 may be unrelated with the VSR and virus infection.

Response: Thank you for your comment. Our data support a causal pathway: The absence of N-glycosylation in P1 prevents its proper nuclear import, which causes P1 to misfold and form aggregates in the cytoplasm (Fig. 4C, Fig. 5D), and subsequently induces ER stress and degradation through 26S proteasome (Fig. 6). Simultaneously, the lack of nuclear P1 impairs its ability to interact with CaMTA3 and execute its VSR function. By forcibly directing the non-glycosylated P1 to the nucleus ($P1^{N116A}$-NLS), we observed: A significant recovery of its VSR activity and viral infectivity (Fig. 4E, 4F, 4G); Restoration of its interaction with CaMTA3 (Fig. 4H). This indicates that the nuclear import of P1, even non-glycosylation, could block the CaMTA3-CaM3 interaction. The cytoplasmic aggregation and degradation of $P1^{N116A}$ are consequences of its non-glycosylation, which in turn affects the WYMV infection. In summary, N-glycosylation serves as a specific signal for P1 nuclear trafficking, its loss traps P1 forming aggregates in the cytoplasm, leading to protein instability and functional impairment. We have revised the discussion in lines 605-607.

4. In Figure 2, all the genes should have at least two homozygous mutant lines to provide solid genetic results.

Response: Thank you for your suggestion. We have included data from two independent homozygous mutant lines for each gene. Due to missing the window for natural field infection, these lines and wild-type plants were inoculated with WYMV in chamber under controlled conditions. Subsequent phenotyping and viral detection were then carried out to assess the level of resistance. All independent mutant lines consistently recapitulate the similar phenotype and accumulation level of WYMV-CP as the lines originally presented. We have included these data in Fig. S2 of revised manuscript.

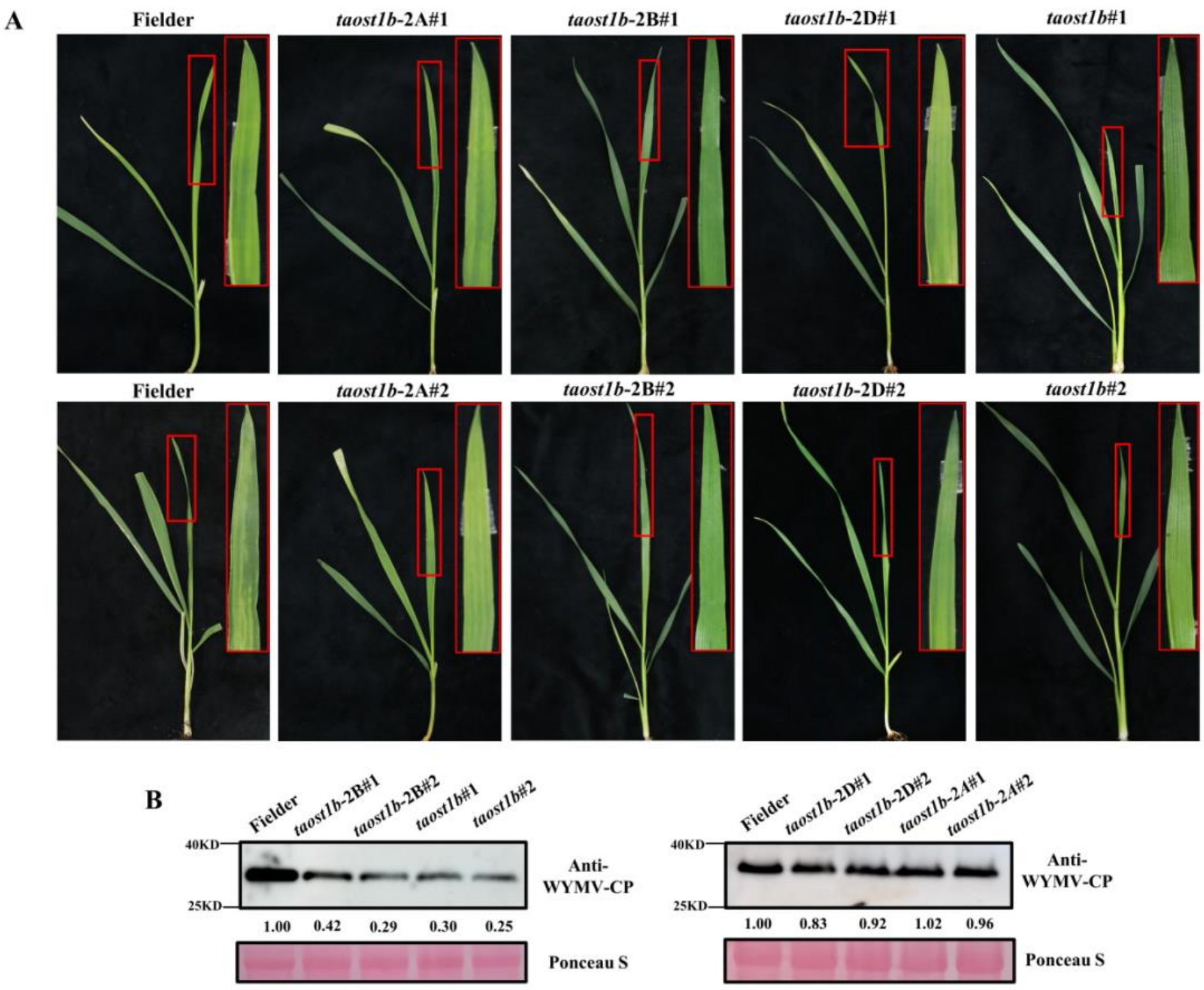

5. The title is confusing, because the main part of this work explain virus improvement by some variant of OST1B.

Response: Thank you for your comment. We have adjusted the title of the article to "The oligosaccharyltransferase TaOST1B promotes viral infection by enhancement of RNA silencing suppression in wheat".

6. In the abstract, the full names of some abbreviations should be indicated, like TaMP-A2, CaM3, and CaMTA3.

Response: Thank you for your comment. We have included details of these abbreviations.

7. In the final model, the specific VSR activities of P1 or P1^{N116A} can be indicated.

Response: Thank you for your comment. We have adjusted the final model.

We sincerely thank the reviewers for their constructive suggestions to further improve the manuscript. In the following, we respond point-by-point to all the comments and questions raised by the reviewers and indicate corresponding changes made in the revised manuscript.

Reviewer #4 (Remarks to the Author):

This study demonstrates that the WYMV virus uses the glycosylation machinery of the plant host to prevent silencing of its RNA and promote infection. N-glycosylation of P1 enables nuclear localization, which appears to be crucial for the suppression of silencing. This is a highly interesting and groundbreaking story, as it links N-glycosylation in the endoplasmic reticulum (ER) to the so far unknown role of an N-glycan on the viral protein in the cytoplasm/nucleus. However, I have serious concerns about some of the data, and further experiments and explanations are needed to substantiate the findings.

1. The identified WYMV resistant and susceptible TaOST1B haplotypes differ in their 5'-coding regions. This region encodes the signal peptide of TaOST1B that is required for targeting to the ER. This is not mentioned by the authors, and it has important implications. Signal peptides are typically cleaved co-translationally, early during the translocation of the protein into the ER. So, how can the differences in the signal peptide sequence or its cleavage affect the interaction of TaOST1B with P1 that appears mediated by another TaOST1B protein domain? It should be analyzed by MS if the N-terminus of transiently expressed TaOST1B-Hap1 and TaOST1B-Hap4 differ in their N-terminal end.

Response: Thank you for your comment. To directly address this issue, we performed LC-MS/MS analysis of immunoprecipitated TaOST1B^{Hap1} and TaOST1B^{Hap4} proteins transiently expressed in wheat protoplasts. The target proteins were digested with chymotrypsin, and the identified peptide coverage for TaOST1B^{Hap1} and TaOST1B^{Hap4} was 43.45% and 27.12%, respectively. Among these, N-terminal peptides accounted for approximately 46.15% and 38.10%, respectively. The results showed that the mature N-terminal peptide of TaOST1B^{Hap1} was identified as AQAVGDGIRVLSAEKRIDL.T, whereas that of TaOST1B^{Hap4} was AGDGIRVLSAEKRIDL.T, they have different signal peptide cleavage site and generate distinct mature N termini (Figure bellow). The detailed information were provided in the Source Data file S9C. The results suggest that the signal peptide sequence polymorphisms of TaOST1B^{Hap1} and TaOST1B^{Hap4} lead to differences in N-terminal sequences of the mature proteins,

which may affect the interaction of TaOST1B with P1. However, these precise molecular mechanism by which requires deep investigation. We have therefore included a discussion in lines 492-495 of the revised manuscript.

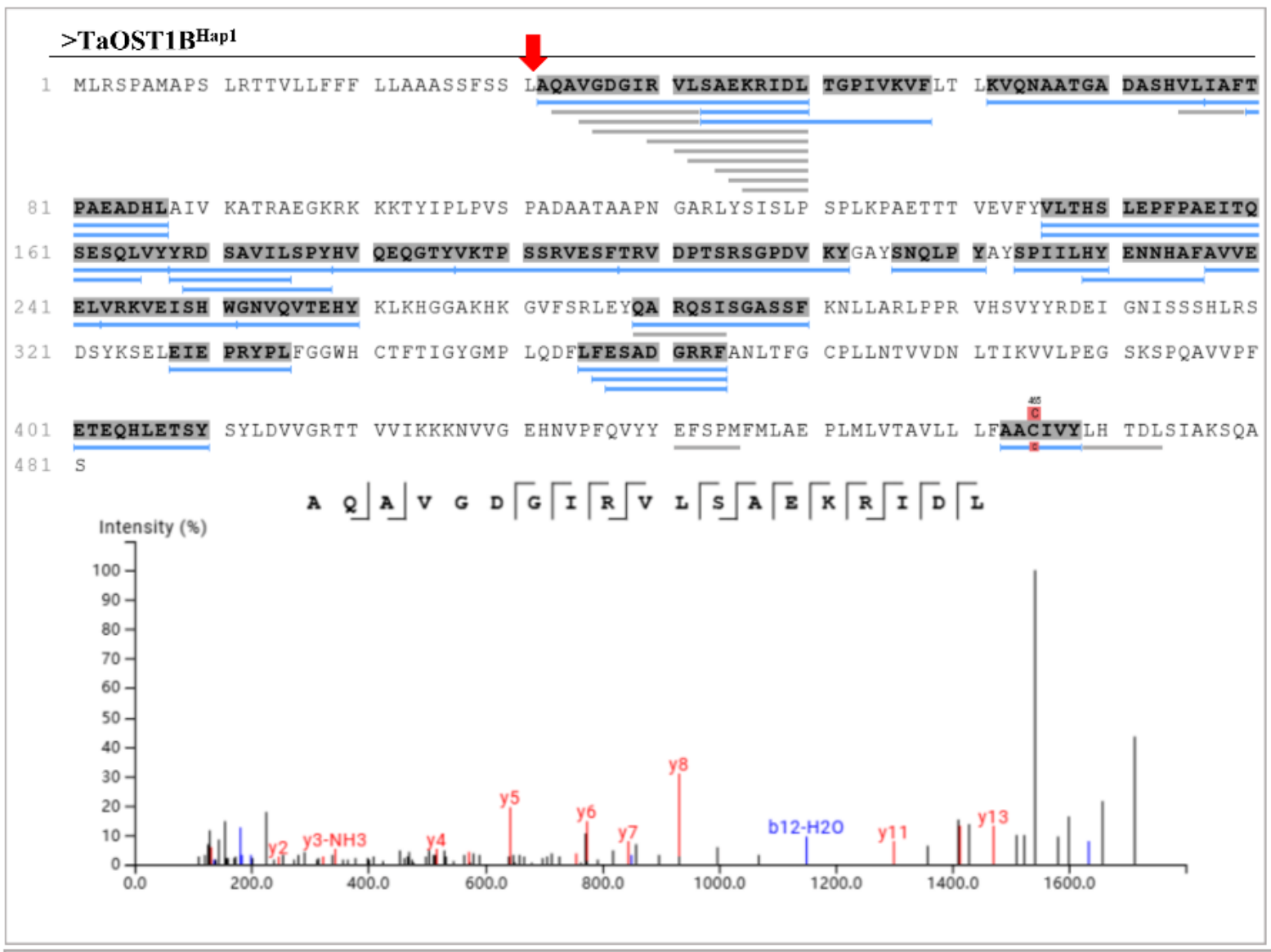

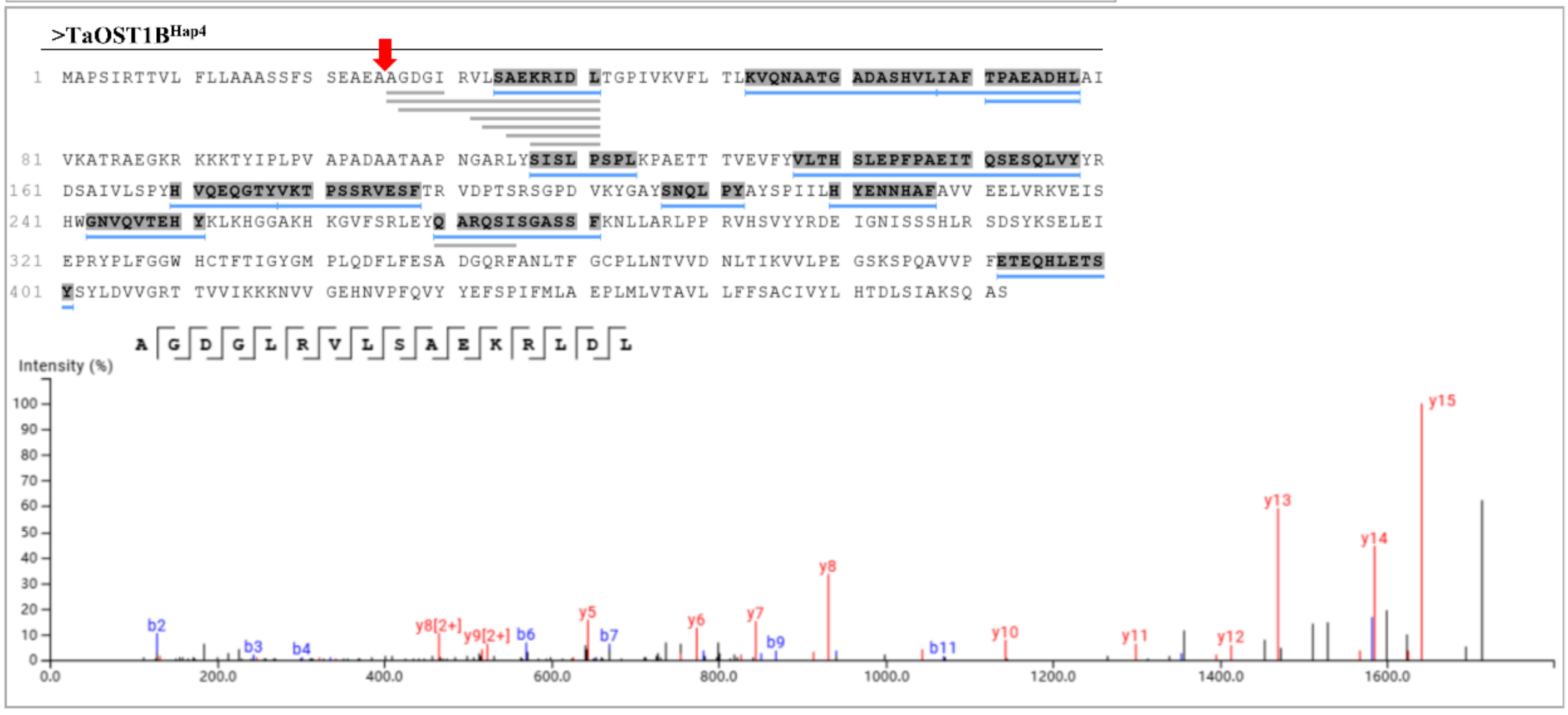

2. The displayed protein-protein interactions are not entirely reliable due to their inherent limitations, which may result in the failure to detect genuine, biologically relevant interactions. The presence or absence of the signal peptide or individual amino acids may cause misfolding or poor expression, resulting in negative LCI results that are misinterpreted as a lack of interaction, when in fact they may be due to a lack of expression. I am concerned because the cLuc domain in the pCAMBIA1300-cLuc system is fused to the N-terminus of TaOST1B, masking the signal peptide so that it is neither recognized as a signal peptide nor cleaved. The same applies to the Y2H system, and both systems are out of their subcellular context, as TaOST1B is likely a membrane-anchored protein, with most of the protein in the ER lumen. The authors should

carefully consider their data and provide additional controls or evidence. For example, show more co-IP data of plant-produced proteins like TaOST1B-Hap1 and TaOST1B-Hap4 with P1.

Response: Thank you for your comment and suggestion. Following your recommendation, we constructed TaOST1B^{Hap1} and TaOST1B^{Hap4} with GFP tag fused to their the C-terminal region，and further performed Co-IP assays by transiently co-expressing P1-Flag with TaOST1B^{Hap1}-GFP or TaOST1B^{Hap4}-GFP in wheat protoplasts, using co-expression of P1-Flag with GFP as a control. The results showed that TaOST1B^{Hap1}-GFP interacted with P1-Flag, but TaOST1B^{Hap4}-GFP did not (Figure below). This finding has been incorporated into lines 431-432 of the revised manuscript.

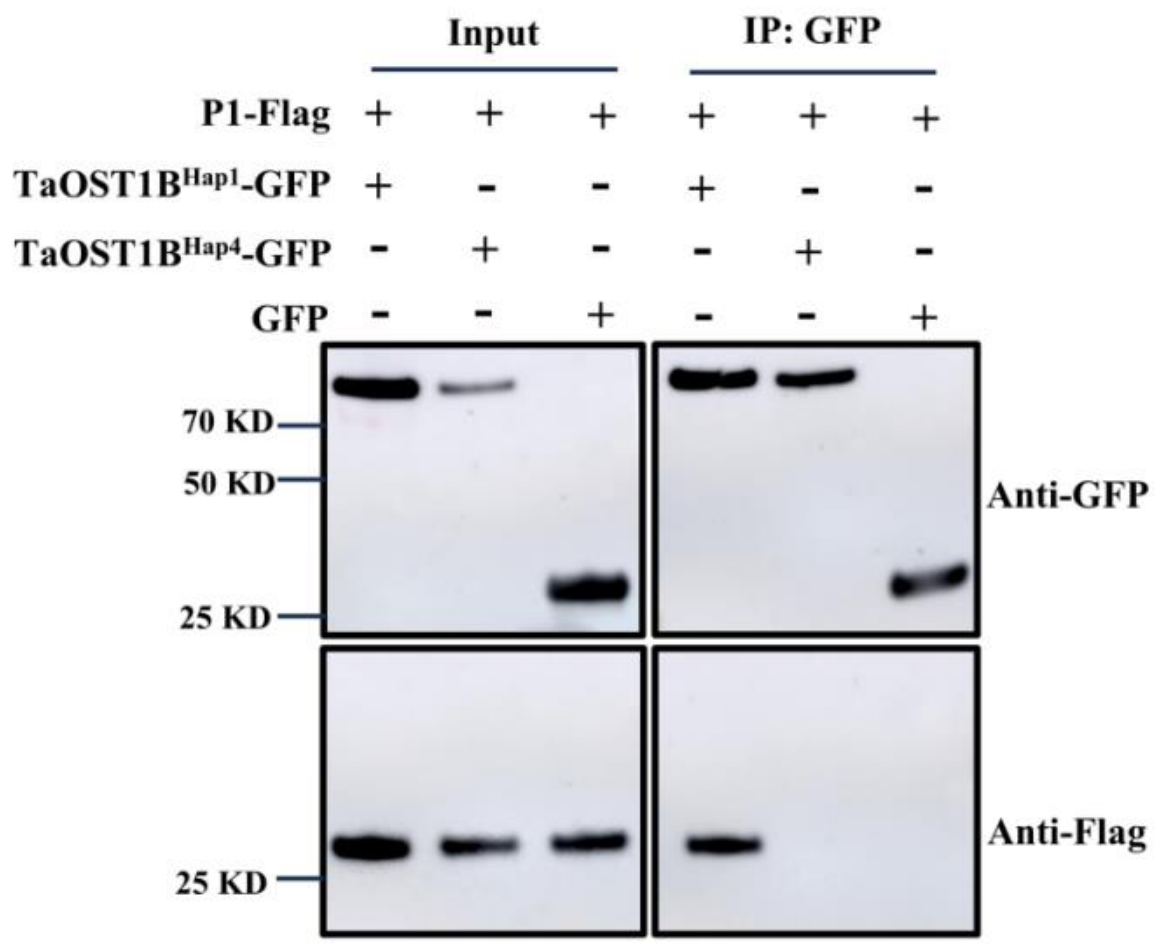

3. P1 is translated in the cytosol and a previous study (Sun et al., 2014 DOI: 10.1111/mpp.12109) has shown that it is associated with the ER membrane, presumably on the cytosolic side. The N-glycosylation by the oligosaccharyltransferase complex takes place in the lumen of the ER. So where is P1 localized in the infected plant cells and how is it targeted to the ER lumen for glycosylation? Does it have a signal peptide? Is it in the ER? The shown confocal images of P1 are not entirely clear and differ from the images of Sun et al. 2014. Since it frequently happens that the fluorescent protein tags are cleaved off in the cytoplasm it is important to show relevant immunoblots of the intact fusion proteins without any quantitative amounts of free RFP/GFP that may label the cytosol and nucleus. The provided blots in the supplement lack the region below 40 KD.

Response: Thank you for your questions and comment. To clarify these issues, we examined the subcellular localization of P1-GFP and P1^{N116A}-GFP in plant leaves infiltrated with WYMV infectious clones using confocal microscopy. Our results showed that P1-GFP was localized not only in the nucleus but also at the ER (Figure below). As P1 lacks a canonical signal peptide, we speculate that it

may access the ER lumen and undergo N-glycosylation during infection through non-canonical or host-assisted mechanisms. Similar targeting and glycosylation within the ER lumen have been reported for several viral proteins, such as hepatitis C virus E1/E2 and flavivirus NS1, which rely on complex membrane-associated or polyprotein-processing mechanisms (Goffard & Dubuisson, 2003; Huang et al., 2024). While the precise mechanism by which P1 is targeted to the ER lumen remains to be determined, we have included a discussion of this point in lines 495-502 of the revised manuscript. To rule out artifacts resulting from cleavage of the fluorescent protein tag, we expressed N-terminal GFP-tagged P1 and P1^{N116A} and performed immunoblot analysis using a GFP antibody. The results confirmed the presence of intact GFP-P1 fusion proteins without detectable free GFP, and the blot included the region below 40 kDa (Figure below). Additionally, the clearer confocal images of P1 subcellular localization have been included in the Fig. S5 of the revised manuscript.

Goffard A, Dubuisson J. Glycosylation of hepatitis C virus envelope proteins. Biochimie. 2003, 85(3-4):295-301.

Huang S. et al., The glycosylation deficiency of flavivirus NS1 attenuates virus replication through interfering with the formation of viral replication compartments. Journal of Biomedical Science. 2024, 31(1):60.

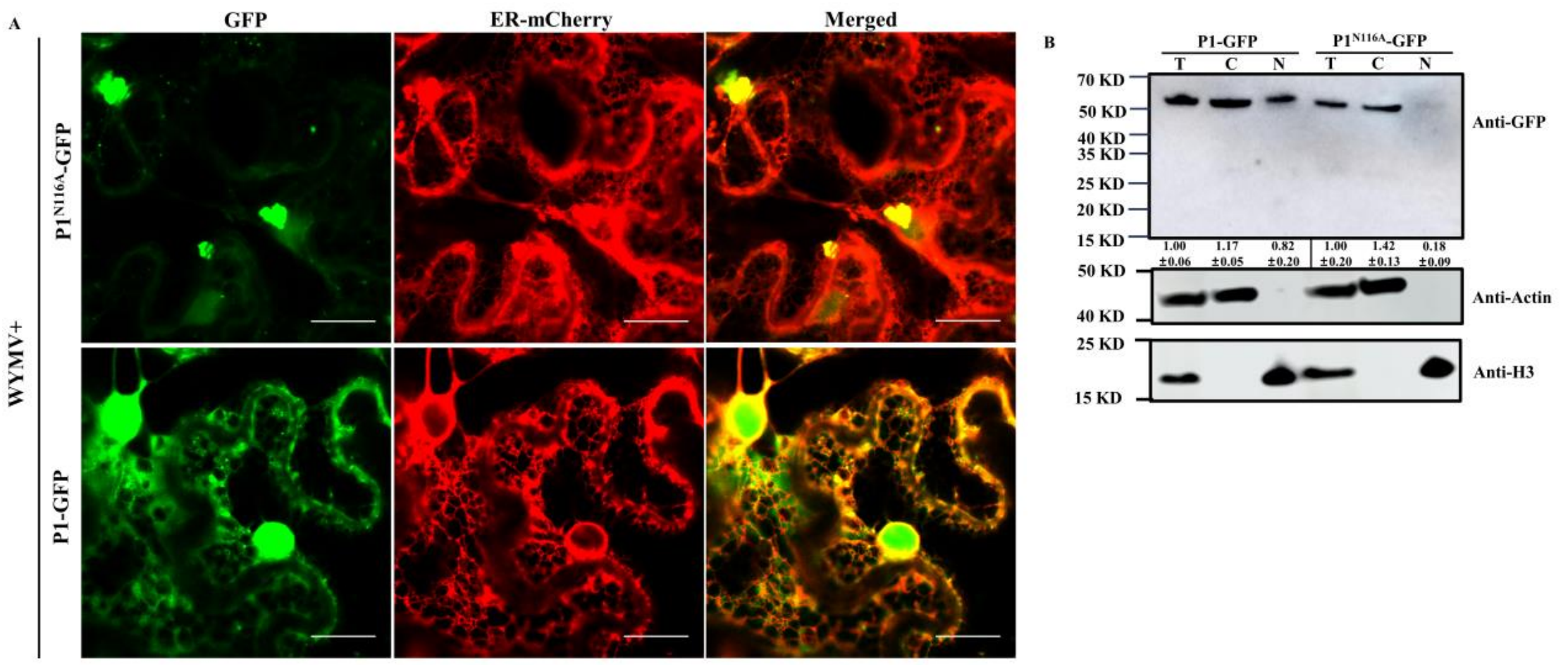

-Is P1-N116A-GFP in the ER as stated in line 292? This is not obvious from the shown images in S5C, where also the ER marker does not show a very clear ER labeling. Please provide better images. In addition, I would like to see also P1 co-expressed with the ER marker and the subcellular localization of both TaOST1B-Hap1 and TaOST1B-Hap4 with an ER marker.

Response: Thank you for your questions and comments. In response, we have provided confocal images showing the co-localization of P1^{N116A}-GFP, P1-GFP, TaOST1B^{Hap1}-GFP and TaOST1B^{Hap4}-GFP with ER marker. The results showed that P1^{N116A}-GFP was clearly distributed on the ER-associated aggregates, and P1 was co-localized with the ER in addition to the nucleus. Both

TaOST1B^{Hap1} and TaOST1B^{Hap4} were localized to the ER, with no obvious differences between the two (Figure below). These confocal images have now been included in the Fig. S3D and Fig. S5C of the revised manuscript.

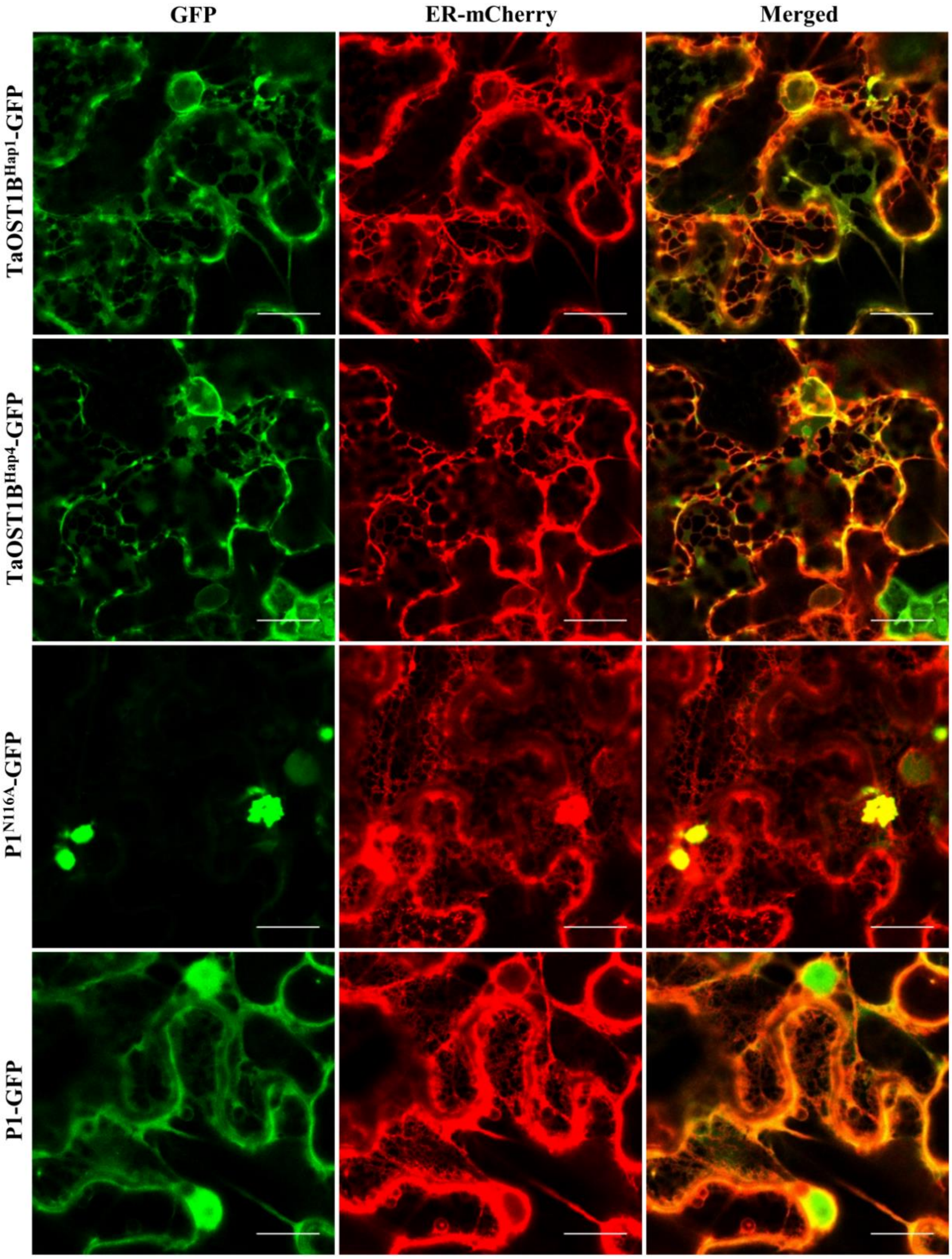

-The confocal data with H2B-RFP in Fig. 4C and 6D display also chloroplasts. For clarity it would be better to filter them out or at least mention that the nucleus and chloroplasts are visible in the images. Is H2B-RFP also present in the images in Fig. 6D, it appears so, but it is not

indicated. Also, please mention that the RFP signal was used as a reference for the GFP signal.

Response: We thank the reviewer for this careful observation. To clarify, the signals shown in Figs. 4C and 6D do not represent chloroplast autofluorescence. Instead, at lower magnification, nuclei from multiple guard cells are visible within the field of view, which may appear similar to chloroplast-like signals. To avoid confusion, we have revised the figure legends to explicitly indicate that the observed signals correspond to nuclei in revised manuscript. We also confirm that H2B-RFP is present in Fig. 6D, and this has been noted accordingly. In addition, we have clarified that the RFP signal was used as a reference for the GFP signal.

4. Detection of the P1 glycosylation is essential for the overall conclusions. Yet the shown data raise some questions. Upon digestion with PNGases a shift in mobility is detectable for P1-PNGase or upon incubation with TM, but P1-N116A which lacks the N-glycosylation does not display a difference in mobility compared to P1. Do you have an explanation? To confirm the glycosylation of P1 from WYMV infected wheat, protein extracts from infected plants should be digested with PNGases and the shift in mobility should be shown with the P1 antibody as used in Fig. S5. Due to the lower molecular weight of P1 compared to P1-RFP the differences should be better visible. Moreover, also the N fraction in Fig. 5D should be digested with PNGases to confirm that glycosylated P1 is in the nucleus.

Response: Thank you for your question and suggestions. We acknowledge that in our previous figure, the mobility shift between $P1^{N116A}$-GFP and P1-GFP was not clearly resolved. This may be because the mobility change resulting from the loss of a single N-glycosylation site is relatively subtle and was not readily distinguishable under the SDS-PAGE conditions used previously. Furthermore, when we conducted N-glycosylation detection on $P1^{N116A}$-GFP and $P1^{N116A}$-GFP treated with tunicamycin (TM) by LC-MS/MS, no abundant glycopeptides were detected for $P1^{N116A}$. To clarify this point, we reanalyzed the mobility shift of TM-treated and untreated P1-GFP and $P1^{N116A}$-GFP using gradient SDS-PAGE, which revealed a more discernible difference than previously observed (Figure below).

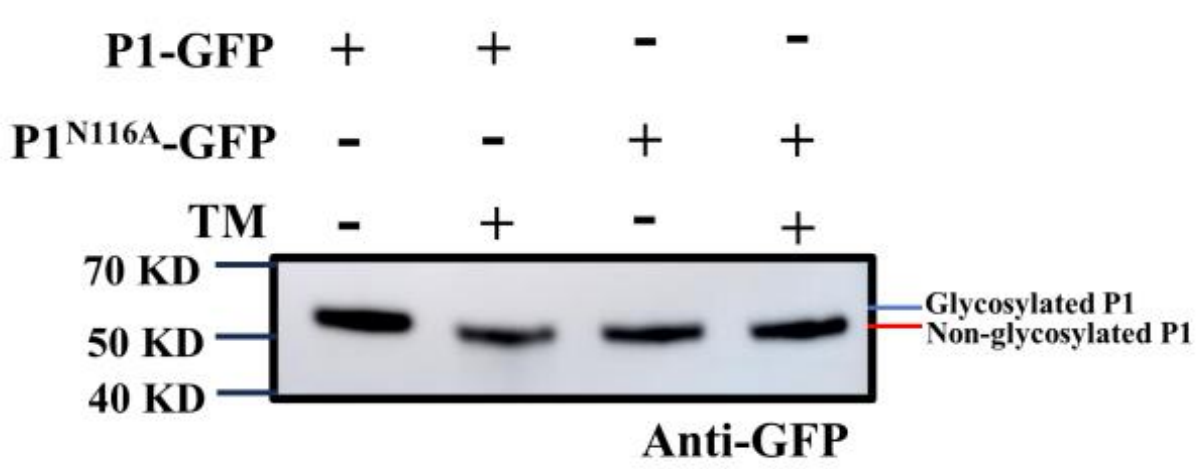

Following your's suggestion, we treated the immunoprecipitated P1 protein from WYMV-infected leaves and nuclear protein fractions from wheat protoplasts expressing P1-GFP with PNGase F. The mobility shift of P1 before and after digestion were analyzed using P1 antibody and GFP antibody, respectively. In the two groups of samples, PNGase F-treated P1 migrated faster than the untreated controls (Figure below). These results further support that P1 undergoes glycosylation and the glycosylated form is present in the nucleus. We have incorporated these findings into lines 250-254 and 363-367 of the revised manuscript.

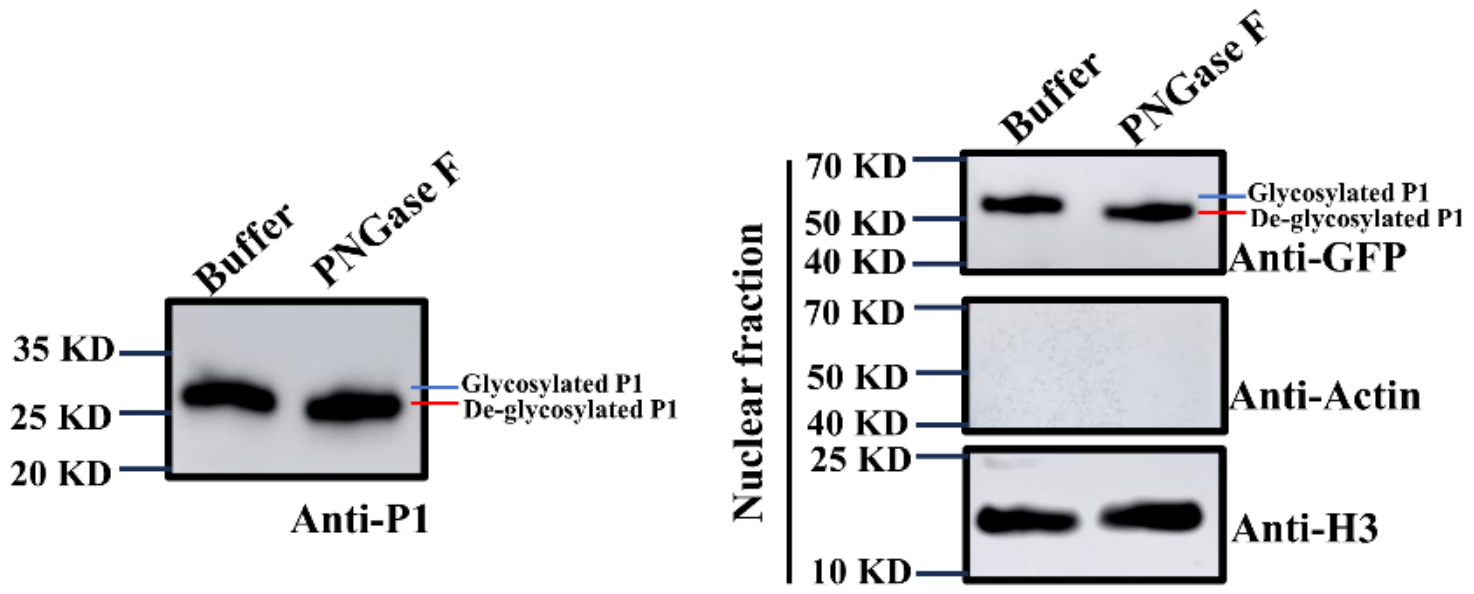

5. I did not understand how the intensity of glycopeptides for P1 was determined in Fig 3H. How was P1 purified from wheat? What does the graph show and how was it normalized? How much non-glycosylated peptide was identified compared to glycosylated P1 peptide? In my opinion it would be easier for the reader to provide relative amounts of glycosylated vs non-glycosylated peptide for every used line/condition. Also, the abundance data and spectra shown for the glycopeptides (Fig. 3F, 3G. 7C and Fig. S4) are not easy to understand. Please provide an additional table listing the relative amounts of all identified glycan structures.

Response: Thank you for your comments. To enrich P1 proteins, we performed immunoprecipitation from WYMV-infected *TaOST1B^{Hap1}* transgenic, knockout lines and wild type plants using anti-P1 antiserum coupled to protein G agarose beads. Following extensive washing, the bound proteins were collected and subjected to LC-MS/MS analysis. In multiple experiments, the detection of P1 glycopeptides was hindered by strong ion suppression caused by highly abundant non-glycosylated peptides. Therefore, we employed a glycopeptide enrichment strategy, which enabled the identification of multiple glycoforms of P1, albeit at the expense of losing non-glycosylated peptides. This approach, which integrates glycoform-level data to represent site-specific glycosylation, has been widely adopted in glycoproteomics workflows, such as SugarQuant and pGlycoQuant (Fang et al., 2020; Kong et al., 2022). Accordingly, we used glycoform-level data to evaluate the glycosylation status of P1 across

different wheat lines. To ensure data quality and reliability, the quantitative values obtained from pGlycoQuant were filtered, including missing value filtering and coefficient of variation (CV<0.3) control. The abundance values presented in the figures represent the summed intensities of the detected P1 glycoforms, while the corresponding spectra are provided to support qualitative identification. We have incorporated these information into the figure legends and lines 852-855 of the revised manuscript. The supplementary tables listing the intensities of identified P1 glycoforms were listed in Data S5.

Fang P, Ji Y, Silbern I, et al. A streamlined pipeline for multiplexed quantitative site-specific N-glycoproteomics[J]. Nature Communications, 2020, 11(1): 5268.

Kong S, Gong P, Zeng W F, et al. pGlycoQuant with a deep residual network for quantitative glycoproteomics at intact glycopeptide level[J]. Nature Communications, 2022, 13(1): 7539.

6. In many of the shown immunoblot data there are quantitative data without any statistics. Yet, in the text, "significant" differences are frequently mentioned. Please add the relevant statistics, define the error bars and number of replications and how the bands were normalized. All this information is missing (e.g. for Fig 4E).

Response: Thank you for your comment. We have now revised all relevant figures. These detailed information on statistical analysis were added in Source Data files. Specifically, the number of biological replicates is now indicated in figure legends, and error bars are defined as mean ± SD. Statistical significance was assessed using two-tailed *t*-tests or the ANOVA tests, and the corresponding *P* values are now provided. For bands quantification, signal intensities were measured using ImageJ and normalized to the corresponding controls. Detailed analysis data is provided in Source Data files.

7. The degradation assay only showed small differences, and it is questionable whether any of the shown differences are statistically significant. How informative is the semi-*in vivo* degradation assay? This assay was done using protein extracts. The used buffer is not clearly defined in the methods (e.g. which "protease" cocktail was used?) and likely includes a reducing agent and several protease inhibitors. While none of these will likely inhibit the proteasome, there is the question whether any conclusion can be made related to the involvement of autophagy. The inhibition of trafficking by wortmannin is not relevant in a non-cellular context and vacuolar proteases might be inhibited by the presence of the inhibitor cocktails and PMSF in the extraction buffer. Please comment on that and consider performing an in vivo assay with wheat protoplasts to provide clearer data on the involvement of the proteasome.

Response: Thank you for your comment. We referred to the methods for semi-*in vivo* degradation assays reported by Shen et al. (2016) and Guo et al. (2025), and further revised the Methods section in lines 880-881 to provide a detailed description of the extraction buffer composition, including the specific protease inhibitor cocktail used. We acknowledge that the presence of reducing agents and protease inhibitors may affect certain degradation pathways, particularly vacuolar proteases. The involvement of the autophagy pathway cannot be adequately assessed using semi-*in vivo* assays. Following your suggestion, we have referenced the protocol reported by Mituła, F et al. (2015) and performed *in vivo* degradation assays using wheat protoplasts. The results showed that treatment with MG132 led to a clear increase in the protein accumulation levels of both P1-GFP and $P1^{N116A}$-GFP compared with the DMSO control, with a more pronounced effect observed for $P1^{N116A}$-GFP. In contrast, wortmannin treatment did not result in an obvious increase (Figure below). These results indicated that $P1^{N116A}$ is predominantly degraded via the proteasome pathway. We have incorporated this protocol and the corresponding results into lines 399-405 and 892-896 of the revised manuscript.

Shen Q, Hu T, Bao M, et al. Tobacco RING E3 ligase NtRFP1 mediates ubiquitination and proteasomal degradation of a geminivirus-encoded βC1[J]. Molecular Plant. 2016, 9(6): 911-925.

Guo J, Zhang T, Xie H, et al. An m6A methyltransferase confers host resistance by degrading viral proteins through ubiquitination. Nature Communications. 2025, 16(1): 4821.

Mituła, F, Kasprowicz-Maluśki, A, Michalak, M, et al. (2015). Protein degradation assays in *Arabidopsis* protoplasts. Bio-protocol 5(4): e1397.

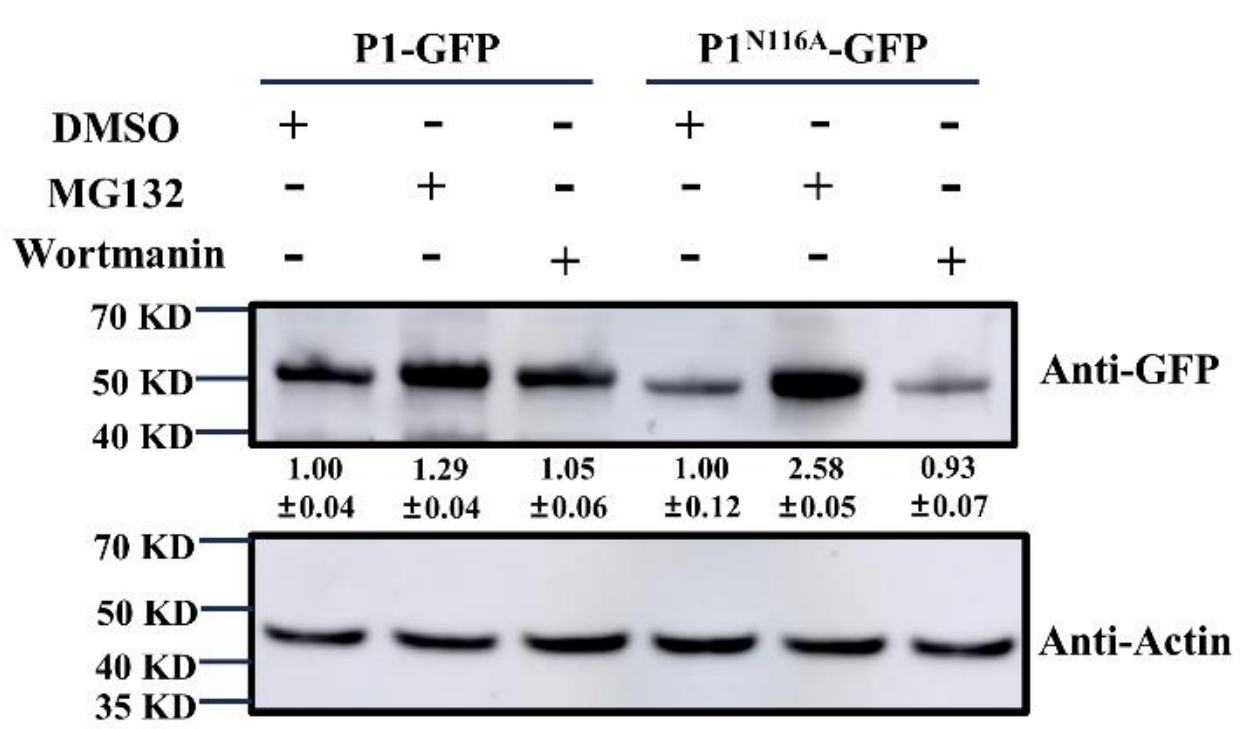

8. Absence of glycosylation may cause misfolding. It is suggested that nonglycosylated P1 may not fold correctly and thus trigger ER stress. However, attachment of an NLS and targeting of nonglycosylated P1 to the nucleus seems to restore the P1 suppressor function. Is this possible when nonglycosylated P1 is misfolded? Does P1-N116A-NLS trigger ER stress, is it degraded by

the proteasome and does it interact with TaIMP-alpha2? The results of these experiments will provide valuable insights into the role of the P1 N-glycan.

Response: Thank you for your comments and questions. The qRT-PCR analysis showed that, compared with GFP expression, the expression of P1^{N116A}-NLS-GFP resulted in a slight upregulation of the ER stress marker gene *NbBIP* (Figure A below). *In vivo* degradation assays using wheat protoplasts indicated that P1^{N116A}-NLS was also partially degraded via the 26S proteasome pathway (Figure B below). Additionally, the Co-IP results showed that P1^{N116A}-NLS interacted with TaIMP-a2 (Figure C below). Our previous results showed that P1^{N116A} failed to interact with TaIMP2-a2, suggesting that the primary consequence of nonglycosylation is impaired binding to TaIMP2-a2, rather than causing global misfolding. Given that TaIMP2-a2 mediates nuclear import via recognition of NLS, addition of an NLS to P1^{N116A} restored both TaIMP2-a2 binding and nuclear localization, thereby rescuing its suppressor function. These findings indicate that the primary effect of nonglycosylation is on the nuclear targeting of P1, rather than on its overall protein folding. We have described and discussed these results in the revised the manuscript.

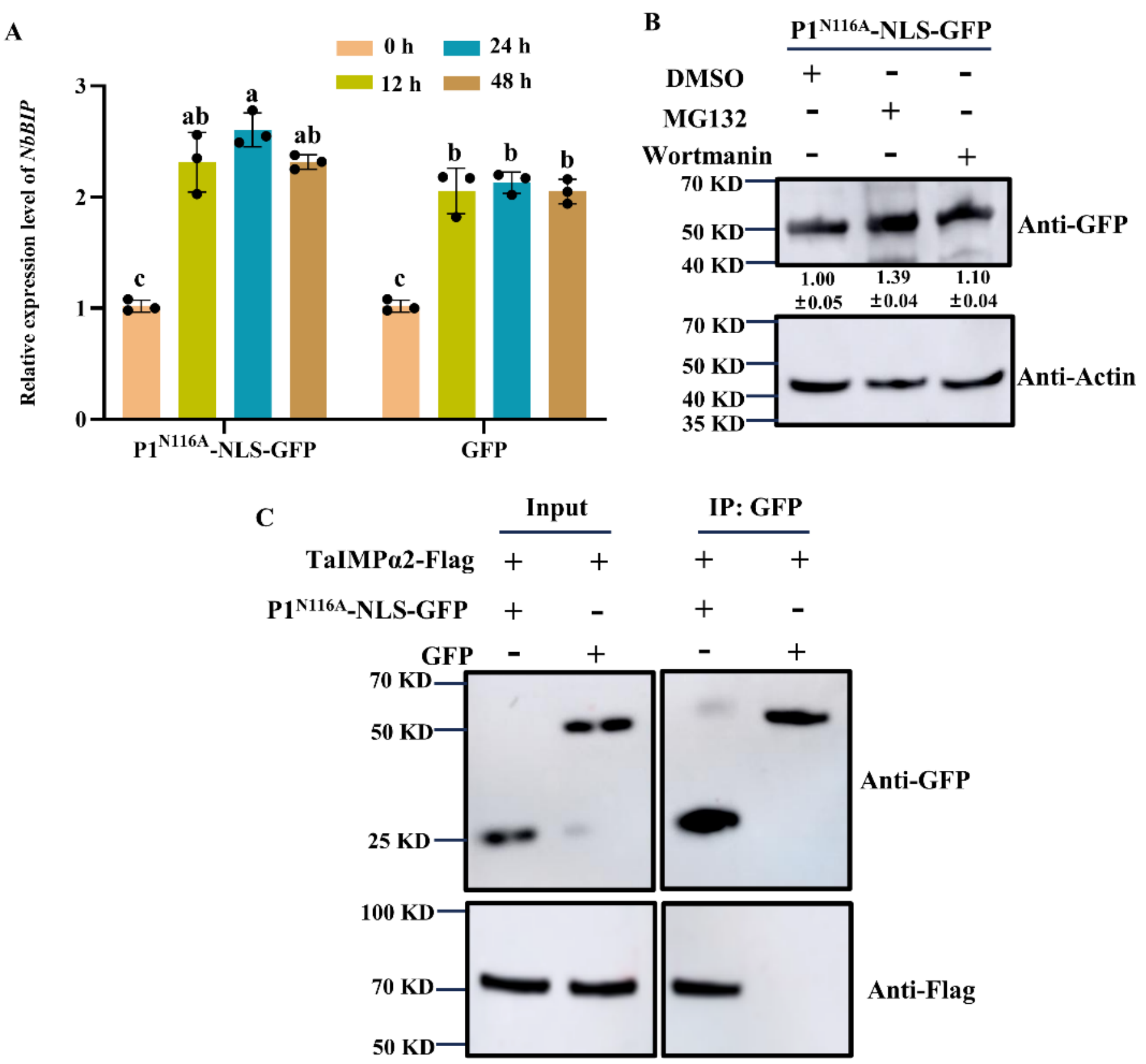

9. Related to the model shown in Fig. 8. How is P1 transported back from the ER to the cytosol

for localization in the nucleus? The presence of processed N-glycans instead of ER-characteristic mannosidic ones suggests that P1 is trafficking through the Golgi, this should be indicated in the illustration.

Response: Thank you for your comment. We propose that glycosylated P1 may follow the canonical ER-Golgi trafficking pathway, where its N-glycans are further processed in the Golgi to form mature protein subsequently localized in the nucleus. We have revised Fig. 8 to incorporate Golgi trafficking and to better reflect this updated model.

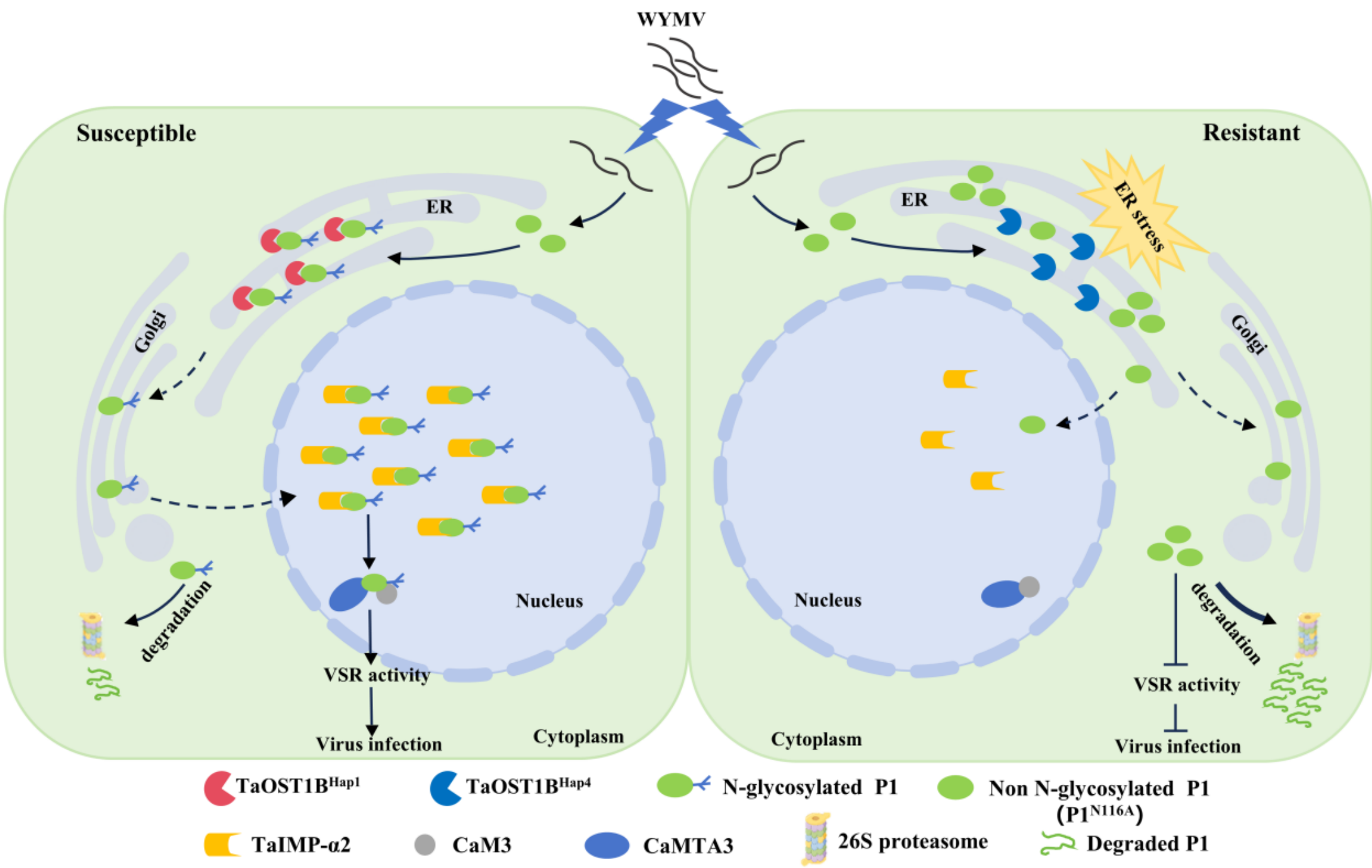

We sincerely thank the reviewer for constructive suggestions to further improve the manuscript. In the following, we respond point-by-point to all the comments and questions raised by the reviewer#4 and indicate corresponding changes made in the revised manuscript.

Reviewer #4 (Remarks to the Author):

The revisions have improved the clarity and presentation of the data, and the authors have addressed most of my concerns. However, there are still some issues that need to be addressed.

Major issue:

-Confirmation of the P1 glycosylation is essential for the overall conclusion. Yet the provided data and explanations are insufficient, and the MS data lack some clarity. The HILIC enrichment should be used for the MS-based identification of glycan structures linked to the P1 peptide. In addition to the MS/MS data, the full MS1 scans should be shown, all peaks related to glycopeptides should be annotated in the MS1 spectra and the relative intensities should be given in the Supplementary data.

Response: Thank you for your comment. We re-performed the LC-MS/MS analysis for P1 glycopeptides with HILIC enrichment, and performed LC-MS/MS-based quantification of P1 protein for the pre-enriched samples to minimize potential bias caused by HILIC enrichment. The relative intensities of enriched P1 glycopeptides normalized to total P1 protein across different samples is shown in Fig. 3F, 3G and 7D of the revised manuscript, the intensities and detailed MS data were provided in the Data S5. In addition, we have provided the MS1 spectra for representative glycopeptides of P1, and the peaks related to glycopeptide are indicated by red arrows in the MS1 spectra (Figures bellow). These figures have been incorporated into the Fig. S4A, S4C, Fig. S8B of the revised manuscript, and related description were added to figure legends in lines 66-69 and 148-150 of revised supplementary information file.

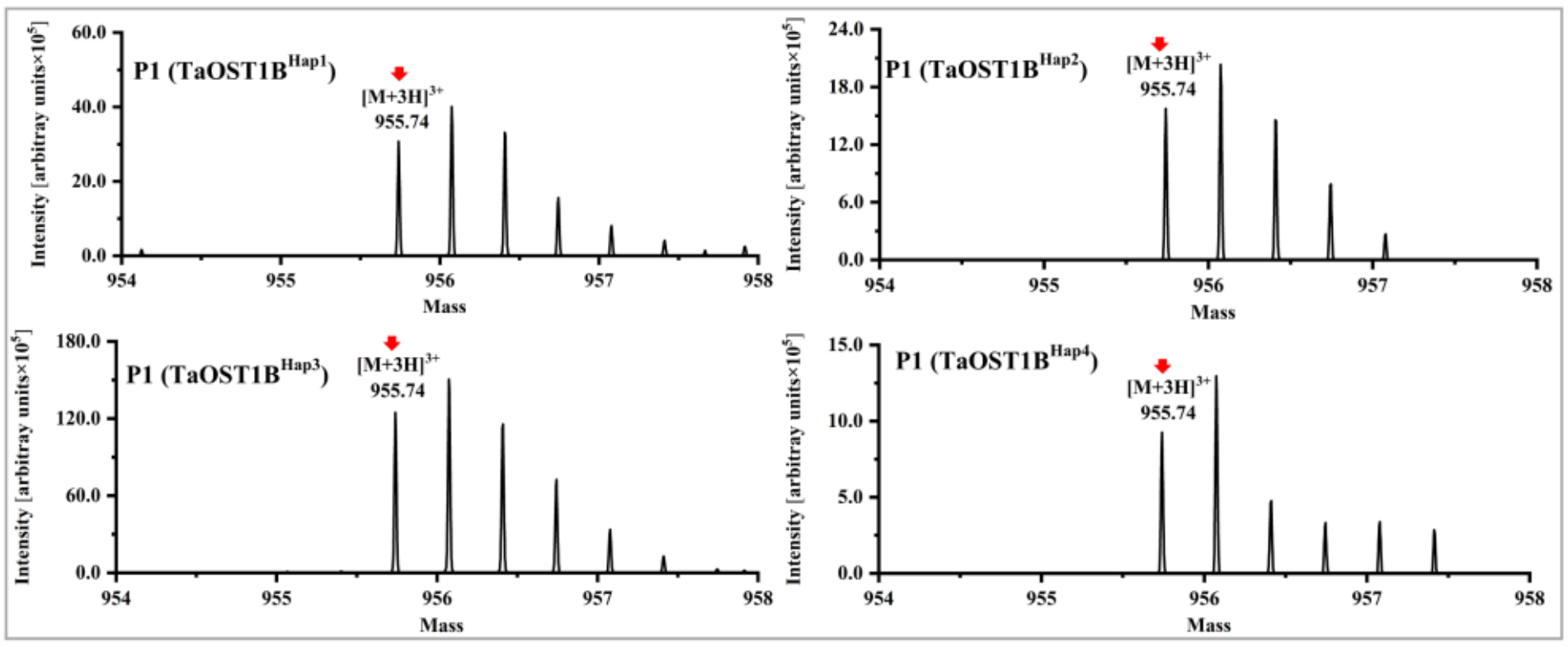

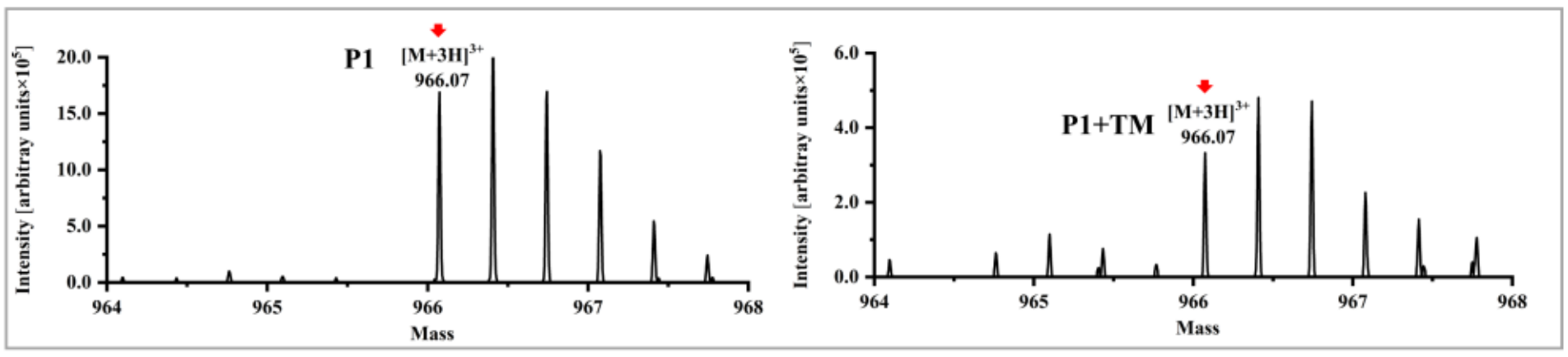

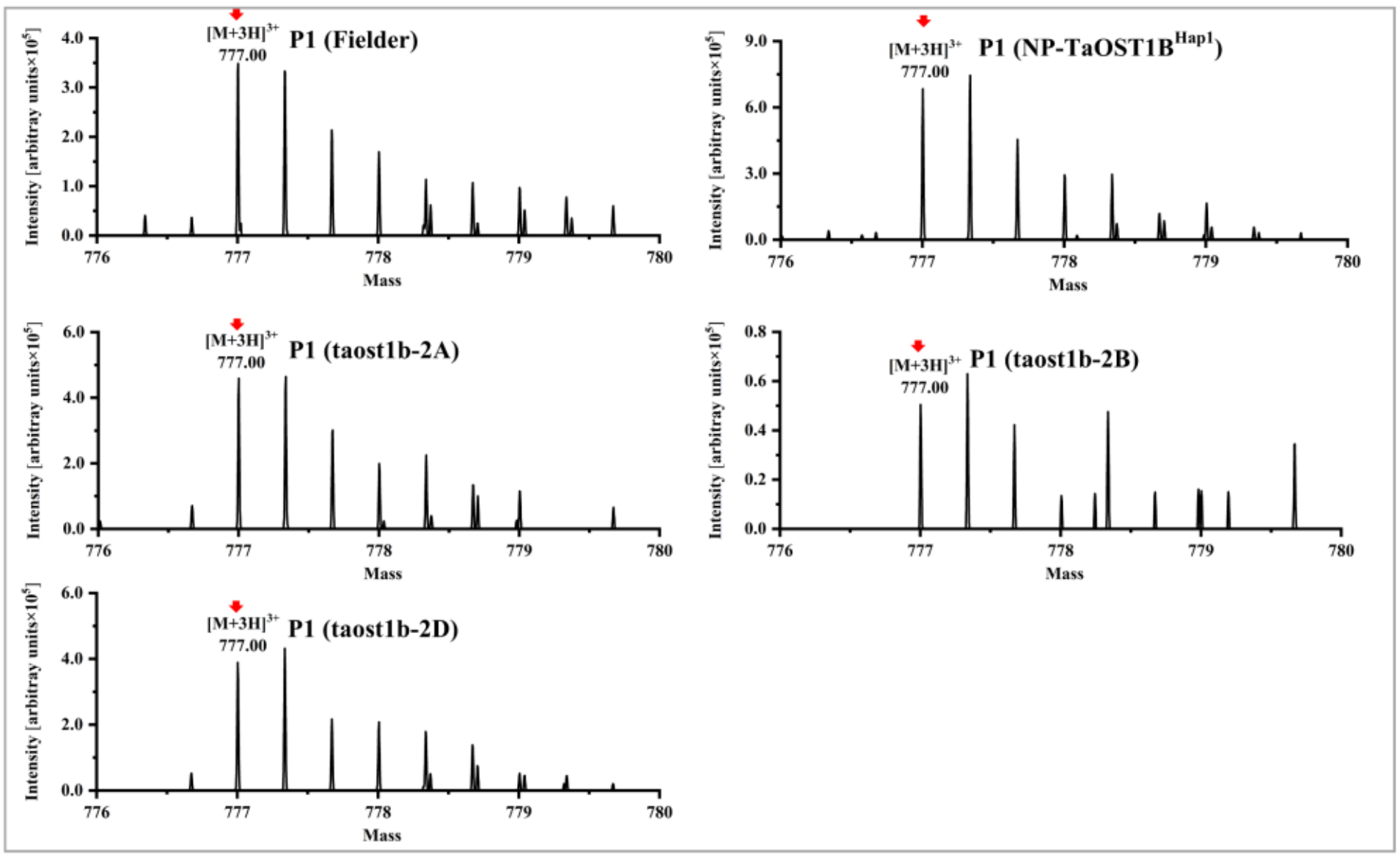

-The shown data for glycosylation intensity (Fig. 3F and 7D) are not informative and I would expect greater differences, for example, upon Tm treatment. This indicates a bias caused by the HILIC enrichment. To obtain more quantitatively balanced data on the amounts of non-glycosylated vs. glycosylated P1, the mixture of peptides and glycopeptides without HILIC enrichment needs to be compared. If the more highly abundant non-glycosylated peptides suppress the signal, then PNGase digestion of the glycopeptide is recommended. This will allow a more true relative comparison of glycosylated vs non-glycosylated P1. This data should be shown instead of the data shown in Fig. 3F and 7D.

Response: Thank you for your comment. Following your recommendation, we have performed LC-MS/MS analysis on PNGase F-digested P1 peptide without HILIC enrichment. By comparing the intensities of glycosylated and non-glycosylated P1 peptide to assess their relative changes across different samples. The results showed that, compared with P1, the relative intensity of glycosylated vs non-glycosylated P1 peptides was significantly reduced in the P1+TM sample (Figure A bellow). In WYMV-infected wheat lines, the relative intensity of glycosylated vs non-glycosylated P1 peptides was significantly increased in NP-TaOST1B^{Hap1} compared with Fielder, whereas it was significantly reduced in taost1b-2B, and no obvious differences in taost1b-2A or taost1b-2D (Figure B bellow). In

addition, the relative intensity of glycosylated vs non-glycosylated P1 peptides in TaOST1B^{Hap1} or TaOST1B^{Hap3} was more higher than in TaOST1B^{Hap2} or TaOST1B^{Hap4} (Figure C bellow). These results were consistent with the trends observed in our previous analyses. These results have been incorporated into the Fig. S4B, S4D, Fig. S8D and lines 259-261, 268-269, 438-440 of the revised manuscript. The detailed information for both glycosylated and non-glycosylated P1 peptides have been provided in the Data S6.

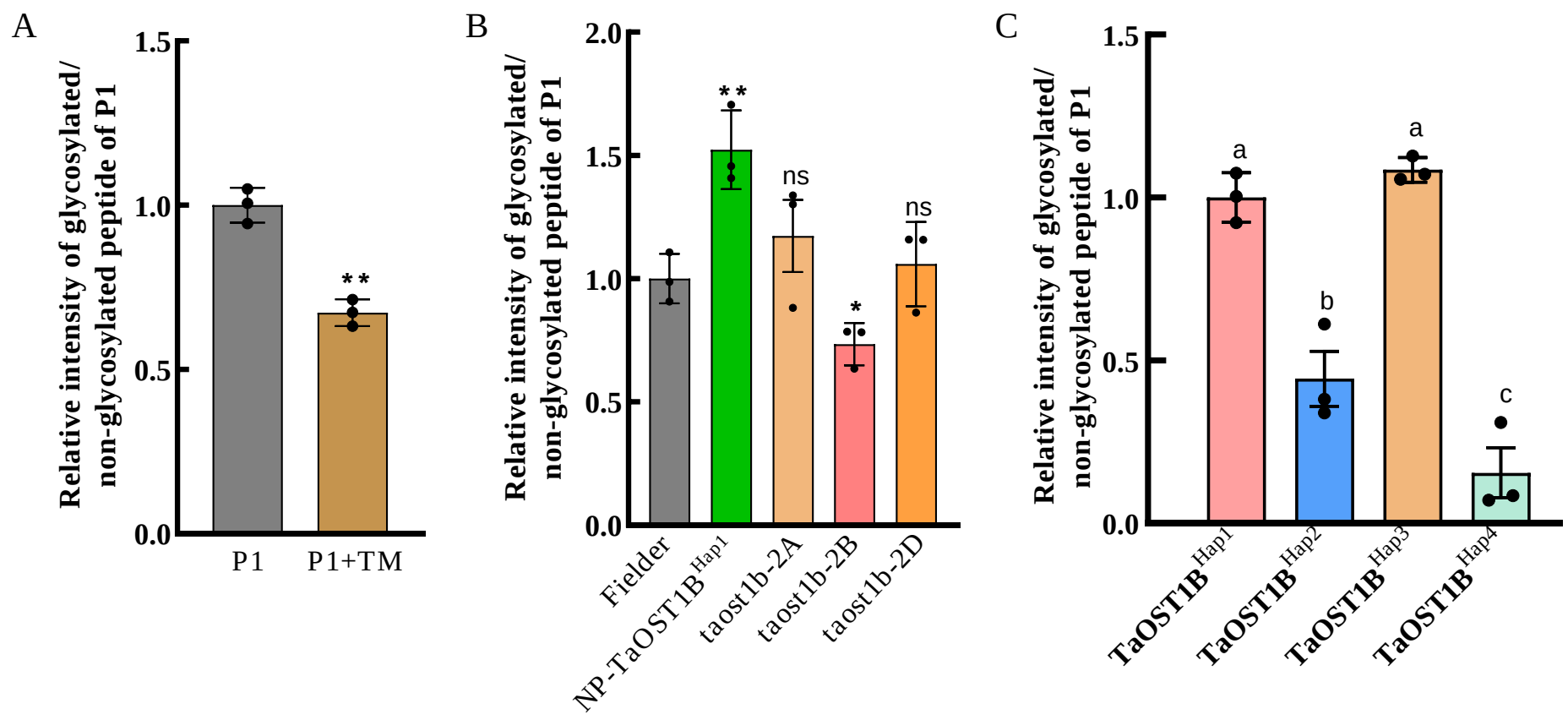

Minor points:

-In the text (e.g. lines 302, 305, 315, 371, 460, 464) significant is used to describe the differences. However, none of the shown data contains a statistic showing that the differences are indeed significant. Please change the text accordingly or provide the necessary information and statistic tests.

Response: Thank you for your comment. We have included the statistical analyses in the source data files. The manuscript text has been revised accordingly to ensure that the term "significant" is used only where supported by statistical evidence.

-The figure legends still lack some relevant information. For example, the shown variations (standard deviation?) are not defined (e.g. Fig. 4G, 6B, 6D, 7G, S2E, S5D,S6A, S6B, S7F, S12E).

Response: Thank you for your comment. We have revised all relevant figure legends to define the error bars and statistical information. The error bars are defined as mean ± SD, and the number of biological replicates (n=3) as well as the statistical tests used (two-sided *t*-test or one-way ANOVA tests) are indicated in the corresponding figure legends.

-Which wheat variety was used for the wheat protoplasts experiments or purification (Fig. S3E).

Response: Thank you for your question. Wheat protoplasts used in Fig. S3E were isolated from the

WYMV-infected wheat cultivar Yangmai 158. This information has been added to the figure legend in line 60 of the revised supplementary information file.

-In Figure 6D the 2h value for P1-GFP is 1.28, this is likely not correct.

Response: Thank you for your comment. This value was incorrectly labeled, it should be 0.28. We have corrected this error in the revised manuscript.

-Please describe more precisely how the P1 protein was purified (line 796).

Response: Thank you for your comment. We have described the process of P1 protein purification in lines 799-805 of the revised manuscript.

-Please define the used protease inhibitor cocktail (line 860, line 882).

Response: Thank you for your comment. We have added the product name and supplier of protease inhibitor cocktail in line 870 and line 892 of the revised manuscript.

-The semi-in vivo degradation assay still mentions the use of wortmannin (line 884).

Response: Thank you for your comment. We have corrected it in line 894 of the revised manuscript.

-In Figure S12, refer to panel "D" in the legend. Define the marker bands in D.

Response: Thank you for your comment. We have defined the molecular weight marker bands in Figure S12D.

We sincerely thank the reviewer for comments to further improve the manuscript. In the following, we respond to the comments raised by the reviewer and indicate corresponding changes made in the revised manuscript.

Reviewer #4 (Remarks to the Author):

I appreciate the authors' efforts to revise the manuscript and respond to prior concerns. However, the experimental issues related to the amount of glycosylated versus non-glycosylated P1 remain insufficiently addressed.

The provided data still do not show the relative proportion (%) of glycosylated P1 for each treatment and plant line used. It is also unclear why the reported relative intensity ratio of glycosylated to non-glycosylated P1 is 1. If this were the case, it would imply that 50% of total P1 is glycosylated and 50% is non-glycosylated, which appears unlikely. The glycosylation site occupancy (%) should instead be calculated as the intensity of the PNGase F digestion-derived deamidated peptide divided by the sum of the intensities of the deamidated peptide and the corresponding non-glycosylated (unmodified) peptide. I assume that these calculations are already included in the additional datasets; however, these data should also be presented clearly in the main body of the manuscript.

Response: Thank you for your comment. The relative intensity ratio of glycosylated versus non-glycosylated P1 was not 1 in the original datasets. In our previous analysis, the intensity values were normalized for relative comparison across samples, which may have caused confusion regarding actual proportion of glycosylated P1. Following your suggestion, we recalculated the data, the relative proportion of glycosylated P1 was calculated as the intensity of the PNGase F-derived deamidated P1 peptide divided by the sum of the intensities of the deamidated and corresponding non-glycosylated P1 peptides. These results were consistent with the trends observed in our previous analyses, the column plots have been revised (Figure below). The analysis data are described in lines 259-260, 267-268 and 438-439 of the main text of the revised manuscript, the corresponding column plots and detailed MS and calculated data are provided in the Figure S4B, S4D and S8D, Data S6).

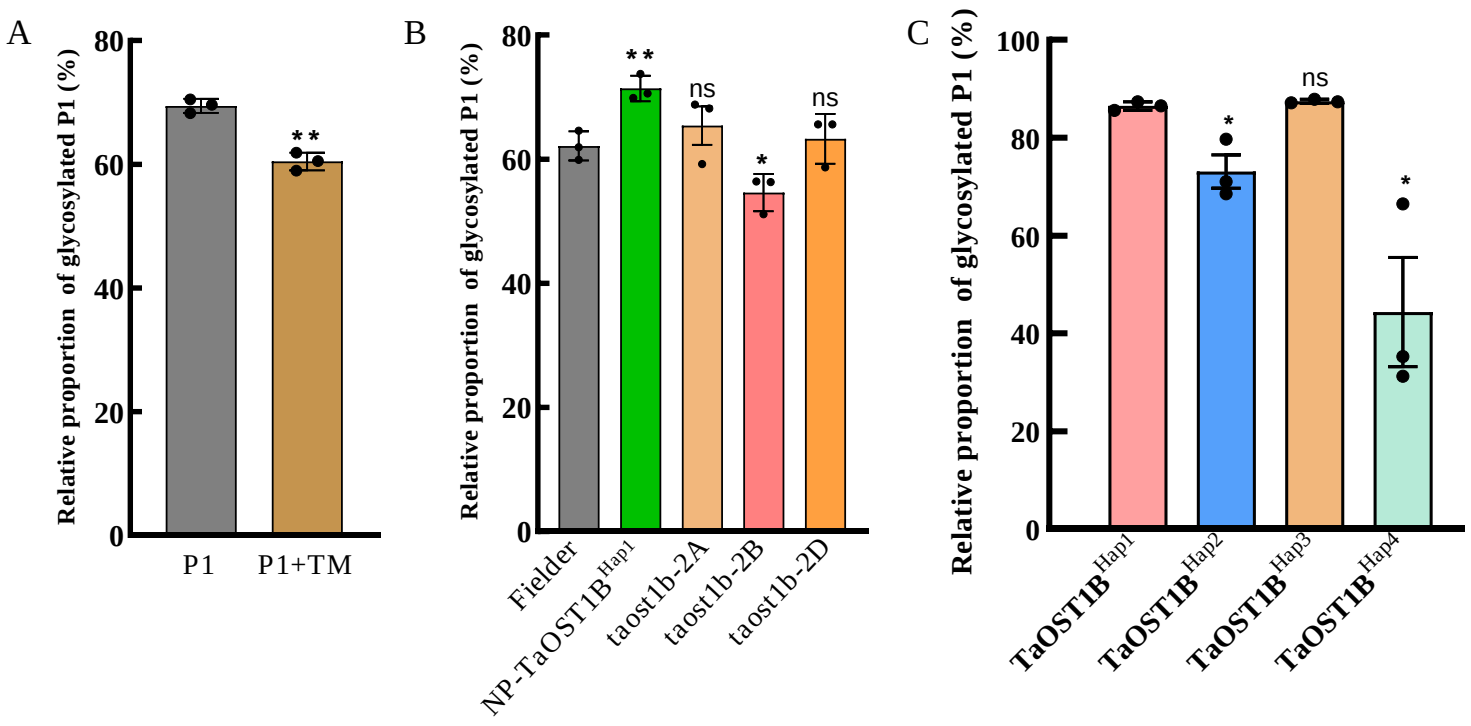

Supplement: Supplementary file 2 [file 41467_2026_75397_MOESM11_ESM.pdf]
