# Supplementary material for: The oligosaccharyltransferase TaOST1B promotes viral infection by enhancement of RNA silencing suppression in wheat
Source: Nat Commun. 2026 Jul 15;17:8688. doi: 10.1038/s41467-026-75397-8 (PMC13490409; doi:10.1038/s41467-026-75397-8)
Supplement: Supplementary file 3 [file 41467_2026_75397_MOESM1_ESM.pdf]

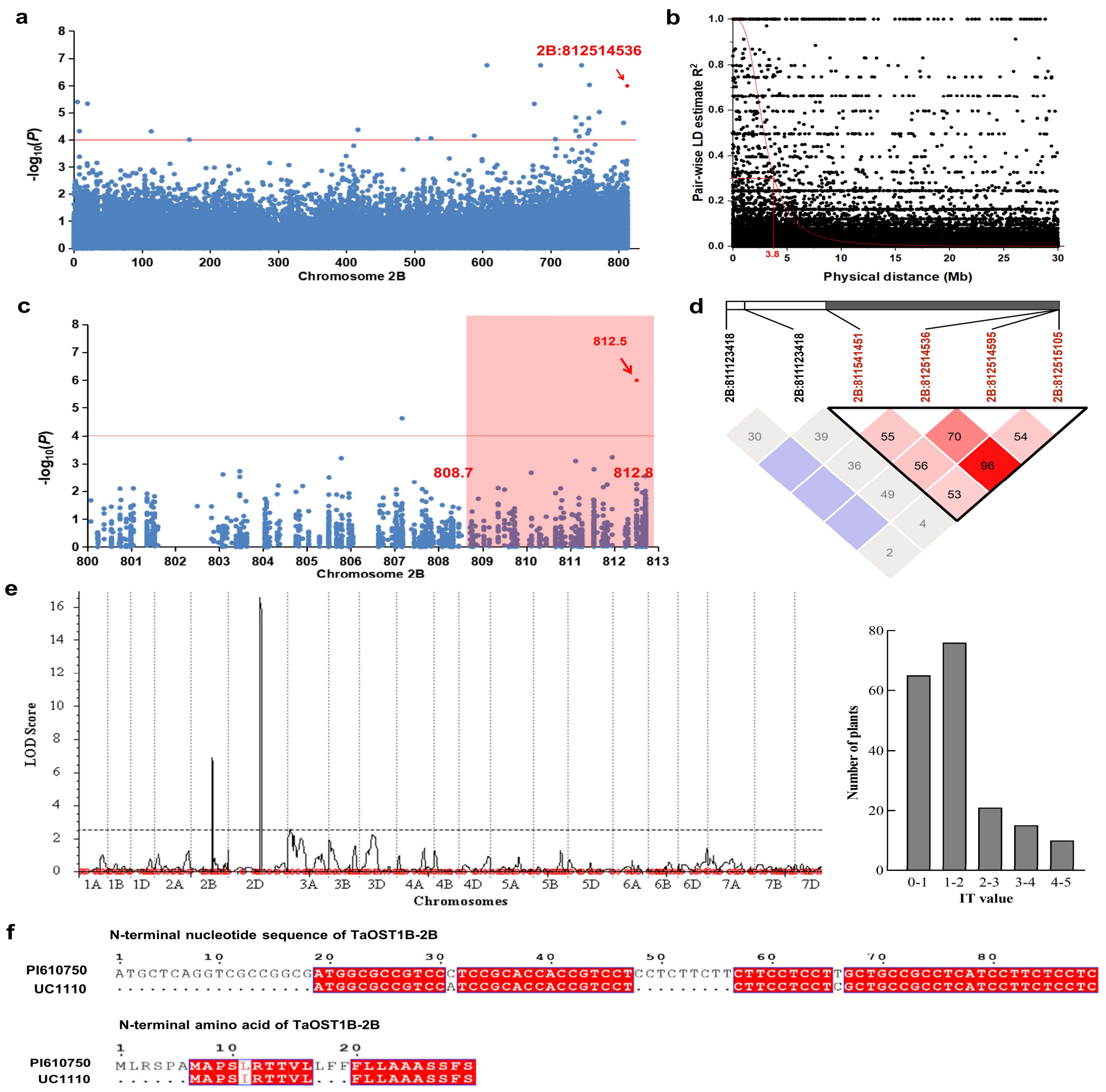

Supplementary Figure 1. Identification of WYMV resistance-related genes by GWAS and QTL mapping.

(a) The most significantly marker (2B:812514536) on 2B was identified by GWAS in the 480 Chinese wheat accessions. The red horizontal line indicates the genome-wide significance threshold. **(b)** Genome-wide average LD decay over genetic distances. Plot of pair-wise single-nucleotide polymorphism LD r^2 values as a function of intermarker map distance (Mb). **(c)** A WYMV candidate interval of 808.7-812.8 Mb on 2B was identified. Red background area indicates the candidate region, The red dots represent the most significant marker. **(d)** Haplotype analysis indicated a about 1.0 Mb block covering the lead SNP (2B:812514536) on chromosome 2B. **(e)** LOD contours for quantitative trait loci (QTL) to WYMV resistance identified by inclusive composite interval mapping (ICIM) in the PI610750/UC1110 (UP) population (LOD score > 2.5). Column chart on right show the distribution of IT (infection type) values for 187 UP-RIL

population in WYMV-contained nursery. **(f)** Alignment of coding sequences and amino acids in TaOST1B-2B N-terminal variation regions from PI610750 and UC1110. Source data are provided as a Source Data file.

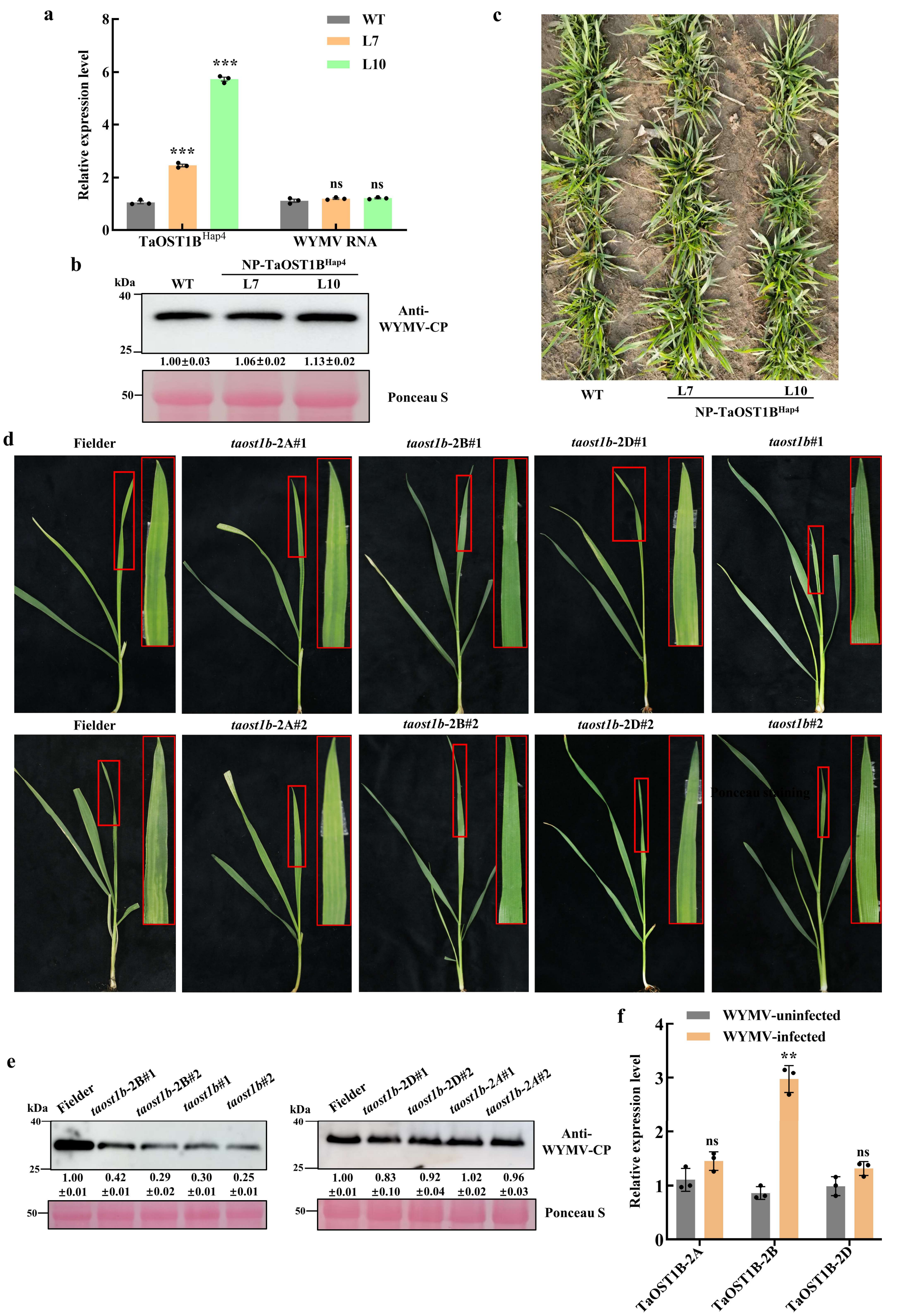

Supplementary Figure 2. (a) The relative expression levels of *TaOST1B*^{Hap4} (left) and accumulation of WYMV RNA (Right) in the WYMV-infected *TaOST1B*^{Hap4} transgenic lines and wild type (WT) plants were determined by qRT-PCR. *TaCDC* was used as the internal control gene. Values are means ± SD (two-sided *t*-test, n = 3 biologically independent samples), ***P <

0.001, and ns $P > 0.05$. **(b)** Detection of WYMV-CP accumulation in WT plants or two NP-TaOST1B^{Hap1} lines by western blot using a CP-specific antibody. Signal density of protein bands were quantified using Image J software, the values are the means ± SD. Ponceau staining showed samples loading. Three times the experiment was repeated with similar results. **(c)** Assessment of NP-TaOST1B^{Hap4} lines for diseases resistance in the WYMV-contaminated nursery at Yangzhou, Jiangsu Province in 2024. WT represents Fielder plants. **(d)** The phenotype of Fielder and the konckout lines inoculated with WYMV at 40 dpi. **(e)** Detection of WYMV CP accumulation in the konckout lines by western blot using a CP-specific antibody. Signal density of protein bands were quantified using Image J software, the values are the means ± SD. Ponceau staining showed samples loading. Three times the experiment was repeated with similar results. **(f)** Analysis of the expression levels for three homologous copies of *TaOST1B* before and after WYMV infection. *TaCDC* was used as the internal control gene, values are means ± SD (two-sided *t*-test, n = 3 biologically independent samples), $^{**}P < 0.01$ and ns $P > 0.05$. Source data are provided as a Source Data file.

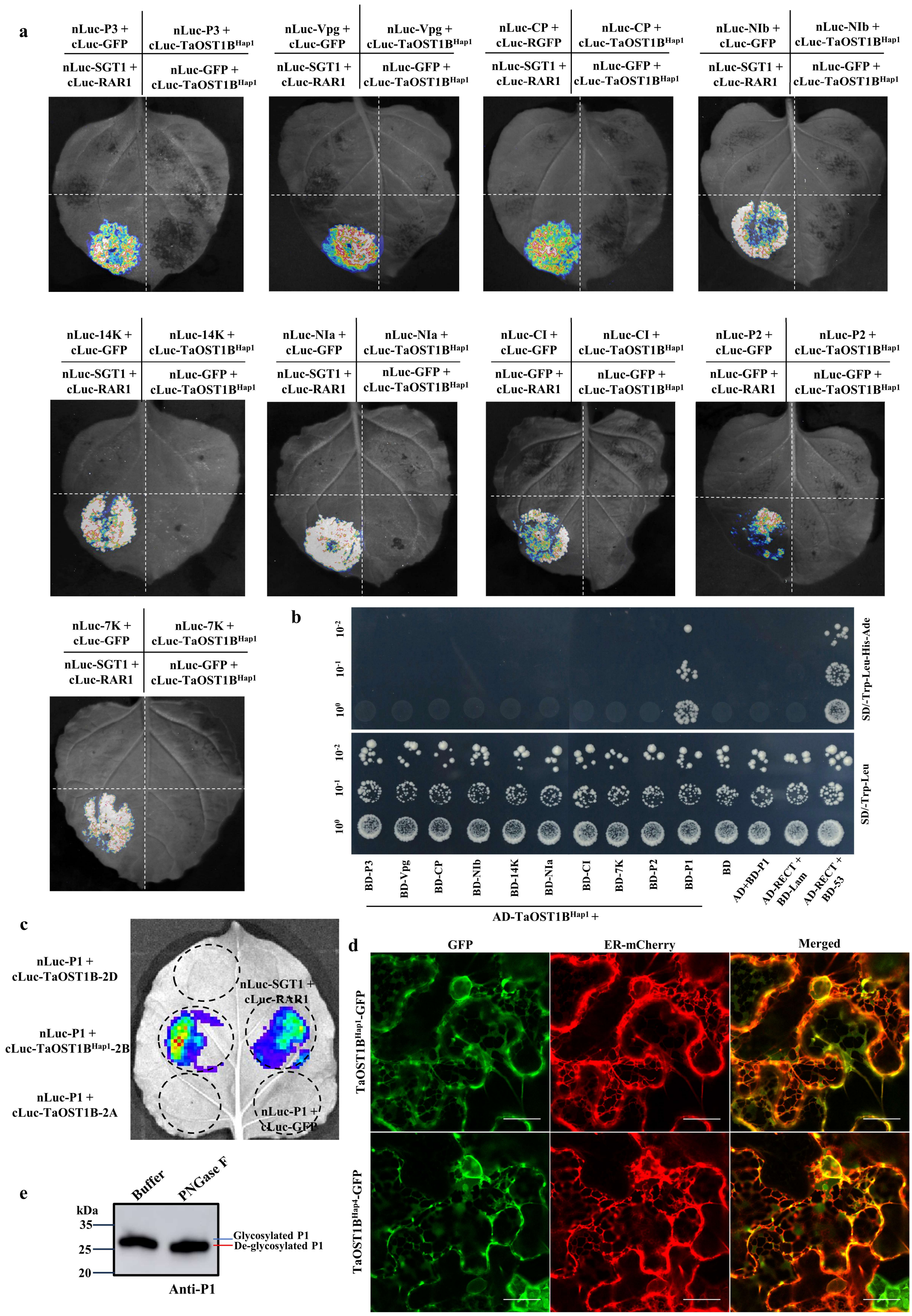

Supplementary Figure 3. Interaction of TaOST1B^{Hap1} with WYMV-encoded proteins.

(a) The leaf areas infiltrated with *Agrobacterium* cultures expressing nLuc-SGT1+cLuc-SAR were used as a positive control, nLuc-P3+cLuc-SAR, nLuc-Vpg+cLuc-SAR, nLuc-CP+cLuc-SAR, nLuc-NIb+cLuc-SAR, nLuc-14K+cLuc-SAR, nLuc-7K+cLuc-SAR, nLuc-NIa+cLuc-SAR,

nLuc-CI+cLuc-SAR, nLuc-P2+cLuc-SAR and nLuc-SGT1+cLuc-TaOST1B were used as negative controls, respectively. Luciferase activity was captured using a low-light cooled CCD imaging apparatus at 2 dpi. **(b)** TaOST1B^{Hap1} interacted with WYMV-encoded protein P1 in a yeast two-hybrid (Y2H) assay. AH109 strain cultures containing TaOST1B^{Hap1} in the GAL4 activation domain (AD) and WYMV coded-proteins in the GAL4 DNA-binding domain (BD) on selective medium. BD-Lam /BD-53and AD-RECT were used as negative and positive control, respectively. **(c)** Interaction of TaOST1B-2A, TaOST1B^{Hap1}-2B, and TaOST1B-2D with P1 was detected by LCI assay in *N. benthamiana* leaves. The leaf areas infiltrated with *Agrobacterium* expressing nLuc-SGT1+cLuc-RAR1 were used as a positive control, nLuc-P1+cLuc-GFP was used as negative controls, respectively. Luciferase activity was captured using a low-light cooled CCD imaging apparatus at 2 days post infiltration (dpi). **(d)** The TaOST1B^{Hap1}-GFP or TaOST1B^{Hap4}-GFP and ER-mCherry were co-expressed into the *N. benthamiana* leaves, the subcellular localizations were observed by confocal microscopy at 48 hpi. Bars indicate 20 μm. **(e)** P1 protein migration analysis of protein extracts from WYMV-infected “Yangmai 158” wheat plants treated with PNGase F glycosidases or a buffer control. Western blotting was performed using P1 specific antibody. Three times the experiment was repeated with similar results.

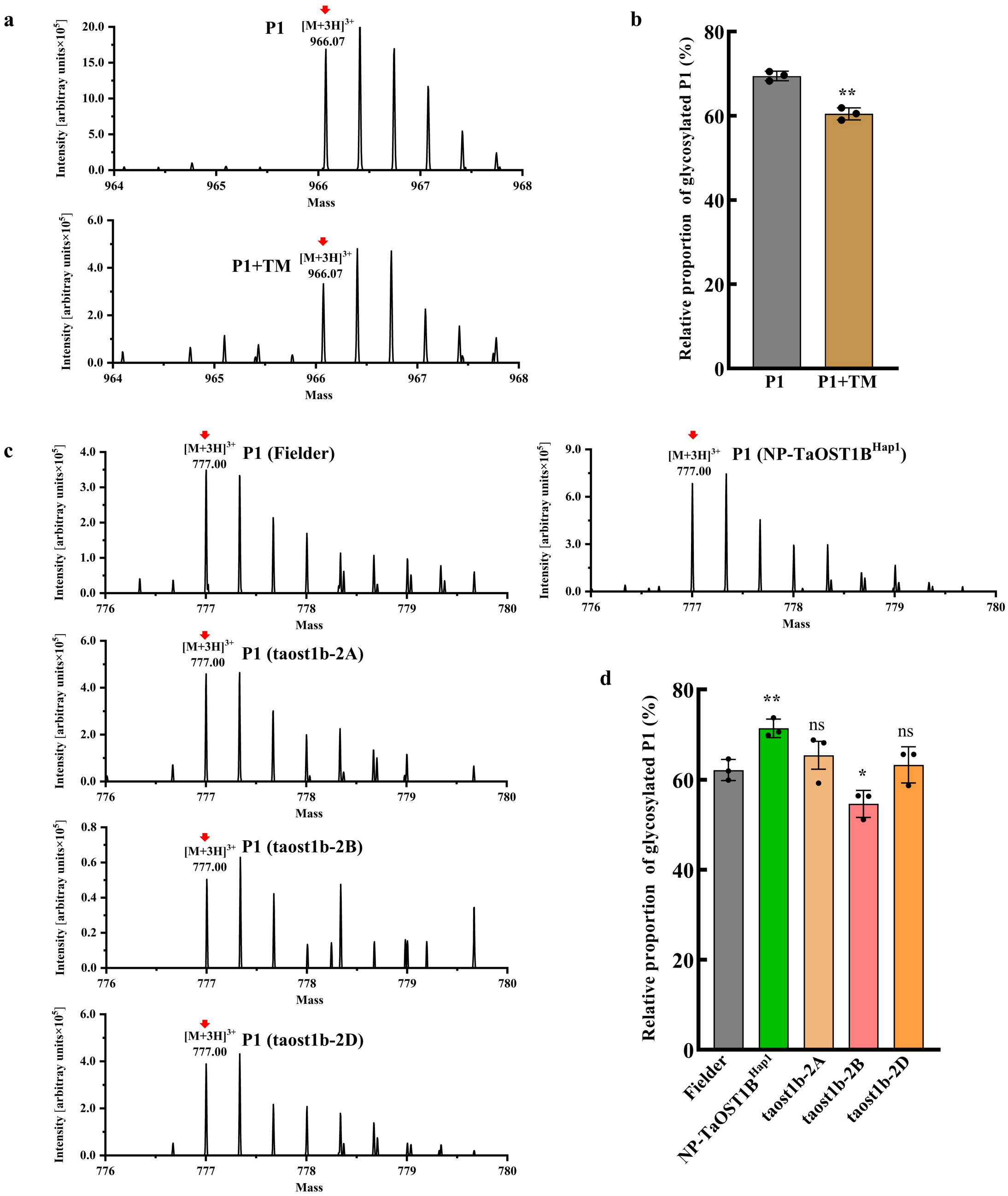

Supplementary Figure 4. The MS1 spectrum of representative intact N-glycopeptide from P1 and the relative comparison analysis for glycopeptides and non-glycopeptides of P1.

(a, c) The MS1 spectrum of representative glycopeptide for Fig. 3F and Fig. 3G. The red arrow indicates the peak related to glycopeptide, YMSNGTHAYEATRL + H(2)N(4)F(1) at m/z 966.07, [M+3H]$^{3+}$ (A); MSNGTHAYEATRL + H(2)N(2)X(1) at m/z 777.00, [M+3H]$^{3+}$ (C). The y axis represents the intensity, the x axis indicates m/z.

(b, d) The relative proportion of glycosylated P1. The intensity of the PNGase F-digested P1 peptides from different samples were examined by LC-MS/MS. The plot is generated by

GraphPad Prism software. The column height represents the intensity of the deamidated P1 peptide divided by the sum of the intensities of the deamidated P1 peptide and the corresponding non-glycosylated peptide. Values are means ± SD (two-sided *t*-test, n = 3 technically independent replicates), ***P* < 0.01, **P* < 0.05, ns *P* > 0.05. Source data are provided as a Source Data file.

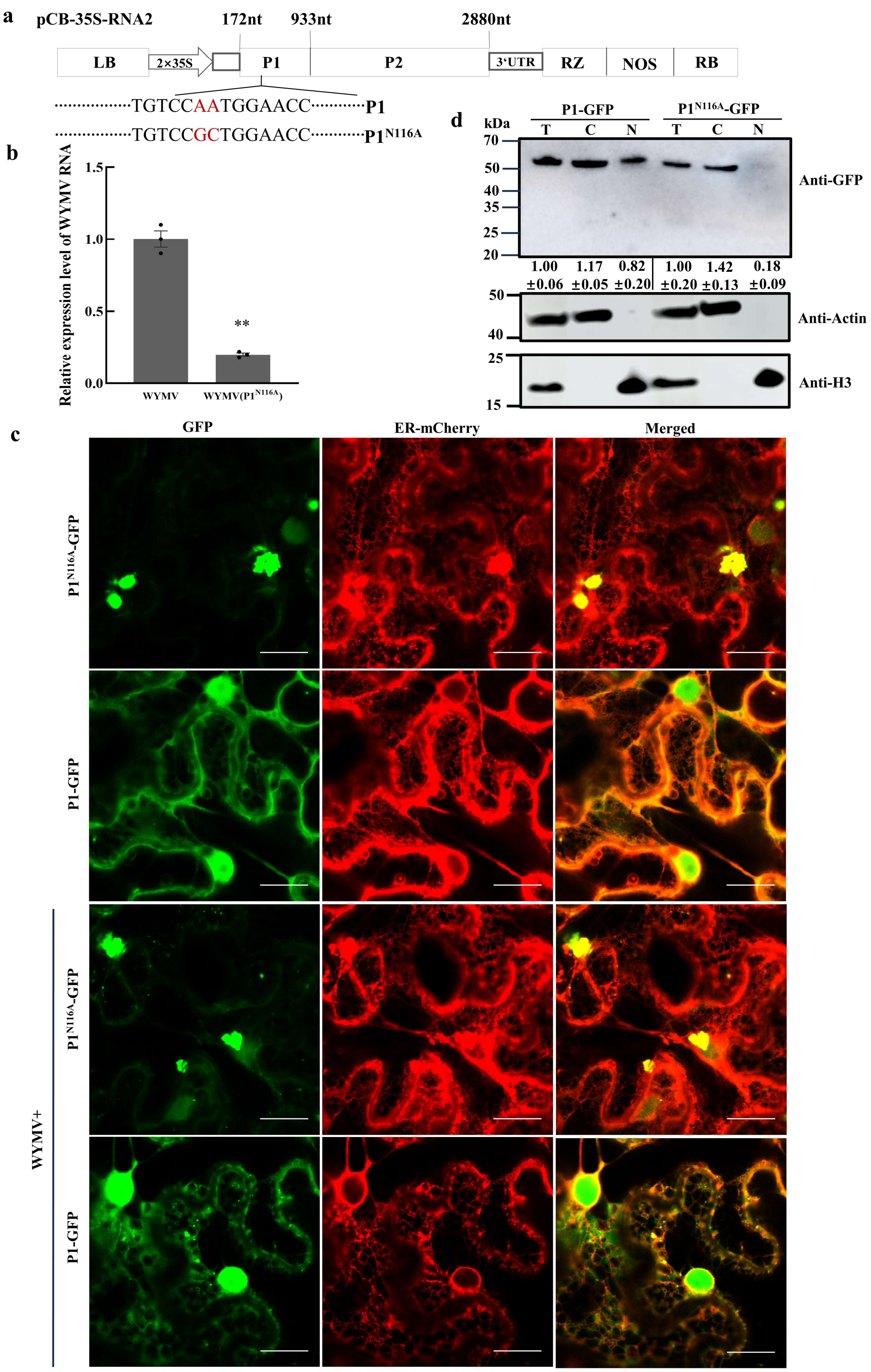

Supplementary Figure 5. (a) Schematic diagram showing the genome organization of pCB-35S-RNA2 and mutant pCB-35S-RNA2($P1^{N116A}$). The difference between the viral genome

is shown in red. **(b)** The accumulation of WYMV RNA in the WYMV- or WYMV(P1^{N116A})-inoculated plants was determined by qRT-PCR. *TaCDC* was used as the internal control gene. Values are means ± SD (two-sided *t*-test, n = 3 biologically independent samples), $^{**}P < 0.01$. **(c)** Colocalization of P1^{N116A}-GFP or P1-GFP and ER in *N. benthamiana* leaves with and without WYMV by confocal microscopy at 48 hpi. Bars indicate 20 μm. **(d)** Western blot analysis of subcellular localizations for the expressed P1-GFP and P1^{N116A}-GFP using an anti-GFP. Each lane represents a sample from an individual experiment, three times the experiment was repeated with similar results. Signal density of protein bands were quantified using Image J software, the values are the means ± SD. T, total protein; C, cytoplasmic protein; N, nuclear protein. Actin was used as a cytoplasmic protein marker, H3 was used as a nuclear protein marker. Source data are provided as a Source Data file.

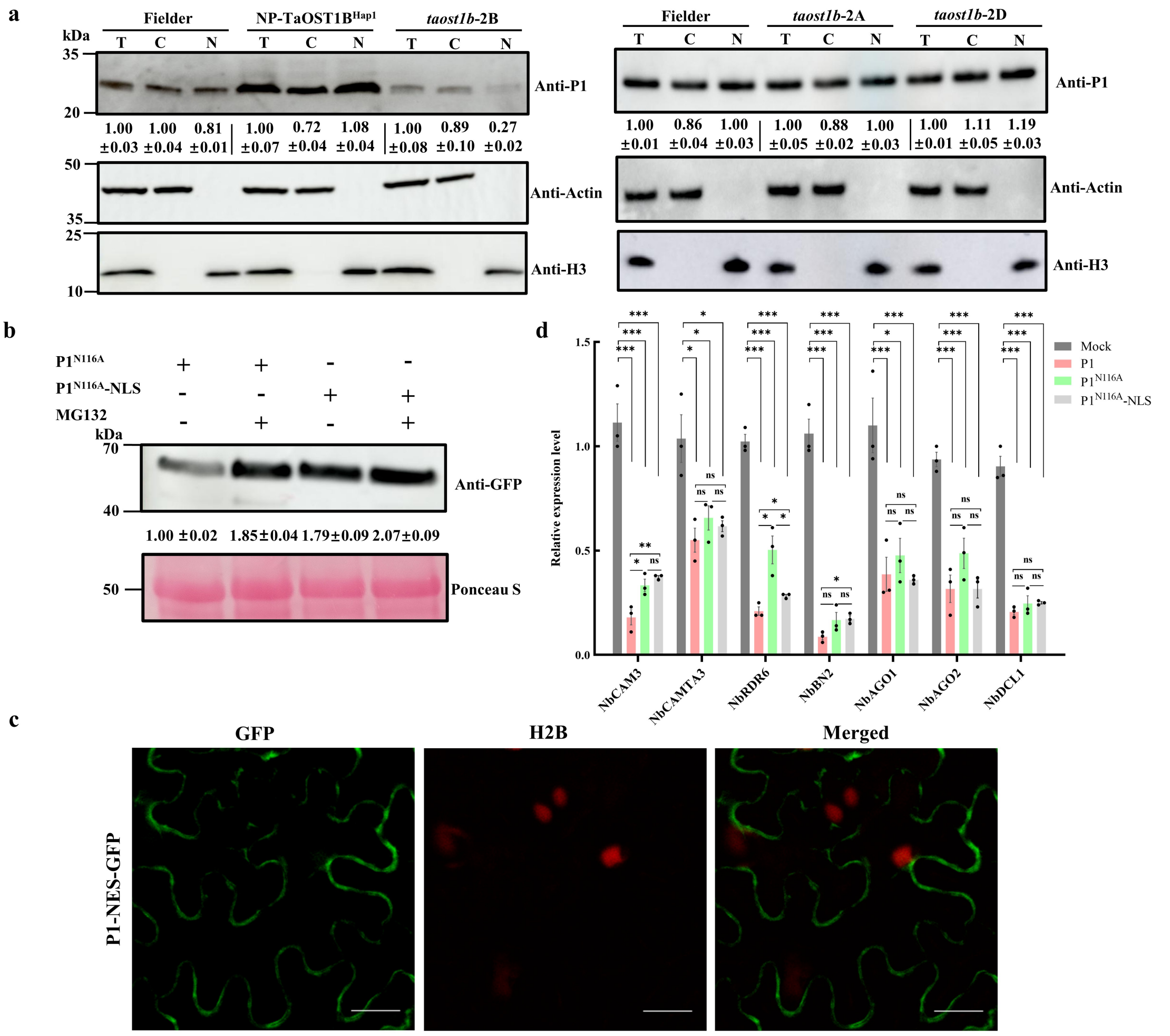

Supplementary Figure 6. (a) Western blot analysis of P1 subcellular localizations in Fielder, NP-TaOST1B^{Hap1}, *taost1b*-2A, *taost1b*-2B, *taost1b*-2D lines under WYMV infection using an specific anti-P1. Each lane represents a sample from an individual experiment, three times the experiment was repeated with similar results. Signal density of protein bands were quantified using Image J software, the values are the means ± SD. Actin was used as a cytoplasmic protein marker, H3 was used as a nuclear protein marker. T, total protein; C, cytoplasmic protein; N, nuclear protein. **(b)** Western blot analysis of accumulation of P1^{N116A} and P1^{N116A}-NLS in Figure 4D. MG132 was added for inhibiting the possible degradation of P1^{N116A}. Each lane represents a sample from an individual experiment, three times the experiment was repeated with similar results. Signal density of protein bands were quantified using Image J software, the values are the means ± SD. Ponceau staining showed samples loading. **(c)** The subcellular localizations of P1-NES-GFP in the leaf cells of H2B-RFP transgenic *N. benthamiana* by confocal microscopy at 48 hpi. Bars indicate 50 μm. **(d)** qRT-PCR analysis of RNAi-related genes expression in *N. benthamiana* leaves expressing P1, P1^{N116A} or P1^{N116A}-NLS. Values are means ± SD (two-sided

t-test, n = 3 biologically independent samples), *$P < 0.05$, **$P < 0.01$, ***$P < 0.001$, ns $P > 0.05$.

Source data are provided as a Source Data file.

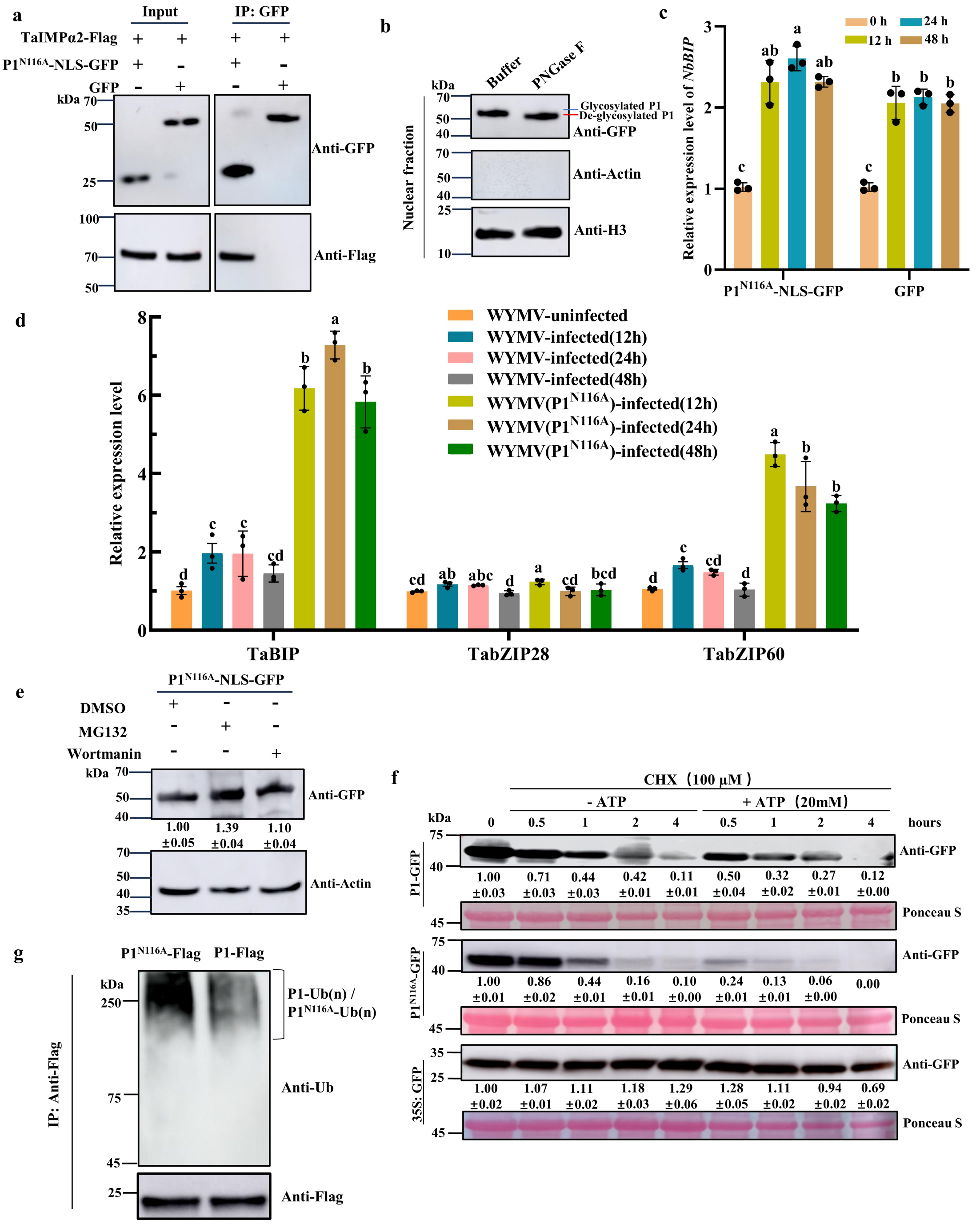

Supplementary Figure 7. (a) Co-IP analysis of the interactions of P1^{N116A}-NLS and TaIMP-α2. TaIMP-α2-Flag with GFP were the negative control groups, and P1^{N116A}-NLS-GFP with TaIMP-α2-Flag were tested. Samples were collected at 2 dpi, following which Co-IP with GFP beads was performed. Three times the experiment was repeated with similar results. **(b)** P1 protein migration analysis of nuclear protein extracts from wheat protoplasts expressing P1-GFP treated with PNGase F glycosidases or a buffer control. Western blotting was performed using GFP antibody. Actin was used as a cytoplasmic protein marker, Histone H3 was used as a nuclear

protein marker. Three times the experiment was repeated with similar results. **(c)** The expression analysis of *NbBIP* ER-stress related representative genes by qRT-PCR in samples expressing $P1^{N116A}$-NLS-GFP for time cross. GFP were used as negative control. *NbActin* was used as the internal control gene. Values are means ± SD (one-way ANOVA tests, n = 3 biologically independent samples), different letters represent significant differences, $P < 0.05$. **(d)** The expression analysis of *TaBIP*, *TabZIP28* and *TabZIP60*, ER-stress related representative genes by qRT-PCR in wheat plants before and after WYMV or WYMV($P1^{N116A}$) inoclulation. *TaCDC* was used as the internal control gene. Values are means ± SD (one-way ANOVA tests, n = 3 biologically independent samples), different letters represent significant differences, $P < 0.05$. **(e)** Effect of MG132 and Wortmannin on the stability of $P1^{N116A}$-NLS-GFP protein expressing in wheat protoplasts by *in vivo* assay. The proteins were extracted at 4 hours when protoplasts were treated with 50 μM MG132, Wortmannin or an equal volume of DMSO (control). Each lane represents a sample from an individual experiment, three times the experiment was repeated with similar results. Signal density of protein bands were quantified using Image J software, the values are the means ± SD. Anti-Actin was used as a protein loading control. **(f)** Detection of the stability of P1 and $P1^{N116A}$ protein by semi-*in vivo* assay. Accumulation levels of P1 and $P1^{N116A}$ were analyzed by western blotting with anti-GFP antibody at different times following 100 μM CHX treatment in the presence or absence of 20 mM ATP. GFP was used as a control. Each lane represents a sample from an individual experiment, three times the experiment was repeated with similar results. Signal density of protein bands were quantified using Image J software, the values are the means ± SD. Ponceau staining shows sample loadings. **(g)** Detection of P1 and $P1^{N116A}$ ubiquitination *in vivo*. P1-Flag or $P1^{N116A}$-Flag were expressed in *N. benthamiana* for 48 hours before immunoprecipitation with anti-Flag magnetic beads. Immunoprecipitated samples were detected with a Flag antibody or a polyclonal antibody against ubiquitin, P1-Ub(n) and $P1^{N116A}$(n) represents ubiquitinationed P1 and $P1^{N116A}$, respectively. Three times the experiment was repeated with similar results. Source data are provided as a Source Data file.

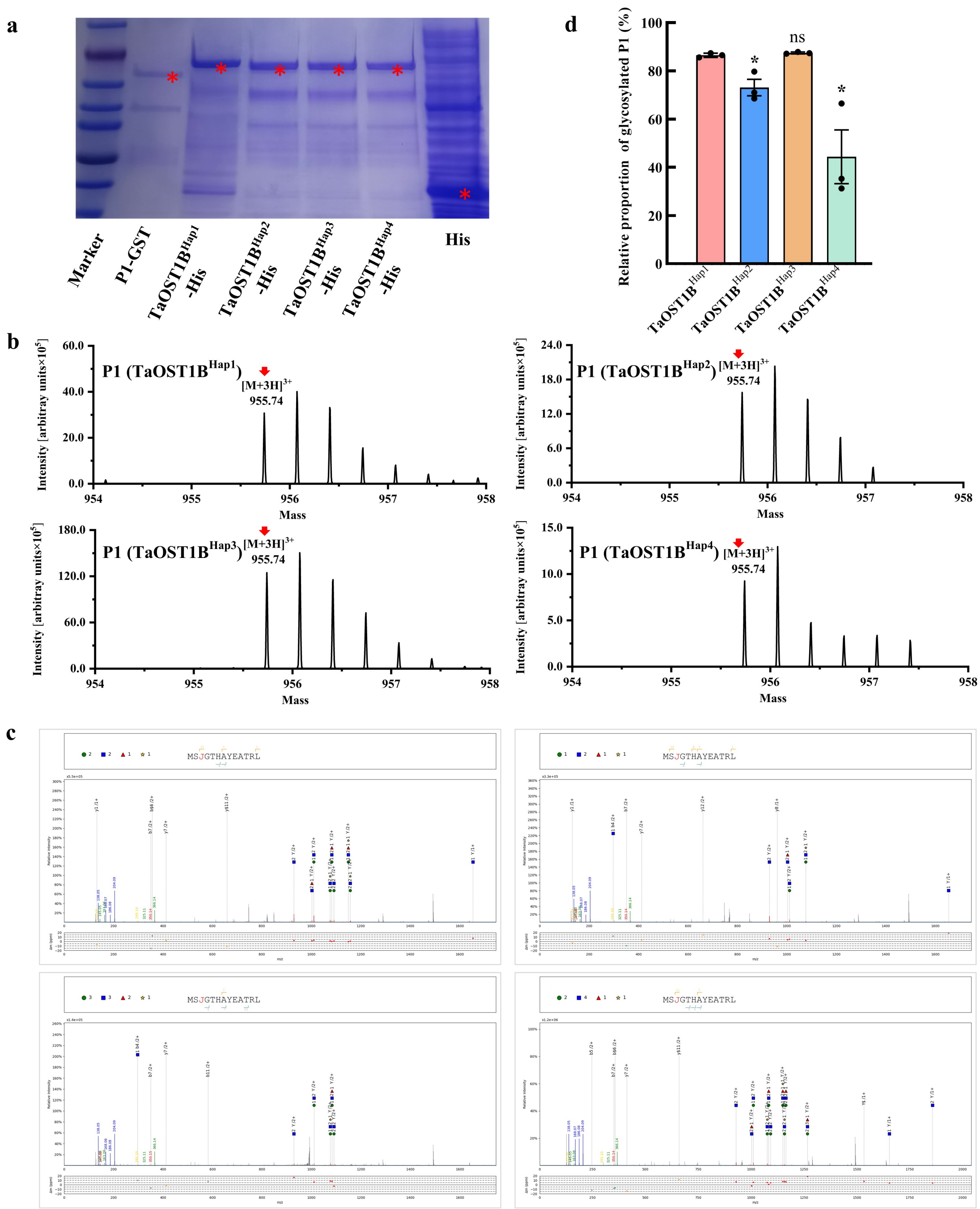

Supplementary Figure 8. (a) Detection of purified P1-GST, TaOST1B^{Hap1}-His, TaOST1B^{Hap2}-His, TaOST1B^{Hap3}-His and TaOST1B^{Hap4}-His proteins. P1-GST were expressed and purified from *E. coli* via GST-binding resin respectively for SDS gel staining. TaOST1B^{Hap1}-His, TaOST1B^{Hap2}-His, TaOST1B^{Hap3}-His and TaOST1B^{Hap4}-His were expressed and purified from *E. coli* via Ni-NTA His-binding resin respectively for SDS gel staining. The red asterisk indicates the size of the corresponding protein. **(b)** The MS1 spectrum of representative glycopeptide for Fig. 7D. The red arrow indicates the peak related to glycopeptide, MSNGTHAYEATRL + H(2)N(4)F(1)X(1) at m/z 955.74, $[M+3H]^{3+}$. The y axis represents the intensity, the x axis indicates m/z. **(c)** The

representative spectra of the intact N-glycopeptide from P1 are provided for qualitative identification of the detected glycoforms, which revealing structural details of the peptide backbone and glycan composition. The red letter "J" indicates the glycosylated asparagine residue. **(d)** The relative proportion of glycosylated P1. The intensity of the PNGase F-digested P1 peptides from different samples were examined by LC-MS/MS. The plot is generated by GraphPad Prism software. The column height represents the intensity of the deamidated P1 peptide divided by the sum of the intensities of the deamidated P1 peptide and the corresponding non-glycosylated peptide. Values are means ± SD (two-sided *t*-test, n = 3 technically independent replicates), $*P < 0.05$, ns $P > 0.05$. Source data are provided as a Source Data file.

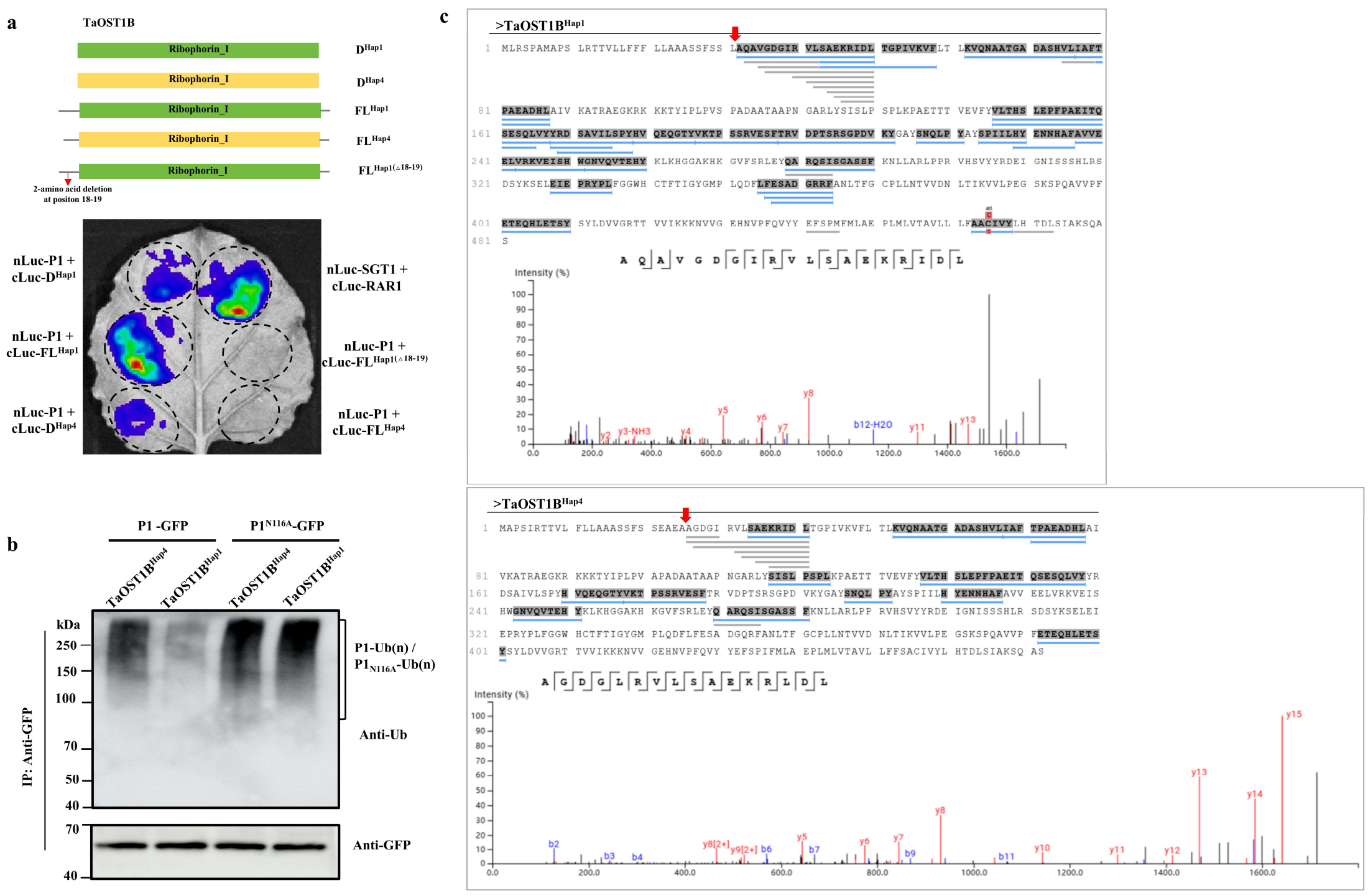

Supplementary Figure 9. (a) LCI assay analysis of 2-amino acid deletion at 18-19 position of TaOST1B on the interaction of P1. The upper pannel showed the structural diagram of the TaOST1B. Luciferase activity was captured using a low-light cooled CCD imaging apparatus at 2 dpi. Three times the experiment was repeated with similar results. **(b)** Detection of P1 and P1^{N116A} ubiquitination *in vivo*. P1-GFP or P1^{N116A}-GFP were expressed in wheat protoplasts with *TaOST1B*^{Hap4} or *TaOST1B*^{Hap1} genotype for 16 hours before immunoprecipitation with anti-GFP magnetic beads. Immunoprecipitated samples were detected with a GFP antibody or a polyclonal antibody against ubiquitin, P1-Ub(n) and P1^{N116A}(n) represents ubiquitinationed P1 and P1^{N116A}, respectively. Three times the experiment was repeated with similar results. **(c)** Immunoprecipitated TaOST1B^{Hap1} and TaOST1B^{Hap4} proteins transiently expressed in *N. benthamiana* were detected by LC-MS/MS. The peptides identified are shown above, and the corresponding ion spectra of the N-terminal peptides of the mature proteins are shown below. The red arrow indicates the site where the signal peptide is cleaved . Source data are provided as a Source Data file.

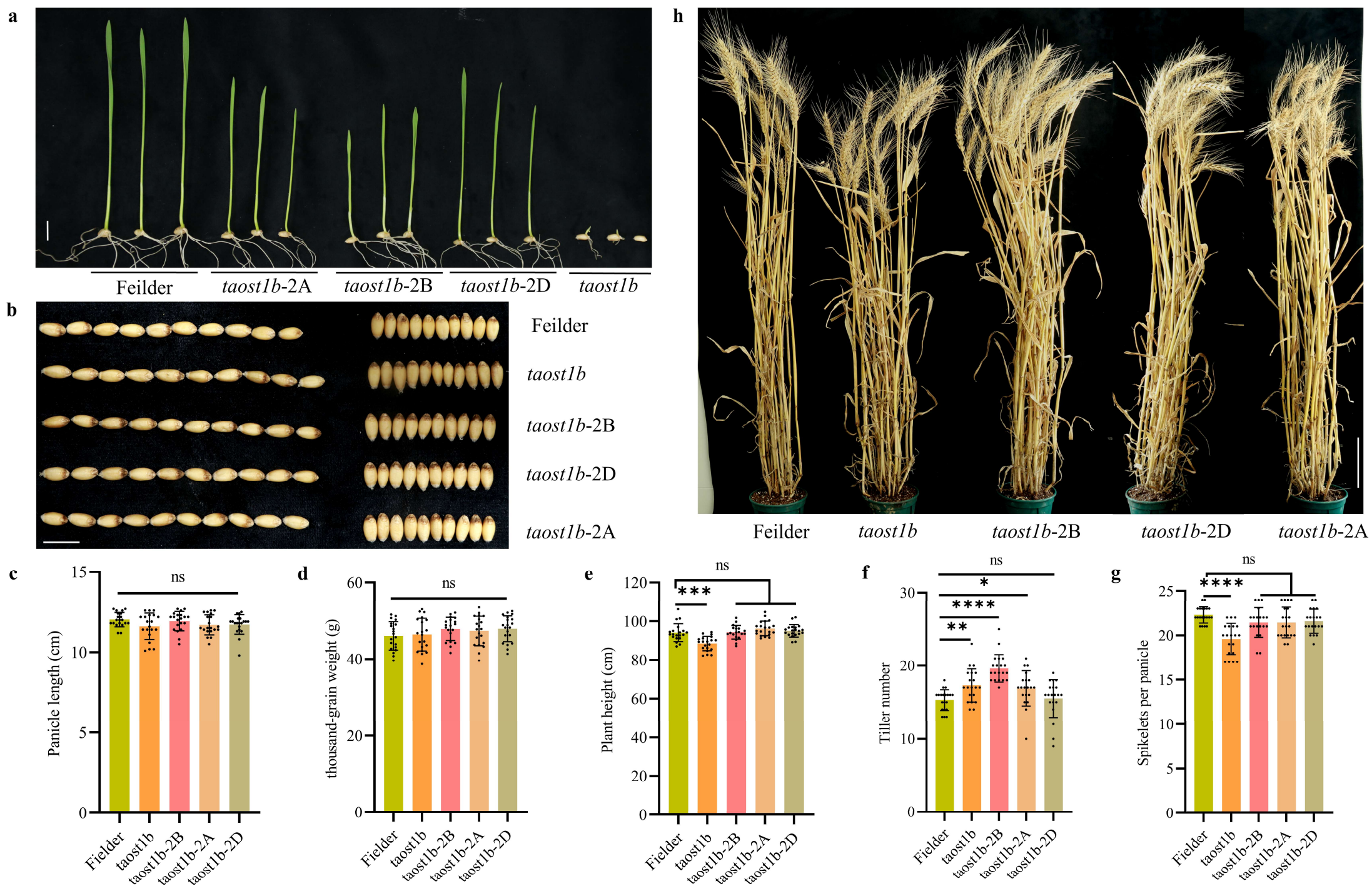

Supplementary Figure 10. Investigation of the agronomic traits of *TaOST1B* knockout lines planted in Yangzhou city, Jiangsu Province. (a) Photograph of seedling stage from Fielder and *taost1b*-2A, *taost1b*-2B, *taost1b*-2D, and *taost1b* lines in controlled environments. Bar indicates 1 cm. **(b)** The length and width of the seeds harvested from field were measured. The seeds were arranged in a row of 10, bar indicates 1 cm. Statistics of **(c)** Panicle length, **(d)** thousand-grain weight, **(e)** Plant height, **(f)** tillers number, and **(g)** spikelets per panicle of Fielder and *taost1b*-2A, *taost1b*-2B, *taost1b*-2D, and *taost1b* lines in the field test. Values are means ± SD (two-sided *t*-test, n = 20 independent plants), $P > 0.05$ (ns), *$P < 0.05$, **$P < 0.01$, ***$P < 0.001$, ****$P < 0.0001$. **(h)** Photograph of maturity from Fielder and *taost1b*-2A, *taost1b*-2B, *taost1b*-2D, and *taost1b* lines from field in pots, bar indicates 10 cm. Source data are provided as a Source Data file.

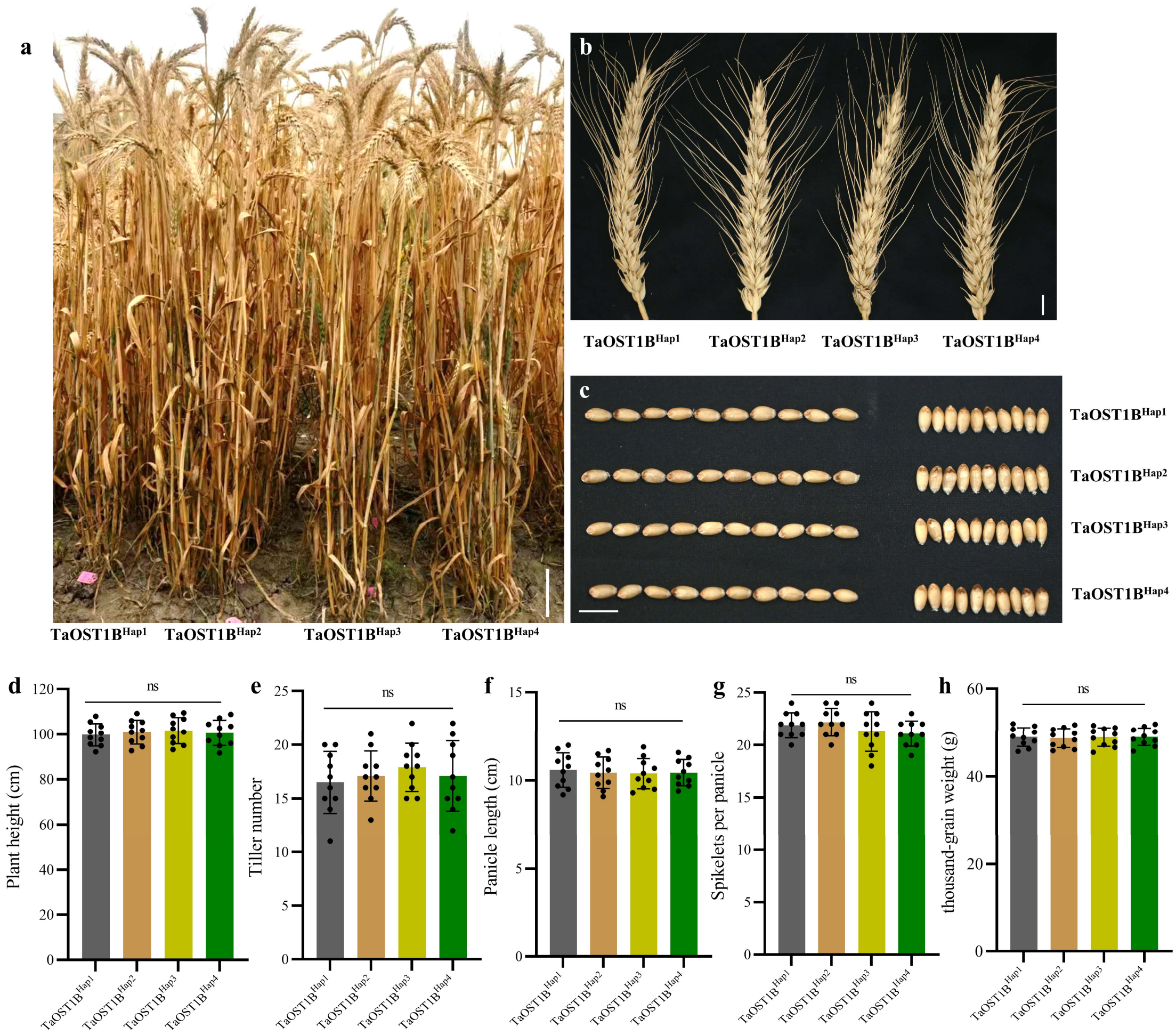

Supplementary Figure 11. Investigation of the agronomic traits of wheat plants corresponding the four haplotypes of TaOST1B at maturity in Yangzhou city, Jiangsu Province. Photograph of maturity **(a)** and spikelets **(b)** from wheat plants corresponding the four haploid of TaOST1B in field. Bars indicate 10 cm (a) and 1 cm (b). **(c)** The length and width of the seeds harvested from field were measured. The seeds were arranged in a row of 10, bar indicates 1 cm. **(d)** Plant height, (**e**) tillers number, (**f**) Panicle length, (**g**) spikelets per panicle, and (**h**) thousand-grain weight of the four haplotypes of TaOST1B lines in the field test (n = 10 independent plants). Values are means ± SD (one-way ANOVA tests, n = 10 independent plants), $P > 0.05$ (ns). Source data are provided as a Source Data file.

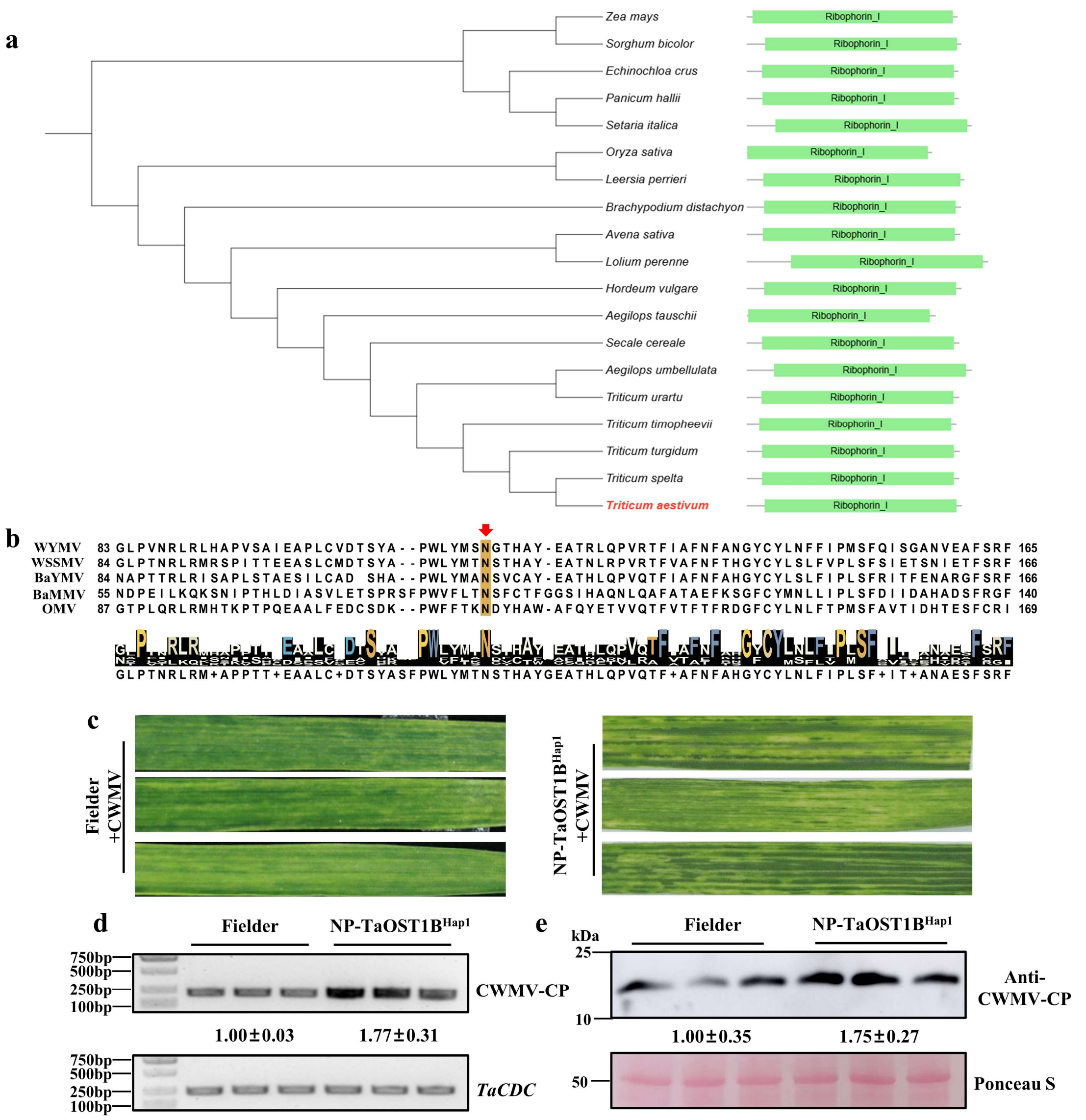

Supplementary Figure 12. (a) The phylogenetic tree and conserved domain analysis of homologous proteins of TaOST1B in the grasses. **(b)** Conserved residues analysis of P1 homologous proteins in WYMV, WSSMV, BaYMV, BaMMV and OMV. The upper pannel was the sequence alignment of P1 homologous proteins, and lower pannel showed the conserved residues. The larger the letter, the more conserved is the amino acid residue at that site. The red arrow indicates the asparagine residue at position 116 of P1. **(c)** The symptom phenotypes of young leaves from the NP-TaOST1B^{Hap1} lines and Fielder wheat plants inoculated with CWMV for 40 days. The semi-quantitative PCR of CWMV RNA accumulation level **(d)** and western blot assay of CWMV CP accumulation level **(e)** were performed for (c). Each treatment included three biological replicates. Signal density of protein bands were quantified using Image J software, the values are the means ± SD. *TaCDC* is a reference gene in wheat. Ponceau staining showed the

samples loading. Source data are provided as a Source Data file.
