# Supplementary material for: The oligosaccharyltransferase TaOST1B promotes viral infection by enhancement of RNA silencing suppression in wheat
Source: Nat Commun. 2026 Jul 15;17:8688. doi: 10.1038/s41467-026-75397-8 (PMC13490409; doi:10.1038/s41467-026-75397-8)
Supplement: Supplementary file 4 [file 41467_2026_75397_MOESM2_ESM.pdf]

Description of Additional Supplementary Files

File Name: Supplementary Data 1

Description: Significant SNPs detected by GWAS in 480 wheat accessions.

File Name: Supplementary Data 2

Description: TaOST1B sequence variations in 363 wheat accessions.

File Name: Supplementary Data 3

Description: Mutation site of TaOST1B in the knockout mutant lines.

File Name: Supplementary Data 4

Description: Predicted N-glycosylation sites in WYMV-encoded proteins.

File Name: Supplementary Data 5

Description: Intensities for P1 glycopeptides and protein in this study.

File Name: Supplementary Data 6

Description: Intensities for deglycosylated and non-glycosylated P1 peptides in this study.

File Name: Supplementary Data 7

Description: Primers used in this study.
